# Renal function following xenon anesthesia for partial nephrectomy—An explorative analysis of a randomized controlled study

**DOI:** 10.1371/journal.pone.0181022

**Published:** 2017-07-18

**Authors:** Ana Stevanovic, Patrick Schaefer, Mark Coburn, Rolf Rossaint, Christian Stoppe, Peter Boor, David Pfister, Axel Heidenreich, Hildegard Christ, Martin Hellmich, Astrid V. Fahlenkamp

**Affiliations:** 1 Department of Anesthesiology, University Hospital RWTH Aachen, Aachen, Germany; 2 Medical Faculty, University of RWTH Aachen, Aachen, Germany; 3 Department of Intensive Care Medicine, University Hospital of RWTH Aachen, Aachen, Germany; 4 Institute of Pathology, University Hospital of RWTH Aachen, Aachen, Germany; 5 Department of Urology, University Hospital of Cologne, Cologne, Germany; 6 Institute of Medical Statistics, Informatics and Epidemiology (IMSIE), University of Cologne, Cologne, Germany; Public Library of Science, FRANCE

## Abstract

**Background:**

Perioperative preservation of renal function has a significant impact on morbidity and mortality in kidney surgery. Nephroprotective effects of the anesthetic xenon on ischemia-reperfusion injury were found in several experimental studies.

**Objective:**

We aimed to explore whether xenon anesthesia can reduce renal damage in humans undergoing partial nephrectomy and to gather pilot data of possible nephroprotection in these patients.

**Design:**

A prospective randomized, single-blinded, controlled study.

**Setting:**

Single-center, University Hospital of Aachen, Germany between July 2013-October 2015.

**Patients:**

Forty-six patients with regular renal function undergoing partial nephrectomy.

**Interventions:**

Patients were randomly assigned to receive xenon- (n = 23) or isoflurane (n = 23) anesthesia.

**Main outcome measures:**

Primary outcome was the maximum postoperative glomerular filtration rate (GFR) decline within seven days after surgery. Secondary outcomes included intraoperative and tumor-related data, assessment of further kidney injury markers, adverse events and optional determination of renal function after 3–6 months.

**Results:**

Unexpected radical nephrectomy was performed in 5 patients, thus they were excluded from the per-protocol analysis, but included in the intention-to-treat analysis. The maximum postoperative GFR decline was attenuated by 45% in the xenon-group (10.9 ml min^-1^ 1.73 cm^-2^ versus 19.7 ml min^-1^ 1.73 cm^-2^ in the isoflurane group), but without significance (*P =* 0.084). Occurrence of adverse events was reduced (*P* = 0.003) in the xenon group. Renal function was similar among the groups after 3–6 months.

**Conclusion:**

Xenon anesthesia was feasible and safe in patients undergoing partial nephrectomy with regard to postoperative renal function. We found no significant effect on early renal function but less adverse events in the xenon group. Larger randomized controlled studies in more heterogeneous collectives are required, to confirm or refute the possible clinical benefit on renal function by xenon.

**Trial registration:**

ClinicalTrials.gov NCT01839084 and EudraCT 2012-005698-30

## Introduction

The worldwide ninth most common type of cancer in men is renal cell carcinoma (RCC), with an increasing incidence [1]. Due to the extended use of surgery, kidney cancer mortality shows a decline in high-resource countries [1]. A partial nephrectomy (PN) with preservation of nephrons is recommended in early-stage RCCs restricted to one kidney [2]. Notwithstanding the nephron-sparing effect of PN compared to radical nephrectomy, a postoperative impairment of renal function is common [3,4]. Despite its often transient nature, a clinically significant GFR decrease of 16–30% is observed in the first days following surgery [3–5]. Early impaired renal function occurs regardless of renal hilar clamping, thus attracting interest to a mechanism of indirect ischemia and reperfusion injury (IRI) involving tumor manipulation and resection itself [3]. Despite protocols to shorten manipulation time, the use of cold ischemia, administration of mannitol, adequate hydration, avoidance of hypotension and blood loss, a postoperative GFR decline cannot fully be prevented [3,5]. After PN the GFR shows an initial nadir, followed by recovery to new reduced baseline levels [4,5]. In elderly patients with pre-existing latent GFR decrease these reduced baseline levels often result in a clinically relevant chronically reduced renal function [4]. Plus, a large body of evidence showed that acute kidney injury (AKI), as induced by IRI, predisposes to chronic kidney disease (CKD) [3,4]. CKD is associated with a significant individual morbidity and mortality, and a high socioeconomic burden. A therapeutic approach enhancing kidney-resilience to IRI would be of outstanding clinical relevance [6].

The noble gas xenon (Xe) is approved for clinical routine use as an inhalational anesthetic with favorable properties [7]. Different experimental *in vitro* and *in vivo* models were able to demonstrate the neuroprotective [8–12] and cardioprotective [13–16] effects induced by Xe treatment. First approaches translating these results into clinical practice showed promising neuroprotective properties of Xe in newborn infants with perinatal asphyxia [17,18] and in patients after out-of-hospital cardiac arrest [19].

Nephroprotection by Xe was recently revealed in both *in vivo* and *in vitro* IRI kidney models [20–22]. Xenon "preconditioning" was identified as one reason for nephroprotection. The underlying mechanisms of Xe "preconditioning" include the activation of HIF-1α [21] microRNA (miR-21) [23] and other cell survival factors like phospho-Akt [22]. They all act via several interrelated pathways enhancing cellular ischemia-tolerance, attenuation of renal tubular damage and apoptosis. Additionally, a nephroprotective effect of Xe by "pre- and postconditioning" was shown in rat models of renal transplantation [24–26]. Xe exposure of donors or recipients induced tubular cell proliferation, reduced cell death and inflammation. This was related to an increased expression of insulin growth factor-1 (IGF-1) and its receptor in human proximal tubular (HK-2) cells, in connection with downstream HIF-1α activation [26]. Clinically, the first hint to improved renal function following Xe anesthesia was found in patients undergoing cardiac surgery [27]. The effects of xenon anesthesia on renal function in patients with kidney surgery have not yet been investigated.

The aim of this explorative study was to explore whether xenon anesthesia can reduce renal damage in humans undergoing PN. The primary outcome variable was chosen to be the maximum post-operative decrease of GFR within the first seven days following surgery. Within this study, we intended to gather preliminary data of potential clinically relevant nephroprotection in the study subjects, comparing the early postoperative reduction of GFR after Xe anesthesia to standard isoflurane (Iso) anesthesia. Secondary outcome variables were further laboratory and clinical marker of kidney function, key anesthesia data, surgical data and malignancy-associated data during the observation period. A long-term follow-up of renal function was obtained after 3–6 months following surgery.

## Materials and methods

### Study design

This study was conducted between July 2013 and October 2015 as a prospective, mono-center, single-blinded, two-arm parallel group randomized controlled study. It was approved by the Ethics Committee of the University of RWTH Aachen, Germany (EK 012/13, 15^th^ April 2013) and the German federal medicines agency (BfArM), and registered on ClinicalTrials.gov (NCT01839084, April 2013). The study was conducted in the University Hospital RWTH Aachen, in adherence to the Declaration of Helsinki. The study protocol has not been published previously. A detailed description of methods is provided as S1 Text. This study is reported in adherence to the CONSORT guidelines. CONSORT checklist is provided in S1 Table. The original German study protocol is provided in S1 Appendix and the English translation in S2 Appendix.

### Participants

After written informed consent, adult patients with a suspected renal carcinoma limited to one kidney and planned for PN were enrolled in this study. Among exclusion criteria were CKD, ASA>III, contraindication and known hypersensitivity to the study drugs, pre-existing severe cardiac disease, severe respiratory disease and neurological disease.

### Randomization and blinding

The randomization sequence based on permuted blocks (allocation ratio 1:1) was computer-generated by an independent statistician (IMSIE, Cologne, Germany) and concealed by means of sealed, opaque envelopes. Only the patient remained blinded during the whole study procedure.

### Changes to methods after trial commencement

A double-blinded (investigator and patient) study procedure was originally planned. During study conduction of the first patients it appeared unfeasible and dispensable to continue the double-blind procedure in clinical routine due to practical differences in anesthesia types and the examiner-independent nature of the primary outcome variable. In agreement with the sponsor and the Ethics Committee the study was continued in a single-blinded manner.

### Intervention

A detailed description is provided in the S1 Text. In summary, all subjects received anesthesia induction with propofol, sufentanil and rocuronium, followed by tracheal intubation and ventilation with a closed-circuit respirator (Felix Dual^™^, Air Liquide Medical Systems, France). The allocated anesthetic agent was administered according to the corresponding randomization envelope. Patients in the Xe group were aimed to receive 60% inspired Xe with 40% oxygen and 1.2% end expiratory Iso with 40% oxygen in the Iso group. Further drugs for maintenance of anesthesia were administered according to the patients’ needs. Two experienced surgeons (AH and DP) performed open PN surgery in accordance with the standard operating procedures of the urology department.

### Primary endpoint

GFR was determined every day and its maximum decrease was determined between the preoperative value and the lowest value within the first seven postoperative days following PN. GFR was calculated by a combined formula incorporating serum creatinine and serum cystatin C: 135 x min(Scr/κ, 1)^α^ x max(Scr/κ, 1^)−0.601^ x min(Scys/0.8, 1)^−0.375^ x max(Scys/0.8, 1)^−0.711^ x 0.995^Age^ [x 0.969 if female] [x 1.08 if black], where Scr is serum creatinine, Scys is serum cystatin C, κ is 0.7 for females and 0.9 for males, α is −0.248 for females and −0.207 for males, min indicates the minimum of Scr/κ or 1, and max indicates the maximum of Scr/κ or 1 [28,29].

### Secondary endpoints

The following secondary endpoints were assessed:

- Patients`vital functions (baseline and daily during the hospital stay until discharge or postoperative day (POD) 7)
- Intraoperative data (surgery day)
- Routine laboratory data including perioperative GFR, cystatin C and creatinine values (baseline and daily during the hospital stay until discharge or postoperative day (POD) 7)
- Tumor-related data (surgery day)
- The kidney injury marker neutrophil gelatinase-associated lipocalin (NGAL) in serum (baseline, intraoperatively 5 minutes after termination of the tumor ground-treatment, in the post anesthesia care unit (PACU) and on POD 1)
- NGAL in urine (baseline, PACU, on POD 1 and 2, and on POD 7 (or at discharge, if earlier))
- Occurrence of adverse events (surgery day until discharge (if earlier) or POD 7)
- Optional follow-up of renal function after 3–6 months.

### Outcome measures

All patients underwent a maximum of 11 visits. The study procedure is summarized in the S1 Fig. and described more extensively in the S1 Text. In brief, the visits consisted of: Visit 0 (preoperative baseline visit), visit 1 (intraoperative visit), visit 2 (postoperative visit on the surgery day), visit 3 (POD 1), visit 4 (POD 2), visit 5 (POD 3), visit 6 (POD 4), visit 7 (POD 5), visit 8 (POD 6), visit 9 (POD 7), visit 10 (optional, 3–6 months after surgery).

### Study sample size calculation

According to the literature [5] and our clinical experience, we assumed up to 30% decrease in GFR (corresponding to 54 ml min^-1^ 1.73^−1^ m^-2^) in a control group patient with normal preoperative renal function (180 ml min^-1^ 1.73^−1^ m^-2^). A 10% reduction of the postoperative GFR decrease in the Xe group was considered as clinically significant. This would correspond to a GFR decrease of 36 ml min^-1^ 1.73^−1^ m^-2^. Assuming a large standardized effect of 0.85, the t-test required 23 patients per group to reach 80% power at two-sided significance level 5%. Expecting a dropout rate of 10%, we have decided to include 2 more patients per group, which yielded 25 patients per group in total.

### Statistical methods

The primary endpoint—maximum decrease of the GFR in the first seven days following PN—was evaluated by analysis of covariance adjusted for the baseline value. Secondary endpoints were evaluated with Fisher's exact test (qualitative data) or Mann-Whitney *U*-test (quantitative data). GraphPad PRISM^®^ (GraphPad Software Inc., La Jolla, California, USA) was used to create figures. All data were analyzed on intention-to-treat (ITT) basis, including all randomized patients, and on per-protocol (PP) basis, excluding subjects with unexpected radical nephrectomy. Statistical calculations were performed with the software SPSS Statistics (version 23; IBM Corp., Armonk, NY, USA).

## Results

**Fig 1:**
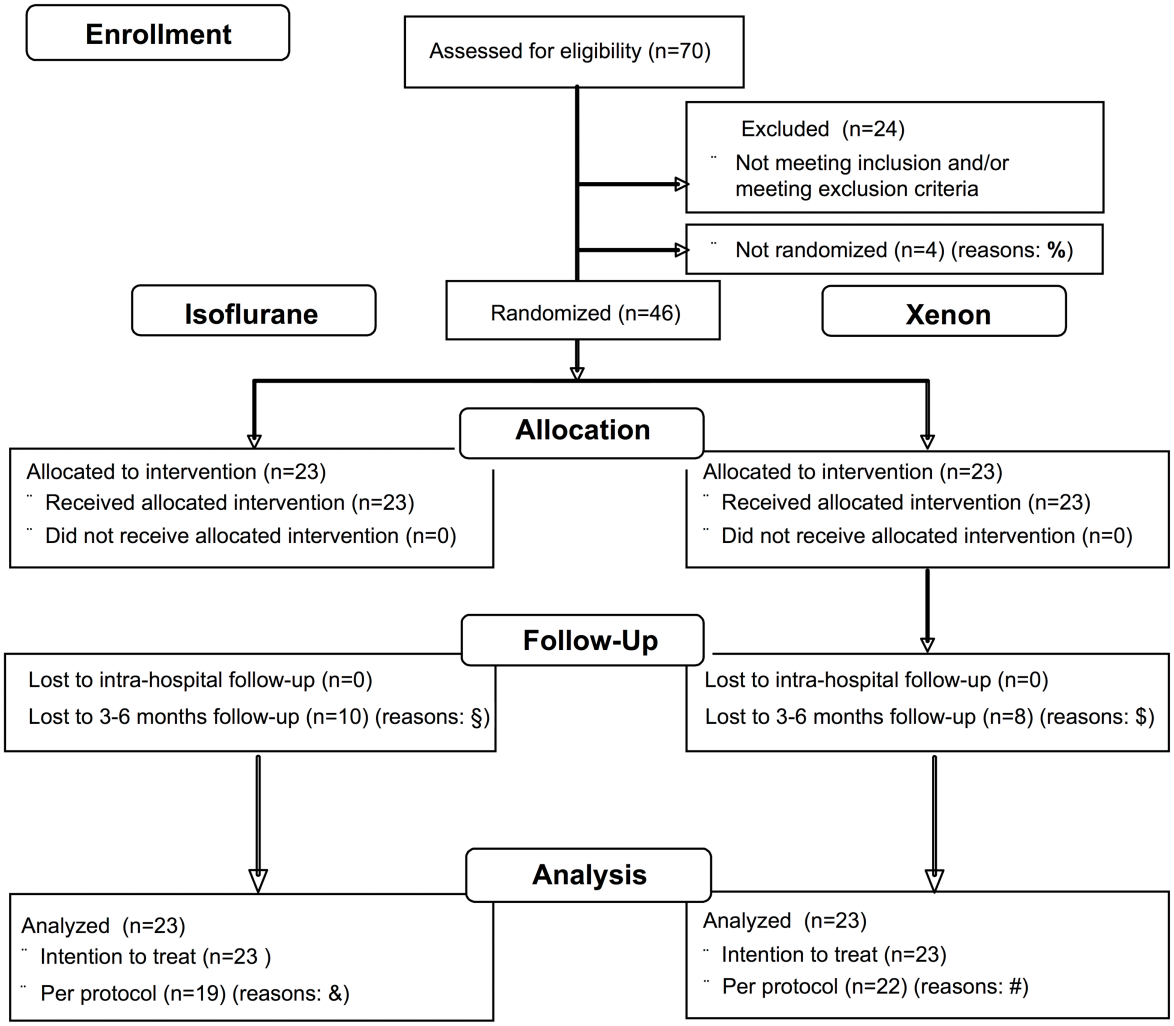
CONSORT-Flowchart. The CONSORT-Flowchart depicts the study-flow from screening until the analysis. Reasons for patient loss: ^%^4 patients were not randomized due to the unplanned earlier stopping of our study after our urological surgeons have left the institution. ^§^(n = 10) primary physician did not control the renal function until 6 months after surgery. ^$^(n = 7) primary physician did not control the renal function until 6 months after surgery, (n = 1) patient deceased within 2 months after surgery, due to another disease. ^&^(n = 4) patients received total nephrectomy. ^#^(n = 1) patient received total nephrectomy.

A detailed study flow chart is presented in Fig 1. 46 subjects were enrolled and equally randomized into the treatment- (n = 23 Xe) and control-group (n = 23 Iso). No patient was excluded from final ITT analysis. Five patients received an unexpected radical nephrectomy (Iso (n = 4) and Xe (n = 1)) due to medical reasons and were excluded from PP analysis. Thus, we included 19 Iso patients and 22 Xe patients into PP analysis. Outcome results, which are presented in our tables and figures, show the ITT analysis data as well as the PP analysis data. All subjects completed primary endpoint analysis. Given the optional character of our follow-up analysis; 18 patients were lost to follow-up for GFR determination after 3–6 months.

### Recruitment

Subjects were recruited between 07/2013 and 10/2015, and the last follow-up was conducted in February 2016. Due to the unexpected leaving of both urological surgeons (Head of Department AH and DP) we had to stop subject recruitment earlier than expected, as this procedure was not performed any longer in the same way. At this time only 46 patients were included, which was the minimal subject number to achieve the estimated power. After discussion with all investigators and the sponsor, we decided to stop recruitment since comparable study conditions were no longer ensured.

### Baseline data

Patient baseline characteristics (Table 1) and pre-existing chronic diseases (S2 Table) did not show any significant difference between the groups, except for the variable urea.

**Table 1:** Patient baseline characteristics.

Group	Total (n = 46)	Isoflurane (n = 23)	Xenon (n = 23)	*P-*value^a^
Sex: m/f [n] (%)	32/14 (69.9/30.4)	15/8 (65.2/34.8)	17/6 (73.9/26.1)	0.749
Age [yrs.]	60.2 ± 14.2, 59.5 (19)	60.0 ± 14.6, 61 (23)	60.4 ± 14.2, 59 (20)	0.912
Height [cm]	173 ± 9.2, 173 (21)	173 ± 8.7, 175 (12)	173 ± 9.8, 172 (14)	0.991
Weight [kg]	81.4 ± 14.9, 82 (24)	81.1 ± 16.1, 82 (26)	81.7 ± 14.0, 82 (23)	0.834
ASA I/II/III [n] (%)	4/31/11 (8.7/67.4/23.9)	2/17/4 (8.7/73.9/17.4)	2/14/7 8.7/60.9/30.4)	0.663
Preoperative baseline values of renal function variables				
Baseline GFR [ml min^-1^ 1,73 cm^-2^]	88.5 ± 16.0, 87.9 (18.8)	90.2 ± 14.0, 85.6 (16)	86.7 ± 17.8, 88.0 (26.4)	0.668
Baseline creatinine [mg dl^-1^]	0.9 ± 0.2, 0.9 (0.2)	0.8 ± 0.1, 0.8 (0.1)	0.9 ± 0.2, 0.9 (0.2)	0.169
Baseline cystatin C [mg dl^-1^]	0.9 ± 0.2, 0.9 (0.3)	0.9 ± 0.2, 0.9 (0.3)	1.0 ± 0.2, 0.9 (0.3)	0.553
Baseline urea [mg dl^-1^]	31.8 ± 7.6, 32 (9)	28.3 ± 7.8, 30 (7)	34.9 ± 6.1, 34.5 (8.5)	0.023*
Baseline NGAL in serum [ng ml^-1^]	23.9 ± 7.6, 23.6 (10.6)	24.0 ± 7.1, 21.6 (12)	23.8 ± 8.2, 24.7 (10.6)	0.869
Baseline NGAL in urine [ng ml^-1^]	5.9 ± 9.3, 2.8 (4.8)	7.3 ± 11.1, 3.7 (6)	4.5 ± 7.0, 2.5 (3.2)	0.067

ASA, American Society of Anesthesiologists; GFR, glomerular filtration rate; n, number; NGAL, neutrophil gelatinase-associated lipocalin

^a^*P*-values are from Fisher's exact test (qualitative data) or Mann-Whitney *U*-test (quantitative data), respectively.

*Significant *P*-value <0.05.

Data are presented as mean ± standard deviation, median (interquartile range) or number and percentage.

### Primary outcome

**Fig 2:**
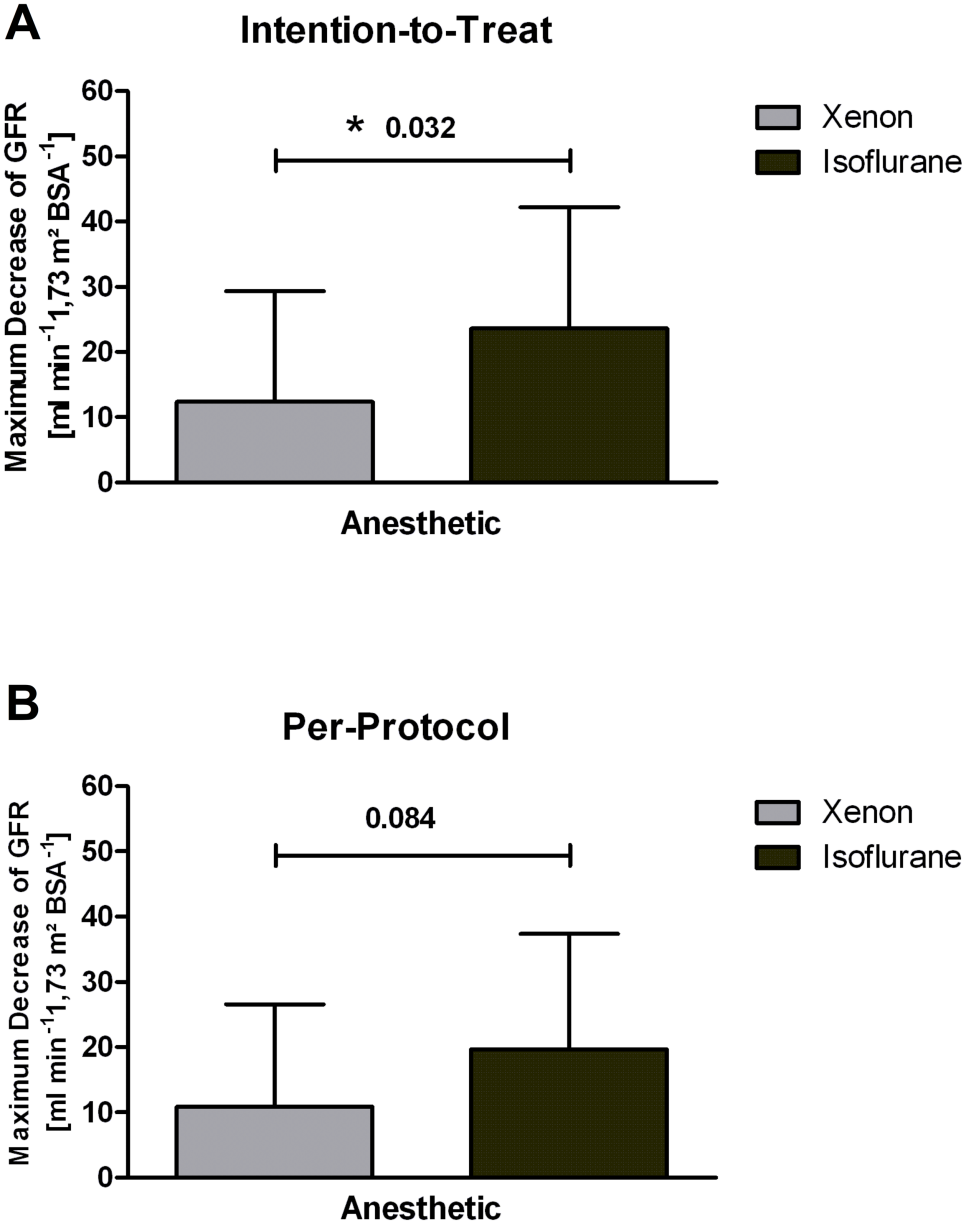
Maximum glomerular filtration rate decrease within 7 days after renal surgery. (A) Intention-to-Treat analysis of the maximum early glomerular filtration rate. (B) Per-protocol analysis of the maximum early glomerular filtration rate in patients undergoing partial nephrectomy. Five patients were excluded from this analysis due to radical nephrectomy. Data are means ± standard deviation. * Significant *P*-value. BSA, body surface area; GFR, glomerular filtration rate.

The sample size for the PP analysis of the primary outcome included 19 patients in the Iso group and 22 patients in the Xe group. Xe reduced the maximum postoperative GFR decrease by 45% compared to Iso in the PP analysis (*P* = 0.084) (Fig 2A). The maximum GFR decrease was 10.9 ml min^-1^ 1.73 m^-2^ in the Xe group, and 19.7 ml min^-1^ 1.73 m^-2^ in the Iso group (S3 Table). Of note, the ITT analysis showed a significant *P*-value of 0.032 (Fig 2B).

### Secondary outcomes

#### Kidney function and markers of kidney injury

**Fig 3:**
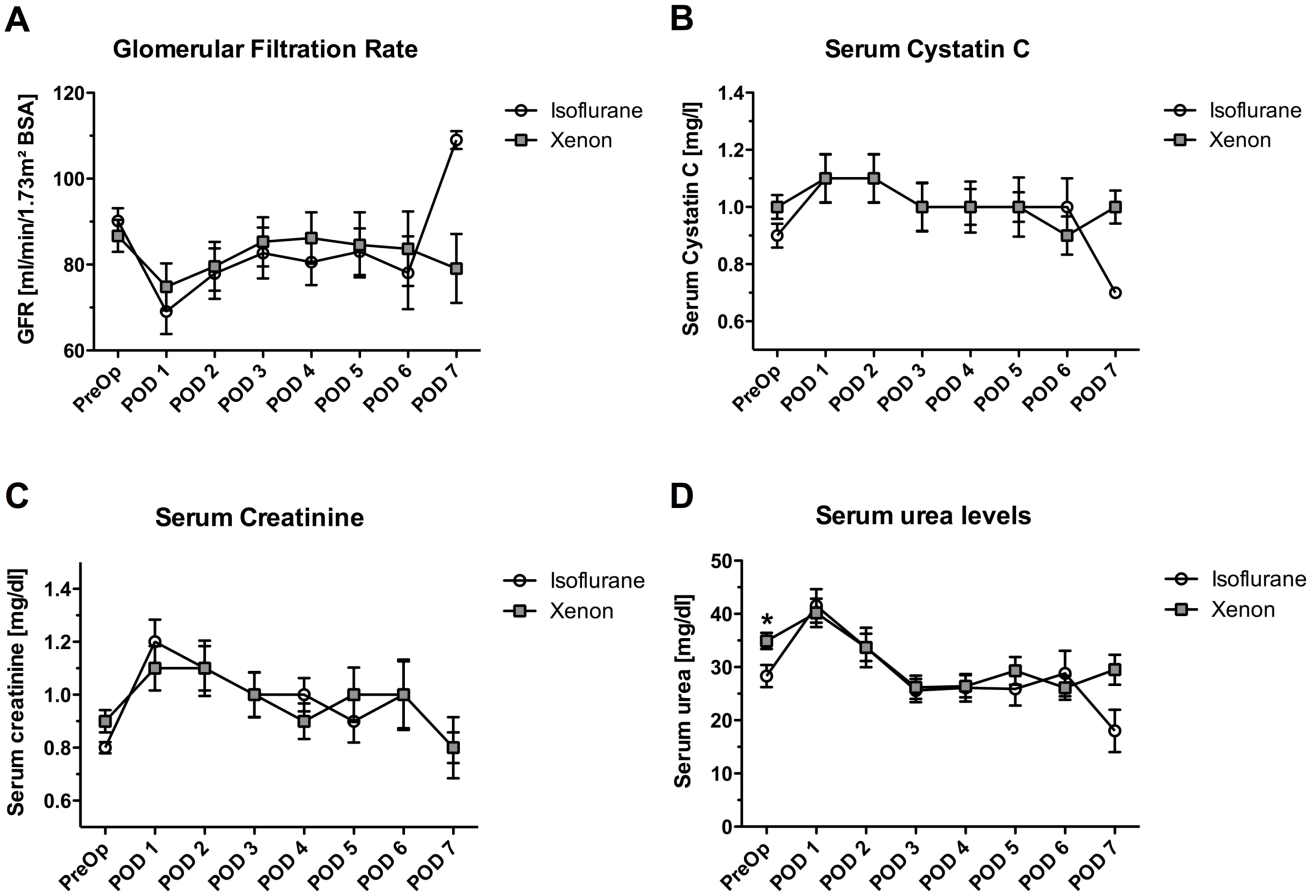
Early postoperative renal function. (A) Perioperative glomerular filtration rate values (GFR) from preoperative until the 7^th^ postoperative day. The respective *P*-values for the particular time-points were: *P* = 0.464 preoperatively, *P =* 0.715 POD 1, *P* = 0.507 POD 2, *P* = 0.703 POD 3, *P* = 0.988 POD 4, *P* = 0.959 POD 5, *P* = 0.908 POD 6, and *P* = 0.050 POD 7. (B) Perioperative serum cystatin C values from preoperative until the 7^th^ postoperative day. The respective *P*-values for the particular time-points were: *P* = 0.339 preoperatively, *P* = 0.914 POD 1, *P* = 0.533 POD 2, *P* = 0.673 POD 3, *P* = 0.815 POD 4, *P* = 0.481 POD 5, *P* = 0.685 POD 6, and *P* = 0.050 POD 7. (C) Perioperative serum creatinine values from preoperative until the 7^th^ postoperative day. The respective *P*-values for the particular time-points were: *P* = 0.150 preoperatively, *P* = 0.774 POD 1, *P* = 0.308 POD 2, *P* = 0.334 POD 3, *P* = 0.861 POD 4, *P* = 0.354 POD 5, *P* = 1.0 POD 6, and *P* = 0.513 POD 7. (D) Perioperative serum urea values from preoperative until the 7^th^ postoperative day. The respective *P*-values for the particular time-points were: *P* = 0.007 preoperatively, *P* = 0.792 POD 1, *P* = 0.188 POD 2, *P* = 0.649 POD 3, *P* = 0.608 POD 4, *P* = 0.230 POD 5, *P* = 0.713 POD 6, and *P* = 0.076 POD 7. Of note, early postoperative kidney function was analyzed until the 7^th^ postoperative day or until discharge, if earlier. Data on the 7^th^ postoperative day were only available for 3 patients of each group. Data are means ± standard deviation. * *P*-value <0.05. POD, postoperative day; PreOP, preoperative baseline values.

**Fig 4:**
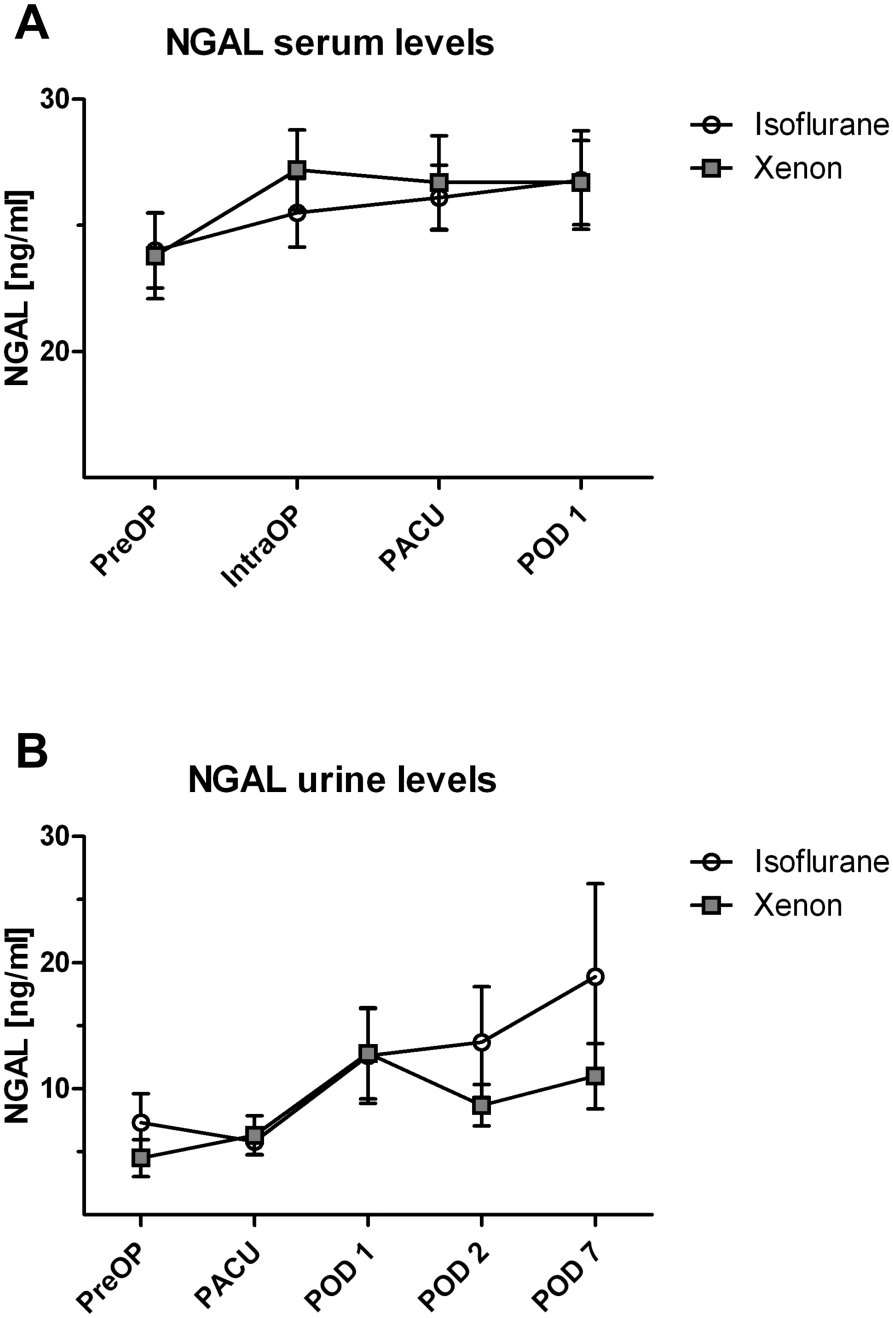
Early postoperative course of NGAL in serum and urine. (A) NGAL concentrations in serum were assessed from preoperative until the first postoperative day. There was no statistically significant difference between the groups for each time-point (*P* = 0.958 preoperatively, *P* = 0.239 intraoperatively, *P* = 0.896 in PACU, and *P* = 0.979 on POD 1). (B) NGAL concentrations in urine were assessed from preoperative until the postoperative day 7. There was no statistically significant difference between the groups for each time-point (*P* = 0.053 preoperatively, *P* = 0.860 in PACU, *P* = 0.875 on POD 1, *P* = 0.273 on POD 2, and *P* = 0.627 on POD 7). Data are means ± standard deviation. IntraOP, intraoperative; NGAL, neutrophil gelatinase-associated lipocalin; PACU, post anesthesia care unit; POD, postoperative day; preOP, preoperative baseline values.

The sample size for the PP analysis of the kidney-related secondary outcomes comprised 19 patients in the Iso group and 22 patients in the Xe group. GFR showed the maximum decrease on POD 1, followed by a recovery to a new reduced baseline level until POD 7 in both groups (Fig 3A). Time-line of serum creatinine, cystatin C, and urea showed an initial increase on the POD 1, which was followed by a slower decrease back to baseline values until POD 7 in both groups (Fig 3B–3D). Serum and urine NGAL showed a postoperative increase, which did not significantly differ between the groups (Fig 4A + 4B).

#### Anesthesia and surgery-related data

The sample size for the anesthesia and surgery-related data in the PP analysis consisted of 19 patients in the Iso group and 22 patients in the Xe group. Intraoperative anesthesia-related data were collected during visit 1. Blood pressure was measured every 5 minutes; anesthetic gas and oxygen concentrations as well as the anesthesia depth and heart rate were measured continuously and documented every 5 minutes. We revealed a significant difference in inspired oxygen concentration and the mean systolic and diastolic blood pressure in both statistical analyses (Table 2). Patients in the Xe group received less inspired oxygen and the mean systolic and diastolic blood pressures were significantly higher in the Xe group. Anesthesia depth, measured with bispectral index (BIS) monitoring was within the recommended margin of 40–60 in both groups (Table 2).

**Table 2:** Anesthesia-related data.

Analysis	Intention to Treat			Per Protocol		
Group	Isoflurane (n = 23)	Xenon (n = 23)	*P*-value^a^	Isoflurane (n = 19)	Xenon (n = 22)	*P*-value^a^
Duration of anesthesia [min]	191.8 ± 59.5, 176 (102)	162.0 ± 59.2, 153 (93)	0.093	191.4 ± 60.6, 176 (112)	160.1 ± 59.9, 147.5 (89)	0.102
Mean inspired oxygen [%]^b^	49.7 ± 6.6, 49.1 (10.1)	44.4 ± 4.6, 44.3 (5.5)	0.010	49.0 ± 6.5, 47.8 (10.9)	44.3 ±4.7, 44.2 (5.5)	0.028
Mean anesthetic concentration [%] (MAC)^b^	0.8 ± 0.1 (0.7 MAC), 0.8 (0.2)	51.1 ± 2.5 (0.8 MAC), 51.4 (2.9)	-	0.8 ± 0.1 (0.7 MAC), 0.9 (0.2)	51.1 ± 2.5 (0.8 MAC), 51.5 (2.9)	-
Mean RR sys [mmHg]^b^	107.7 ± 7.7, 105.7 (12.1)	116.9 ± 14.4, 114.2 (26.9)	0.020	106.9 ± 7.4, 104.4 (12)	117.5 ± 14.4, 114.6 (26.4)	0.012
Mean RR dia [mmHg]^b^	64.5 ± 9.2, 64 (11.6)	73.9 ± 12.3, 75.1 (19.3)	0.011	63.6 ± 8.5, 64 (11.5)	74.8 ± 11.7, 76.2 (18.6)	0.003
Mean heart rate [bpm]^b^	65.2 ± 9.1, 65.7 (12.7)	61.6 ± 13.2, 57.9 (15.4)	0.116	64.0 ± 7.4, 63.7 (12.7)	61.8 ± 13.5, 57.5 (15.4)	0.182
Mean BIS^b^	49.2 ± 5.2, 48.6 (7.5)	48.9 ± 4.3, 48.9 (4.3)	0.752	49.7 ± 5.6, 49.4 (8.6)	48.8 ± 4.4, 48.8 (4.3)	0.615

BIS, bispectral index; bpm, beats per minute; MAC, minimum alveolar concentration; n, number; RR sys, systolic blood pressure; RR dia, diastolic blood pressure.

^a^ *P*-values are from Mann-Whitney *U*-test.

^b^ Data were recorded every 5 minutes during anesthesia.

Data are presented as mean ± standard deviation, median (interquartile range).

Surgery data were similar for both groups, with the exception of anesthetics exposure time before kidney manipulation, which was significantly prolonged in the Iso group (S4 Table). No difference was found for surgery duration, kidney manipulation and hilar clamping time, anesthetic exposure time after kidney manipulation, intraoperative fluid input (crystalloids and colloids) and output (urine and blood loss). Hilar clamping was performed in 17 patients (Iso (n = 8) and Xe (n = 9)). The mean clamping duration was longer in the Xe group (S4 Table). The maximum postoperative GFR decrease was higher in all patients with hilar clamping. Of note, patients with Xe anesthesia had less GFR decrease compared to the Iso group even after hilar clamping (S3 Table)

Histological (tumor classification) and pathological (renal tissue excision volume, size and weight) analysis did not reveal any significant differences between the groups (S5 Table).

#### Adverse events

Occurrence of adverse events was significantly lower in the Xe group (*P* = 0.003) (S6 Table). The sample size in the PP analysis contained 19 patients in the Iso group and 22 patients in the Xe group. Considering the individual events, there was a significant difference for intraoperative hypotension requiring catecholamine support in the Iso group compared to Xe. A significantly increased incidence of anemia in the Iso group, due to postoperative bleeding via surgical drain or hematoma, was only present in the PP analysis. Nausea, vomiting, AKI assessed according to the Acute Kidney Injury Network (AKIN) classification [30] and the length of in-hospital stay were similar in both groups (S6 and S7 Tables).

#### Longer-term kidney function

The optional longer-term analysis of kidney function was obtained after 3–6 months for 28 patients (Fig 1 and S7 Table). The PP analysis included 11 patients in the Iso group and 15 patients in the Xe group Renal function did not show any significant difference between the two patient groups and revealed a mean GFR of 78.9±16.6 ml min^-1^ 1,73m^-2^ in the Iso and 82.1±16.0 ml min^-1^ 1,73m^-2^ in the Xe group.

## Discussion

The present study is the first randomized clinical study, which could confirm that Xe anesthesia is feasible and safe in patients undergoing PN surgery with regard to the postoperative renal function. We could not confirm preclinical results that report better kidney function in mice after exposure to Xe anesthesia and kidney IRI [21]. The result of a significantly attenuated GFR decrease was only found in the ITT analysis, whereas it did not differ in the PP analysis (*P* = 0.084). The most likely reason for this difference between the ITT and PP analysis is the small sample size of our study and the unfortunately required exclusion of 5 nephrectomy patients from the PP analysis. The decision to perform a radical nephrectomy was not dependent on the type of anesthetic, but on the anatomical size and condition of the tumor. This decision was made during surgery and could not be foreseen at patient recruitment. Patients with nephrectomies were excluded from the PP analysis, as the aim of this study was to preserve nephron function in surgery-related IRI by Xe, which is disrupted by nephrectomy. This issue most likely hindered a powerful primary endpoint result. Therefore, we cannot prove if Xe is nephroprotective and if we would have the same result with a higher PP sample size. On the other hand, it remains controversial if the 4 patients with nephrectomies in the Iso group versus 1 nephrectomy patient in the Xe group were exclusively decisive for the significant difference in the ITT, due to the greater decline in GFR after nephrectomy independently of the used anesthetic.

Of note, we found a trend of reduced GFR decrease in the Xe group, despite the significantly longer Iso exposure before tumor manipulation, and despite a higher rate of hilar clamping with Xe.

A further important factor for preservation of postoperative organ function is the maintenance of intraoperative hemodynamic stability, which was more present in the Xe group. Therefore, it remains unclear if Xe directly induces kidney function preservation or indirectly via more stabilized blood pressure during anesthesia. But from the clinical point of view, both would be beneficial. Of note, almost 50% of our patients (n = 12 in each group) had pre-existent arterial hypertension. Especially, these patients may profit from higher and more stable intraoperative blood pressure, as their auto regulation of renal perfusion is usually shifted to a higher range [31]. We have chosen Iso as the comparative, as it was shown to have the strongest nephroprotective effect against IRI among commonly used volatile anesthetics [32–34]. Furthermore, Iso does not exhibit potentially nephrotoxic effects like sevoflurane [35], and it was not inferior in clinical studies examining postoperative renal function after major surgery and renal transplantation compared to sevoflurane, desflurane or propofol anesthesia [36–38].

In contrast to one large randomized clinical study comparing Iso and Xe anesthesia, we did not reveal a significant difference in mean heart rates [39]. Whereas Wappler et al. included only ASA I-II patients, we have also included eleven ASA III patients in our study. Of note, the applied minimal alveolar concentration (MAC) could be a potential confounder for hemodynamic outcomes, but data regarding the MAC values for Xe are controversial [40]. Therefore, our MAC values have to be interpreted with care (Table 2). Thus we used BIS monitoring to have some information on anesthesia depth in both groups. The mean BIS values were comparable and within the recommended range. The significantly higher applied mean inspired oxygen concentrations in the Iso group were not related to any anaesthesiological concern, but rather an accidental effect. One explanation could be the technical circumstances of the ventilator, which provides a different anesthesia handling for gaseous Xe than for the volatile anesthetic Iso.

Serious adverse events did not occur in our study. Interestingly, we could not confirm a significantly higher incidence of the diagnosis AKI according to the AKIN classification in the Iso group, even not in the ITT analysis. This might be rooted in the combined use of creatinine/ cystatin for GFR evaluation in our study, contrary to the use of creatinine/ oliguria for AKI diagnosis [30]. Creatinine has been shown to be less sensitive in the detection of small declines of renal function than cystatin [28,29,41]. Furthermore, our power calculation was performed for detection of maximum postoperative GFR decrease and not the incidence of AKI. In addition, the early termination of our study has led to a drop in the number of subjects which otherwise would have completed the study. Therefore, the higher incidence of postoperative anemia in the Iso group has to be seen within its limits, as it was only significant in the PP analysis and abolished in the ITT analysis. To our opinion, the significantly higher baseline serum urea concentration in the Xe group was most likely an incidental finding, which did not influence our results. Serum urea is known to lack sensitivity and specificity as a marker of renal function [42,43].

NGAL was identified as an early biomarker for AKI, appearing even before clinically apparent functional changes [44]. It is released from renal proximal tubular cells within 2 hours of kidney injury and can easily be detected in serum and urine [44]. We observed increased NGAL levels in all patients post-surgery in serum and particularly in urine samples, but only found a tendency to more increased urine NGAL levels in the Iso group compared to Xe. Since NGAL was not the primary outcome parameter these results are merely descriptive.

Longer-term follow-up of kidney function after 3–6 months was successfully obtained for only 28 patients. As pre-specified in our study protocol it was not feasible to perform follow-up in all patients, which came from all over Germany and even abroad. According to the German law, these patients would need a separate insurance for follow-up investigations out of the clinical routine, which would go beyond the scope of this explorative study. Therefore, this analysis was left to the discretion of the patients`family physicians and their routinely post-surgery care policy, which explains the high loss to follow-up and the different type of GFR estimation. We did not find a significant difference in the mean GFR between the two groups. One explanation could be the lower sensitivity of the creatinine-based GFR estimation method [28], beside the high loss to follow-up. Further data are required to confirm or refute our results, as our study lacked power for this longer-term analysis. If Xe does not preserve the potential short-term benefit, the clinical consequences of Xe anesthesia may be questioned, especially regarding the high costs of Xe anesthesia [7,45]. Of note, only patients with normal preoperative GFR have been included in our study. It was shown that patients with CKD and preoperatively impaired renal function are at risk for postoperative worsening of CKD and increased CKD-associated mortality [4]. It may be speculated but remains to be proven if Xe would have a stronger nephroprotective effect in these patients or in a larger study population, which would improve the cost-benefit ratio for Xe.

Clinical nephroprotection by an anesthetic like Xe might not only have an impact after renal surgery but also after further types of major surgery, like aortic repair. Postoperative AKI occurs in 40% of these patients, and mortality is even increased with the need of renal replacement [21]. Furthermore, renal IRI frequently compromises renal graft function in transplantation [21]. Preclinical data show promising results with regard to the influence of Xe on renal grafts [24–26]. Up to now there are no clinical RCTs including patients with impaired renal function [7].

The following limitations have to be acknowledged: This study was performed in a single hospital and the generalizability of our findings to other settings and populations remains unknown.

Though not significant, due to the high standard deviation the resection volume and weight was higher in the Iso than in the Xe group, despite similar tumor sizes. The reason remains unclear and the influence on our results is questionable. But these variations reflect the clinical routine of surgeries. Concurrently, hilar clamping was performed in more Xe patients and the mean hilar clamping time was longer in the Xe group. Nevertheless, this did not affect the effect of Xe on primary outcome. Of note, the low rate of hilar clamping and the short clamping times in our center may be different to other hospitals. Probably a nephroprotective effect of Xe could be enhanced after surgery with longer and more frequent hilar clamping, and the longer-term GFR would probably be different from baseline. In addition, exclusion of patients with disturbed renal function in this explorative study has probably hindered a clinically significant outcome improvement, which could justify the high costs of Xe anesthesia [45].

## Conclusions

Xe anesthesia in PN surgery showed no difference of early postoperative GFR decrease compared to Iso anesthesia. But, even if we could not show a significant result of nephroprotection by Xe, the first hints of potential nephroprotection in patients with PN should not be disregarded. Further RCTs are required to prove xenon´s possible nephroprotection in a larger heterogeneous population, especially in patients with preoperatively impaired renal function.

## Supporting information

S1 Text Methods (extended version). (DOC)

S1 Appendix Original German study protocol. (PDF)

S2 Appendix English translation of the German study protocol. (DOCX)

S1 Table CONSORT checklist. (DOC)

S2 Table Pre-existent chronic disease. (DOCX)

S3 Table Postoperative renal function. (DOCX)

S4 Table Key intervention data. (DOCX)

S5 Table Histological and pathological analyses. (DOCX)

S6 Table Adverse events. (DOCX)

S7 Table Hospital length of stay and longer-term kidney function. (DOCX)

S1 Fig Trial procedure. (TIF)
