# Supplementary material for: Renal function following xenon anesthesia for partial nephrectomy—An explorative analysis of a randomized controlled study
Source: PLoS One. 2017 Jul 18;12(7):e0181022. doi: 10.1371/journal.pone.0181022 (PMC5515428; doi:10.1371/journal.pone.0181022)
Supplement: S1 Text — (DOC) [file pone.0181022.s001.doc]

**S1 Text. Methods (extended version)**

**Study design**

The study was performed as a prospective, single-center, single-blinded, two-arm parallel-group, interventional randomized controlled, phase III pilot-study between July 2013 and October 2015. It was conducted in the University Hospital of Aachen, Germany. An ethical approval with the number EK 012/13 was received on 15^th^ April 2013 by the Ethics Committee of the University of RWTH Aachen, Aachen, Germany (Chairperson Prof. G. Schmalzing). The study design and performance was furthermore approved by the German FDA equivalent official (BfArM). The study was registered on ClinicalTrials.gov (NCT01839084) in April 2013 and conducted in adherence to the Declaration of Helsinki.

**Inclusion/ exclusion criteria**

The patients were enrolled consecutively after detailed verbal and written information by an investigator and obtainment of a written informed consent. We included all adult patients (≥18 years of age), with a suspected renal carcinoma limited to one kidney, which were planned for a partial nephrectomy (PN). The following patients were excluded from the study:

1. Patients with a chronic kidney disease (CKD), and a pre-operative glomerular filtration rate (GFR) < 60 ml min^-1^ 1.73^-1^ m^-2^
2. American Society of Anesthesiologists (ASA) status > III
3. Known contraindication and hypersensitivity to propofol, sufentanil, Xe, Iso or rocuronium
4. Severe pre-existing cardiac disease (New York Heart Association-Classification (NYHA)<III), acute coronary syndrome during the past 24 hours, hemodynamic instability or requirement of catecholamines
5. Severe respiratory disease (Forced expiratory volume in one second/ forced vital capacity (FeV1/FVC)<70% and FeV1<30%), severe respiratory insufficiency with a partial pressure of oxygen in arterial blood (PaO_2_)<60mmHg, or home oxygen therapy
6. Severe neurological disease
7. Increased intracranial pressure
8. Risk for malignant hyperthermia
9. Pregnant or breast-feeding woman
10. Legally incompetent patients
11. Refusal of consent
12. Concurrent participation in other interventional studies in the last 30 days
13. Language and communication difficulties during study information
14. People who are institutionalized by court or administrative order
15. Subjects with dependent/ employment relationship to the sponsor or the investigator

**Randomization and blinding**

The randomization sequence was generated computer-based by an independent biometrician (Institute of Medical Statistics, Informatics and Epidemiology, University Hospital of Cologne, Cologne, Germany). The patients were randomly allocated with an equal 1:1 ratio and a blocking size of 4 into the two groups Iso and Xe. Only the biometrician and the sponsor Clinical Trial Centre Aachen (CTC-A) had access to the randomization sequence, which was unmasked after the termination of data analysis. Allocation concealment was achieved by sequentially numbered, sealed opaque envelopes, which were provided to the investigator by the CTC-A. Study subjects were enrolled and pseudonymized with sequential increasing numbers by an investigator after obtaining written informed consent. Right before surgery an investigator opened the pseudonym-concordant randomization envelope in the operating room, ensuring the sequential order compliance and the performance of the assigned anesthesia. Only the patient remained blinded during the whole study procedure.

**Changes to methods after trial commencement**

Originally, we planned to provide two investigators for each patient, to enable a double-blinded procedure. The first investigator was planned for the informed consent procedure, the baseline visit and the postoperative visits in a blinded manner. The second investigator was planned for the intraoperative anesthesia conduction, which could not be blinded due to safety reasons. After inclusion of the first six patients it appeared unfeasible to continue this double-blinding procedure in the clinical routine. After intensive discussion and in agreement with the sponsor and the Ethics Committee, we decided to continue the study in a single-blinded manner. This decision was encouraged by the objectiveness of our primary outcome measure, which could not be influenced by the investigators. The patients remained blinded throughout the study.

**Intervention**

The patients were scheduled to undergo PN for renal carcinoma according to the clinical routine. After application of a standard anesthesia monitoring and a thoracic epidural catheter for postoperative pain treatment, anesthesia was induced with propofol, sufentanil and rocuronium. This was followed by intubation and ventilation with the closed-circuit respirator Felix Dual (Felix Dual™, Air Liquide Medical Systems, France). The attending anesthesiologist applied thereafter the allocated anesthetic agent for anesthesia maintenance according to the result of the randomization envelope. Patients in the Xe group were aimed to receive 60% inspired Xe with 40% oxygen and 1.2% end expiratory Iso with 40% oxygen in the Iso group. The actual maintenance dosage of the inhalational anesthetics and all other intraoperative drugs was left to the discretion of the attending anesthesiologist, according to the patients’ needs. Two experienced surgeons (AH and DP) performed open PN surgery in accordance with the standard operating procedures of the urology department.

**Primary endpoint**

The primary endpoint of this study was to determine the maximum GFR decrease between the preoperative value and the lowest value within the first seven postoperative days after PN. GFR was calculated by a combined formula incorporating serum creatinine and serum cystatin C [27,28].

**Secondary endpoints**

- Assessment of patients` vital functions (e.g. blood pressure)
- Anesthesia-related data (anesthesia duration, anesthetic concentrations, requirement of opioids, inspiratory oxygen concentration, anesthesia depth, hemodynamic and respiratory variables, applied drug and fluid dosages, and urine output)
- Routine central laboratory data
- Surgery related data (duration of tumor resection time, which defines the ischemia time, arterial clamping time, surgery duration, and blood loss)
- Duration of the anesthetic exposure before and after the tumor resection time
- Tumor-size and -histology
- Determination of the early postoperative renal function by the perioperative time course of GFR, cystatin C and creatinine
- Assessment of AKI according to the AKIN-classification [29]
- Occurrence of adverse events (AE)
- Optional determination of the later postoperative renal function (3-6 months)

**Additional endpoints**

- Further serum and urine analyses e.g. for the detection of the novel biomarker neutrophil gelatinase-associated lipocalin (NGAL) [40]

**Baseline and outcome measures**

All patients underwent a maximum of 11 visits.

**Visit 0: Baseline visit**

After written informed consent, the patient demographic and medical history data, vital and central laboratory data (including cystatin C) and a serum and urine sample for additional analyses were obtained.

**Visit 1: Surgery day**

During the intraoperative visit, we collected surgery- and anesthesia-related data. The ischemia time was defined as the tumor resection time until the termination of the tumor ground treatment. An additional arterial clamping was assessed in addition. A second serum sample for additional analyses was collected five minutes after the termination of the tumor ground-treatment. The third serum and second urine sample were collected in the Post Anesthesia Care Unit (PACU) after the surgery.

**Visit 2: Postoperative on the surgery day**

The occurrence of adverse events including AKI was assessed.

**Visit 3-9: Postoperative day (POD) 1-7**

Daily collection of patient`s vital data, adverse events, and the occurrence of AKI until POD 2. Central laboratory data including cystatin C were obtained daily until POD 7 or earlier, if the patient was discharged before. A fourth serum and third urine sample for the additional analyses was drawn on the POD 1, and a further urine sample on the POD 2. The fifth urine sample was collected on the POD 7 or at discharge, if earlier.

**Visit 10: Follow up 3-6 months (optional)**

An obligatory follow-up after discharge from hospital was not feasible and planned in our study protocol. According to the German law patients would need a separate insurance for follow-up investigations out of the clinical routine, which would go beyond the scope of this pilot-study. We only planned to contact the attending primary care physicians of the patients from all over Germany and even abroad, to obtain serum-creatinine and serum urea data, if they were collected during the clinical routine, after the surgery.

**Sample preparation**

Samples were left to clot (blood)/ sediment (urine) for 30 min at room temperature and then centrifuged at 3000 rpm at 4°Celsius (Varifuge 3.0RS, Heraeus, Osterode, Germany, and the supernatant was aliquoted and frozen at -80°Celsius until further analysis.

**NGAL- Enzyme-linked Immunosorbent Assay (ELISA)**

Neutrophil gelatinase-associated lipocalin (NGAL) in serum and urine samples was assessed with the Lipocalin-2 (NGAL) Human ELISA Kit (Abcam, Cambridge, UK) according to the manufacturers’ instructions. Samples were diluted 100-fold in 1x assay diluent before use. Colorimetric evaluation of the assay results was performed with a spectrophotometer (Sunrise™; Tecan, Crailsheim, Germany) at 450 nm.

**Study sample size calculation**

Due to the innovative character of this pilot study, we could only estimate the sample size with literature results [5] and our clinical experience. We assumed up to 30% decrease of the GFR (corresponding to 54 ml min^-1^ 1.73^-1^ m^-2^) in a control group patient with normal preoperative renal function (180 ml min^-1^ 1.73^-1^ m^-2^). A 10% reduction of the postoperative GFR decrease in the Xe group was considered as clinically significant. This would correspond to a GFR decrease of 36 ml min^-1^ 1.73^-1^ m^-2^. Assuming a large standardized effect of 0.85, the t-test requires 23 patients per group to reach 80% power at two-sided significance level 5%. To account for 10% attrition we decided to include 25 patients per group.

**Interim analyses**

Interim analyses were not planned and not performed.

**Statistical methods**

The primary endpoint - the maximum decrease of the GFR in the first seven days following PN - was evaluated by analysis of covariance adjusted for the baseline value. Secondary endpoints were evaluated with Fisher's exact test (qualitative data) or Mann-Whitney U-test (quantitative data). GraphPad PRISM® (GraphPad Software Inc., La Jolla, California, USA) was used to create figures. All data were analyzed on intention to treat (ITT) (including all randomized patients) and on per protocol (PP) basis. Patients were excluded from the PP analysis, which have received an unexpected total nephrectomy intraoperatively. Statistical calculations were done with the software SPSS Statistics (version 23; IBM Corp., Armonk, NY, USA).
