# Supplementary material for: Renal function following xenon anesthesia for partial nephrectomy—An explorative analysis of a randomized controlled study
Source: PLoS One. 2017 Jul 18;12(7):e0181022. doi: 10.1371/journal.pone.0181022 (PMC5515428; doi:10.1371/journal.pone.0181022)
Supplement: S1 Appendix — (PDF) [file pone.0181022.s002.pdf]

PRÜFPLAN – CLINICAL STUDY PROTOCOL

EINFLUSS EINER XENONANÄSTHESIE AUF DIE NIERENFUNKTION VON PATIENTEN NACH EINER NIERENTEILRESEKTION

PANEX = **PA**RTIELLE **NE**PHREKTOMIE UNTER **X**ENON

Version 02, 20. März 2013

Sponsor RWTH Aachen vertreten durch das CTC-A

Leiter der Klinischen Prüfung (LKP gem. § 40 AMG) Dr. med. Astrid Fahlenkamp
Assistenzärztin, Klinik für Anästhesiologie, RWTH Universitätsklinikum Aachen
Direktor der Klinik: Univ.-Prof. Dr.med. Rolf Rossaint

Projektmanagement Clinical Trial Center Aachen (CTC-A)
RWTH Aachen

ORGANISATIONSSTRUKTUR

SPONSOR

RWTH Aachen
vertreten durch den Rektor
dieser vertreten durch das Clinical Trials Center Aachen
Dipl.-Biol. Verena Deserno
Koordinierende Geschäftsführerin
Pauwelsstraße 30
52074 Aachen
Tel: +49 241 80 80092
Fax: +49 241 80 33 35849
E-Mail: vdeserno@ukaachen.de

ZENTRALE ORGANISATION BEIM SPONSOR

Dipl.-Biol. Angela Sudhoff
Clinical Trials Center Aachen
Pauwelsstraße 30
52074 Aachen
Tel.: +49 241 80 37429
Fax: +49 241 80 33 37429
Email: asudhoff@ukaachen.de

ETHIK-KOMMISSION

Ethik-Kommission an der Medizinischen Fakultät
der RWTH Aachen
Pauwelsstraße 30
52074 Aachen
Tel: 0241 80 89963
Fax: 0241 80 82012
E-Mail: ekaachen@ukaachen.de

ZUSTÄNDIGE BUNDESOBERBEHÖRDE

Bundesinstitut für Arzneimittel und Medizinprodukte
Kurt-Georg-Kiesinger-Allee 3
53175 Bonn

PRÜFER (MONOZENTRISCH)

Dr. med. Astrid Fahlenkamp
Assistenzärztin, Klinik für Anästhesiologie, RWTH Universitätsklinikum Aachen
Direktor der Klinik: Univ.-Prof. Dr.med. Rolf Rossaint
Pauwelsstraße 30
52074 Aachen
Tel: 0241 80 36986
Fax: 0241 80 82406
E-Mail: afahlenkamp@ukaachen.de

STELLVERTRETER

Prof. Dr.med. Mark Coburn
Oberarzt, Klinik für Anästhesiologie, RWTH Universitätsklinikum Aachen
Direktor der Klinik: Univ.-Prof. Dr.med. Rolf Rossaint
Pauwelsstraße 30
52074 Aachen
Tel: 0241 80 35394
Fax: 0241 80 82406
E-Mail: mcoburn@ukaachen.de

BETEILIGTE WISSENSCHAFTLER

Dr. med. Ana Stevanovic
Fachärztin, Klinik für Anästhesiologie, RWTH Universitätsklinikum Aachen
Direktor der Klinik: Univ.-Prof. Dr.med. Rolf Rossaint
Pauwelsstraße 30
52074 Aachen
Tel: 0241 80 88179
Fax: 0241 80 82406
E-Mail: astevanovic@ukaachen.de

BIOMETRIE / STATISTIK

Univ.-Prof. Dr. rer. nat. Walter Lehmacher
Institut für Medizinische Statistik, Informatik und Epidemiologie (IMSIE)
der Universität zu Köln
50924 Köln
Tel. +49(0)221 478-6501
Fax +49(0)221 478-6520
walter.lehmacher@uni-koeln.de

Inhaltsverzeichnis:

Organisationsstruktur

Prüfplan-Synopse

Abkürzungsverzeichnis

1 Rationale und Fragestellung

1.1 Hintergrund

1.2 Rationale

1.3 Arbeitshypothese

2 Studienziele

2.1 Primäres Ziel

2.2 Primärer Endpunkt

2.3 Sekundäre Ziele / sekundäre Endpunkte

3 Studienbeschreibung

3.1 Studiendesign

3.2 Randomisierung

3.3 Aufbewahrung der Randomisierungs-Codes und Entblindung

3.3.1 Vorzeitige Entblindung

3.3.2 Reguläre Entblindung

3.4 Personelle und technische Anforderungen

3.4.1 Wissenschaftlerinnen und Wissenschaftler, mit denen für dieses Vorhaben eine konkrete Vereinbarung zur Zusammenarbeit besteht

3.4.2 Apparative Ausstattung

4 Studienpopulation

4.1 Studien- und Interventionsdauer

4.2 Einschlusskriterien

4.3 Ausschlusskriterien

4.4 Patientenausschlusskriterien und Prozeduren

4.4.1 Gründe für einen vorzeitigen Studienausschluss und Abbruch der Prüfproduktapplikation

4.4.2 Zeitpunkt des Patientenausschlusses aus der Studie / dem Einfluss des Studienmedikamentes
4.4.3 Dokumentation des Zeitpunktes und der Art des Patientenausschlusses
4.4.4 Ersatz von ausgeschlossenen Patienten
4.4.5 Weiterverfolgung von ausgeschlossenen Patienten

5 Individueller Studienablauf
5.1 Prüfung der Ein- und Ausschlusskriterien
5.2 Patientenaufklärung
5.3 Visiten
5.3.1 Visite 0 (Baselinemessung)
5.3.2 Visite 1 (Operationstag)
5.3.3 Visite 2 (OP-Tag, postoperativ)
5.3.4 Visite 3 (1. postoperativer Tag)
5.3.5 Visite 4-8 (2.-6. postoperativer Tag)
5.3.6 Visite 9 (7. postoperativer Tag bzw. am Entlasstag / Studienende)
5.3.6 Visite 10 (nach 3-6 Monaten)

6 Prüfmedikation
6.1 Bezeichnung
6.2 Verabreichung der Prüfmedikation
6.3 Verpackung, Bezeichnung und Aufbewahrung der Prüfmedikation
6.4 Verantwortung des Umgangs mit der Prüfmedikation
6.5 Kriterien für einen vorzeitigen Studienabbruch aufgrund der eingesetzten Prüfmedikation
6.6 Bezeichnung der restlichen Studienmedikamente
6.7 Verantwortung über die restliche Studienmedikation
6.8 Begleittherapie
6.9 Unerlaubte Medikation

7 Beurteilung der Wirksamkeit
7.1 Bezeichnung der Wirksamkeitsparameter
7.1.1 Auswahlkriterien demographische Daten
7.1.2 Primäre Wirksamkeitskriterien
7.1.3 Sekundäre Wirksamkeitskriterien

7.2 Messung, Registrierung und Analyse der Wirksamkeitskriterien

8 Sicherheitsanalysen

8.1 Sicherheitsmassnahmen während der Allgemeinanästhesie zur Nierenteilresektion (Visite 1)

8.1.1 Verabreichung der Prüfmedikation

8.1.2 Klinische Sicherheitsparameter

8.2 Sicherheitsmassnahmen während der Visiten 0 und 2-9

8.2.1 Klinische Sicherheitsparameter und Untersuchungen

8.2.2 Blutentnahmen und Laborparameter

8.3 Bewertung, Aufzeichnung und Analyse der Sicherheitsparameter

8.4 Umgang mit unerwünschten Ereignissen (AE)

8.5 Umgang mit schwerwiegenden unerwünschten Ereignissen (SAE)

8.6 Umgang mit suspekten unerwarteten schwerwiegenden Nebenwirkungen (SUSAR)

8.7 Erneute Überprüfung der Nutzen-Risiko-Bewertung

9 Statistik

9.1 Fallzahlplanung

9.2 Patientenauswahl für den Einschluss in die Analyse

9.3 Anwendung von statistischen Tests

9.4 Prozeduren zur Behandlung von fehlenden Daten

9.5 Auswertungsprozeduren bei Abweichungen vom ursprünglichem statistischen Plan

10 Dateneingabe und Datenmanagement

10.1 Dateneingabe

10.2 Datenaufbewahrung

10.3 Datenmanagement

10.3.1 Zugang zu Quelldaten

10.3.2 Monitoring

10.4 Abschlussbericht

11 Qualitätskontrolle und Qualitätssicherung
11.1 Qualitätskontrolle
11.2 Qualitätssicherung

12 Ethische und Administrative Aspekte
12.1 Erläuterungen zu den vorgesehenen Untersuchungen bei Versuchen an Menschen oder an vom Menschen entnommenen Material oder Tieren
12.2 Zustimmung einer unabhängigen Ethik-Kommission
12.3 Zustimmung der Bundesbehörde
12.4 Änderung des Studienprotokolls
12.5 Studienabschluss
12.6 Abschlussbericht

13 Dokumentation
13.1 Datenerhebung und Datenverwaltung
13.1.1 Quelldaten (Source Data)
13.1.2 Case Report Forms (CRF`s)
13.2 Digitalisierung der Studiendaten
13.3 Archivierung der Studiendaten

14 Formales
14.1 Finanzierung
14.2 Studienversicherung

15 Publikationsvereinbarung

16 Signaturen

17 Literaturverzeichnis

I Prüfplan-Synopse

**Titel der Studie**	Einfluss einer Xenonanästhesie auf die Nierenfunktion von Patienten nach einer Nierenteilresektion
**Kurzbezeichnung**	**PaNeX** = **Pa**rtielle **Ne**phrektomie unter **X**enon
**EudraCT-Nummer**	2012-005698-30
**Studiennummer**	12-051
**Clinical Trials Nummer**	NCT01839084
**Hauptprüfer**	Dr. med. Astrid Fahlenkamp
**Stellvertreter**	Prof. Dr. med. Mark Coburn
**Studienzentrum**	Universitätsklinikum der RWTH Aachen<br>Klinik für Anästhesiologie<br>Pauwelsstraße 30<br>52074 Aachen
**Studienzeitplan**	Studienbezogen:<br>Beginn des Patienteneinschlusses: Juni 2013<br>Ende des Patienteneinschlusses: Oktober 2014<br>Ende der Auswertungsphase März 2015<br><br>Patientenbezogen:<br>Die Studiendauer mit täglichen innerklinischen Untersuchungen läuft für jeden Patienten vom Moment der schriftlichen Einwilligung bis zum Entlassungstag, maximal jedoch bis zum siebten postoperativen Tag. Abhängig vom Zeitpunkt des Eingriffs entspricht das neun bis zehn Tage. Die Interventionsdauer entspricht dabei der für den Eingriff notwendigen Anästhesiedauer, welche üblicherweise drei Stunden beträgt. 3-6 Monate postoperativ erfolgt eine weitere Datenerhebung mit Hilfe des behandelnden Urologen /Hausarztes.
**Studiendesign**	Monozentrische, prospektive, randomisierte, kontrollierte, doppelblinde (Patienten und Auswerter-verblindete) Pilotstudie

**Phase der Studie**	Phase III
**Primäres Ziel**	Prüfung des Einflusses einer Xenonanästhesie im Vergleich zu einer Isoflurannarkose, als Referenzsubstanz mit nephroprotektiven Eigenschaften, auf die Verminderung der GFR (Glomerulären Filtrationsrate) bei Patienten, die sich einer Nierenteilresektion unterziehen.
**Primärer Endpunkt**	Maximaler Abfall der GFR (Absolutwert in ml/min/1,73 m² KOF) gemessen als Differenz zwischen dem präoperativen Ausgangswert und dem niedrigsten Wert innerhalb der ersten sieben Tage nach Nierenteilresektion.
**Sekundäre Endpunkte**	• Demographische Daten: Alter, Größe, BMI<br>• Anästhesiedaten: Anästhetika-Konzentrationen, Opiatverbrauch und die insp. Sauerstoffkonzentration während der Narkose<br>• Direkte Tumorresektions- und Manipulations- bzw.Ischämiezeit<br>• Intraoperativer Blutverlust<br>• Dauer der Anästhetika-Exposition vor und nach der direkten Tumorresektionszeit und somit der Ischämiezeit<br>• Tumorgröße und –histologie; ggf. Nachweis von HIF-1α im gesunden Resektionsrandgewebe<br>• Auftreten von macrophage migration-inhibiting factor (MIF) im Serum<br>• Verlauf der GFR, bestimmt mit dem Cystatin C-Wert im Serum, bis zum Entlasstag, längstens 7 Tage postoperativ<br>• Verlauf des Serum-Kreatinins bis zum Entlasstag, längstens 7 Tage lang postoperativ<br>• Urinausscheidung während des Eingriffes und innerhalb der ersten 48 h postoperativ<br>• Ggf. Bestimmung von KIM-1 oder NGAL aus Urinproben vor und nach Resektion<br>• Auftreten eines akuten Nierenversagens (acute kidney injury) nach der AKIN-Klassifikation

	• Auftreten von AE`s und SAE`s<br>• Erfassung der Nierenfunktion nach 3-6 Monaten durch Bestimmung des Serum-Kreatinins und der GFR über den Cyststin C Wert.
**Patientenzahl**	n=50 insgesamt, d.h. n=25 mit einer Xenonanästhesie und n=25 mit einer Isoflurananästhesie
**Einschlusskriterien**	• unterschriebene Einwilligungserklärung<br>• Patienten mit einem Nierenkarzinom mit Beschränkung auf eine Niere)<br>• geplante OP: Nierenteilresektion<br>• Männliche oder weibliche Patienten, ≥ 18 Jahre<br>• Personen, die geschäftsfähig und mental in der Lage sind, die Anweisungen des Studienpersonals zu verstehen und ihnen Folge zu leisten.
**Ausschlusskriterien**	• chronische Niereninsuffizienz mit einer präoperativen GFR < 60 ml/min/1,73 m² KOF<br>• American Society of Anesthesiologists (ASA) Status > III<br>• Bekannte Kontraindikationen und Allergien gegen Propofol, Sufentanil, Xenon, Isoflurane, oder Rocuronium<br>• Schwere kardiale Vorerkrankungen (NYHA > III), akutes Koronarsyndrom innerhalb der letzten 24h, hämodynamische Instabilität, Bedarf einer inotropen Unterstützung<br>• schwere Lungen- und Atemwegserkrankungen (FeV1/FVC < 70% und FeV1 < 30% des Sollwertes), oder schwere respiratorische Insuffizienz mit einem PaO2 von <60mmHg, oder Heimsauerstofftherapie, COPD GOLD IV<br>• Patienten mit schweren neurologischen Vorerkrankungen<br>• Patienten mit erhöhtem intrakraniellen Druck<br>• Patienten mit einer Veranlagung zur malignen Hyperthermie<br>• Frauen während Schwangerschaft / Stillzeit<br>• Nicht-Einwilligungsfähigkeit<br>• Ablehnung der Studie durch den Patienten<br>• Teilnahme an weiteren Studien innerhalb der letzten 30 Tage

	• Sprach-/ Verständigungsprobleme im Rahmen der Aufklärung<br>• Personen, die aufgrund gerichtlicher oder behördlicher Anordnung in einer Anstalt untergebracht sind.<br>• Personen, die in einem Abhängigkeits-/Arbeitsverhältnis zum Sponsor oder Prüfer stehen.
**Studienplan und Durchführung**	Nach der Auswahlvisite, Aufklärung und Einwilligung durch den Patienten wird zunächst die Baselineuntersuchung (Visite 0) durch einen Studienarzt durchgeführt und der Einschluss hiermit vervollständigt. Demographische Daten, wie Alter, Größe, BMI, Geschlecht, ASA Status etc. werden in der Baselineuntersuchung dokumentiert. Vitalparameter (Herzfrequenz, Blutdruck, Sauerstoffsättigung unter Raumluft), der Laborparameter (kleines Blutbild, Gerinnungsparameter, Serum-Harnstoff, Transaminasen, Creatinkinase, Serum-Kreatinin und die präoperative GFR) und das Auftreten von macrophage migration-inhibiting factor (MIF) im Serum). Eine Urinprobe wird asserviert.<br>Anschließend erhalten die eingeschlossenen Patienten eine Studiennummer.<br>Es folgt die operative Nierenteilresektion, entsprechend den Standards der Klinik für Urologie des UKA, die Narkose hierzu wird durch einen Studienarzt durchgeführt (Visite 1). Zur postoperativen Schmerztherapie erhält jeder Patient präoperativ einen thorakalen PDK (Periduralkatheter).<br><u>Narkoseeinleitung:</u><br>• Propofol 1,5 – 2mg/kgKG durch Bolustitration<br>• Sufentanil 0,2 – 0,3µg/kgKG durch Bolustitration<br>• Rocuronium 0,6 - 0,9mg/kgKG<br>• Nach Intubation wird der Cuffdruck des Tubus auf 25-30cmH_2O eingestellt und während der Anästhesie auf diesem Niveau gehalten, um eine ausreichende Dichtigkeit des Tubus zu gewähren und Trachealschäden zu vermeiden.<br><u>Narkoseaufrechterhaltung:</u><br>• Xenon-Gruppe: 60% Xenon/ 40% O_2<br>• Kontroll-Gruppe: 1,2 Vol.% Isoflurane/ 40% O_2/Luftgemisch

- Sufentanil titriert nach klinischem Bedarf
- Gabe von 4mg Ondansetron zur Prophylaxe von Übelkeit und Erbrechen

Monitoring:

- entsprechend den SOP`s unserer Klinik für Anästhesiologie; alle 5 Min. Dokumentation von: (nicht-) invasiven Blutdruckwerten (RR_{sys}, RR_{dias}, RR_{MD}) und der HF
- kontinuierliche EKG-Ableitung und Pulsoximetrie
- Alle 15 Min. Erfassung der Beatmungsparameter (Fi O_2, Sättigung, Fe O_2, CO_{2et}, AMV, AF, Beatmungsdrücke)
- Anästhesiedaten: Anästhetika-Konzentrationen, Opiatverbrauch und die insp. Sauerstoffkonzentration werden alle 15 Min. erfasst
- kontinuierliche Temperaturmessung
- Stündliche Urinausscheidung und Uringesamtmenge am Ende der Operation
- Dokumentation der Schnitt-Nahtzeit, der direkten operativen Manipulationszeit (Tumorresektion)und/oder Ischämiezeit an der Niere, des intraoperativen Blutverlustes und der Anästhetikaexpositionsdauer bis zum Beginn der direkten Tumorresektion und nach Ende dieser bis zum Narkoseende.
- Ca. 5 Min. nach der abschliessenden Versorgung des Tumorgrundes: Blutentnahme zur Messung des macrophage migration-inhibiting factor (MIF) im Serum
- Dokumentation des Tumorpräparatergebnisses des Instituts für Pathologie (Größe, Histologie)
- Ggf. HIF-1α Bestimmung im tumorfreien Rand des Präparats
- Im AWR werden die invasiven Blutdruckwerten (RR_{sys}, RR_{dias}, RR_{MD}) und die HF alle 15 Min. erfasst und das EKG und die Pulsoxymetrie werden kontinuierlich abgeleitet.
- Bei Aufnahme in den AWR erfolgt eine weitere Blutentnahme zur Messung des macrophage migration-inhibiting factor (MIF) im Serum

	<u>Folgevisiten: werden durch einen Studienarzt durchgeführt</u><br><u>Visiten 2-8 (1.- 6. postoperativer Tag):</u><br>• Urinausscheidung alle 6 h innerhalb der ersten 24h postoperativ. Am 2. postoperativen Tag die Urin-gesamtausscheidung des 2. postoperativen Tages.<br>• Morgendliche Blutentnahme und Messung der Vital-parameter (Blutdruck, Herzfrequenz, Sauerstoffsättigung).<br>• Zu bestimmende Laborwerte: Cystatin C, woraus die GFR berechnet wird, Kreatinin im Serum, sowie die Sicherheitsparameter (kleines Blutbild, Gerinnungsparameter, Serum-Harnstoff, Transaminasen, Creatinkinase). Am ersten postoperativen Tag wird eine weitere Blutprobe entnommen, um die Konzentration von macrophage migration-inhibiting factor (MIF) im Serum zu bestimmen.<br>• Asservation von zwei Urinproben an den Tagen 1 und 2<br>• Erfassung eines akuten Nierenversagens, anhand der AKIN-Klassifikation.<br>• Erfassung und Dokumentation sämtlicher AE`s und SAE`s<br><u>Visite 9 (7. postoperativer Tag/bzw. Entlasstag):</u><br>• Studienabschlussuntersuchung (Vitalparameter, klinische Untersuchung und Labor, Urinprobe)<br>Visite 10: (3-6 Mon. postoperativ):<br>• Bestimmung der GFR und des Serum-Kreatinins mit Hilfe des behandelnden Urologen/Hausarztes
**Bewertungskriterien**	Das primäre Kriterium maximaler Abfall der GFR wird anhand der täglichen Bestimmung der GFR vom präoperativen Wert bis zum niedrigsten Wert innerhalb der ersten 7 Tage nach Nierenteilresektion bewertet.<br>Die sekundären Kriterien:<br>• Demographische Daten: Alter, Größe, BMI, Geschlecht, ASA Status<br>• Anästhesiedaten: Anästhetika-Konzentrationen, Opiatverbrauch und die insp. Sauerstoffkonzentration<br>• Dauer der direkten Tumorresektionszeit und der Ischämiezeit

	• Intraoperativer Blutverlust<br>• Dauer der Anästhetika-Exposition vor und nach der direkten Tumorresektionszeit<br>• Tumorgröße und –Histologie, ggf. Aktivierung von HIF-1α im gesunden Resektionsrandgewebe<br>• Auftreten von macrophage migration-inhibiting factor (MIF) im Serum<br>• Verlauf der GFR, durch Bestimmung des Cystatin C-Wertes im Serum<br>• Verlauf des Serum-Kreatinins<br>• Ggf. Auftreten von KIM (kidney injury molecule)-1 oder NGAL (neutrophil gelatinase-associated lipocalin) im Urin<br>• Urinausscheidung während des Eingriffs und am 1. postoperativen Tag<br>• Auftreten eines akuten Nierenversagens nach der AKIN-Klassifikation innerhalb der ersten 7 Tage postoperativ<br>• Nierenfunktion nach 3-6 Monaten<br>werden entsprechend der Beschreibung der Visiten (s.o.) erfasst.
**Statistische Rationale**	Die Hauptzielvariable maximaler Abfall der GFR wird primär mit einer Kovarianzanalyse mit dem Faktor Gruppe und der Kovariaten Ausgangswert über einen 2-seitigen Mann-Whitney-Test zum Niveau von 5% ausgewertet. Die primäre Auswertung wird nach dem Intention-to-Treat-Prinzip durchgeführt (fehlende Werte werden durch Imputationen, deren genauere Methoden in einem späteren Statistical Analysis Plan definiert werden, ersetzt, sodass konservative Abschätzungen des Effekts erreicht werden), die sekundäre nach dem per-protocol-Prinzip. Die Nebenzielvariablen werden analog mit üblichen 2-Stichproben-Tests deskriptiv bzw. explorativ ausgewertet.<br>Die deskriptive Auswertung für die Baseline- und Outcome-Variablen erfolgt mit Hilfe der üblichen Maßzahlen und Kenngrößen, Graphiken und Tests (p-Werte) in Abhängigkeit der Skalenniveaus der Merkmale.
**Nutzen-Risiko**	Die in dieser Studie eingesetzten Prüfpräparate, sind

**Bewertung**	zugelassene Medikamente, die täglich zur Durchführung einer Vollnarkose eingesetzt werden.<br>Lässt sich im Rahmen der Studie ein nephroprotektiver Effekt unter der Xenonanästhesie nachweisen, wird dies in einer multizentrischen randomisiert kontrollierten klinischen Studie mit größerer und auf den Effekt bezogener Fallzahl auf Signifikanz überprüft. Langfristig könnte sich daraus ein viel versprechender Nutzen für die Allgemeinheit ableiten, indem die Rate an Nierenfunktionseinschränkungen vermindert und/oder den Grad der Funktionseinschränkung abschwächt werden kann und damit kurz- und langfristige Folgen (Kurzzeitdialyse, chronische Niereninsuffizienz, kardiovaskuläres Risiko) für Patienten und Gesundheitssystem reduziert werden können.

II Abkürzungsverzeichnis:

**AE**	Adverse Event (Unerwünschtes Ereignis)
**AF**	Atemfrequenz
**AMV**	Atemminutenvolumen
**AMG**	Arzneimittelgesetz
**AR**	Adverse Reaction (Unerwünschte Arzneimittelwirkung)
**ASA**	American Society of Anesthesiologists
**AWR**	Aufwachraum
**BfArM**	Bundesinstitut für Arzneimittel und Medizinprodukte
**BMI**	Body-Mass-Index
**CO_{2et}**	enttidales CO_2
**CRF**	Case Report Form (Erhebungsbogen)
**EKG**	Elektrokardiogramm
**Fe O_2**	expiratorische Sauerstoff-Konzentration
**Fi O_2**	inspiratorische Sauerstoff-Konzentration
**GCP**	Good Clinical Practice
**GFR**	Glomeruläre Filtrations Rate
**HIF-1α**	Hypoxia inducible factor-1α
**HF**	Herzfrequenz
**i. v.**	intravenös
**KG**	Körpergewicht
**KIM-1**	**kidney injury molecule-1**
**KOF**	Körperoberfläche
**LKP**	Leiter der Klinischen Prüfung
**MAD**	Mittlerer Arterieller Druck
**MIF**	macrophage migration-inhibiting factor
**NGAL**	**neutrophil gelatinase-associated lipocalin**
**NYHA**	New York Heart Association
**PaO2**	arterieller Sauerstoffpartialdruck
**PID**	Patientenidentifikationsnummer (=Randomisierungsnummer)
**RR_{dias}**	diastolischer Blutdruck
**RR_{MD}**	Mitteldruck
**RR_{sys}**	systolischer Blutdruck

**SaO2**	Sauerstoffsättigung
**SAE**	Serious Adverse Event (schwerwiegend unerwünschtes Ereignis)
**SAR**	Serious Adverse Reaction (schwerwiegende Nebenwirkung)
**SOP**	Standard Operating Procedure
**SUE**	Schwerwiegend unerwünschtes Ereignis
**SUSAR**	Suspected Unexpected Seriouse Adverse Reaction (Verdachtsfall einer unerwarteten schwerwiegenden Nebenwirkung)
**UAR**	Unexpected Adverse Reaction (unerwartete Nebenwirkung)
**UE**	Unerwünschtes Ereignis

III Beteiligte Personen / Institutionen

**Leiter der klinischen Prüfung**	**Dr. med. Astrid Fahlenkamp**<br>Klinik für Anästhesiologie<br>Universitätsklinikum Aachen<br>Pauwelsstr. 30<br>52074 Aachen<br>Tel.: +49 (0) 241-8036986<br>Fax.:+49 (0) 241-8082406
**Stellvertretender Leiter der klinischen Prüfung**	**Prof. Dr. med. Mark Coburn**<br>Klinik für Anästhesiologie<br>Universitätsklinikum Aachen<br>Pauwelsstr. 30<br>52074 Aachen<br>Tel.: +49 (0) 241-8035394<br>Fax.:+49 (0) 241-8082406
**Sponsor**	RWTH Aachen, vertreten durch den Rektor<br>dieser vertreten durch das<br>Clinical Trials Center Aachen (CTC-A)<br>Koordinierende Geschäftsführerin:<br>Dipl.-Biol. Verena Deserno, M.A.

1 Rationale und Fragestellung

1.1 Hintergrund

Das Nierenzellkarzinom ist der dritthäufigste urologische Tumor in Europa und den USA, Tendenz steigend, mit einer besonderen Zunahme der zufällig (bei Bildgebungsverfahren) entdeckten kleinen Tumoren im Stadium T1 (1-4). Bei diesem Stadium ist die Nierenteilresektion das Verfahren der Wahl (5). Auch bei sorgfältig auserwählten Patienten mit einem Tumor im Stadium T2 wird die Nierenteilresektion empfohlen (5). Obwohl bei dieser OP-Technik ein erheblicher Teil des Nierengewebes gegenüber der radikalen Nephrektomie erhalten bleibt, sind größere perioperative Komplikationen durch die operativ bedingte Ischämie im Rahmen der Manipulation durch die Tumorresektion und eine eventuelle Nierenklemmzeit für die Zeit der Resektion beschrieben (6). Besonders die Beeinträchtigung der Nierenfunktion durch den Ischämie-Reperfusionsschaden mit einer post-operativen Erniedrigung der glomerulären Filtrationsrate (GFR) um 16 bis 30% ist ein klinisch relevantes Problem (6-8). Neben einer zeitlichen Limitation der Abklemm- und Manipulationszeit („so kurz wie möglich") werden nephroprotektive Manöver mit Mannitol als Osmodiuretikum und Kühlung während Ischämie bei vorhergesehener mehr als 30-minütiger Ischämiezeit durchgeführt, doch auch darunter ist eine Verminderung der GFR häufig, die durch lange Operationszeiten und einen ausgeprägten Blutverlust weiter negativ beeinflusst wird (6,8). Aus der klinischen Versorgung ist ein klassischer Verlauf der GFR nach Nierenteilresektion mit „Peak"-Abfall (8) und anschließender Erholung der GFR, allerdings auf einem oft niedrigeren Niveau, bekannt. Studiendaten zu postoperativen Veränderungen der GFR sind hierbei sehr begrenzt (9); insbesondere zum frühen postoperativen Verlauf und zum maximalen Abfall der GFR fehlen valide Studien. Ein Therapieansatz, welcher die Widerstandsfähigkeit der Niere erhöht und damit hilft, den perioperativen Ischämie/-Reperfusionsschaden zu überstehen, hat wichtige klinische Relevanz.

1.2 Rationale

Xenon als inhalatives Anästhetikum ist bereits seit 2005 in Deuschland und seit 2007 in Europa zugelassen. Es wird zur Durchführung einer balancierten Anästhesie in Kombination mit Opioiden bei Erwachsenen mit einer American Society of

Anesthesiology-Klassifikation ASA ≤ III eingesetzt. Für Xenon konnten experimentell organprotektive Effekte nachgewiesen werden. In unterschiedlichen Schädigungsmodellen wurden dabei gewebeprotektive Wirkungen an Herz und Hirn demonstriert, die sowohl ein mechanisches Trauma als auch den Reperfusionsschaden nach Ischämie umfassten (12-15). Dabei erwiesen sich prä- und postkonditionierende Therapieprotokolle als wirksam (16,17). Kürzlich konnte in einem *in vivo* Ischämie-Reperfusions-Modell und *in vitro* in einem Glucose-Sauerstoff-Deprivationsmodell an tubulären Nierenzellen gezeigt werden, dass eine Vorbehandlung mit Xenon nephroprotektiv wirkt (18,19). In beiden Arbeiten wurde eine gesteigerte Expression von hypoxia inducible factor-1α (HIF-1α) in den mit Xenon behandelten Zellen bzw. den Nieren der mit Xenon behandelten Tiere nachgewiesen (18,19). Inhibition des HIF-1α Aktivität hob den nephroprotektiven Effekt durch Präkonditionierung mit Xenon im Ischämie-Reperfusions-Modell an Ratten auf (18). Einen weiteren Hinweis auf einen nephroprotektiven Effekt von Xenon lieferte kürzlich die Arbeitsgruppe um J. Pype mit einem Nierentransplantationsmodell an Ratten: Eine Sättigung der kalten Aufbewahrungslösung für Transplantatnieren mit Xenon führte im Tierversuch dazu, dass Transplantatnieren nach sechsstündiger kalter Ischämie in diesen Lösungen weniger histologische Schäden aufwiesen (20). Die mit in Xenon-Lösung gelagerten nierentransplantierten Tiere hatten nach 7 und 14 Tagen eine bessere Kreatinin-Clearance als Maß für glomeruläre Funktion und eine geringere Albuminausscheidung im Urin als Maß für tubuläre Schädigung als Tiere, die eine in mit Luft gesättigter Aufbewahrungslösung gelagerte Niere erhalten hatten (20). Im Rahmen einer klinischen Studie unserer AG an herzchirurgischen Patienten konnte kürzlich eine geringere Rate an Nierenfunktionsstörungen, gemessen am Anstieg des post-operativen Kreatinins im Serum nach Xenon-Anästhesie gezeigt werden (21). Die gleiche AG zeigte an weiteren herzchirurgischen Patienten, dass eine Ischämie-Reperfusion zu einer erhöhten Freisetzung des macrophage migration-inhibiting factor (MIF) im Serum führt. Diese Freisetzung von MIF war mit einer erhöhten antioxidativen Kapazität und einem verminderten postoperativen Auftreten von akutem Nierenversagen verbunden (22). Der Einfluss von Xenon im Vergleich zu Isoflurane auf die Freisetzung von MIF, als eine Erklärung für den verminderten Nierenschaden nach Xenonexposition soll daher in dieser Studie mit untersucht werden.

Über die experimentell gezeigten organprotektiven Effekte hinaus stellt Xenon ein effektives Anästhetikum mit einem gegenüber den herkömmlichen Anästhetika günstigem hämodynamischen Wirkungsprofil mit hoher Blutdruckstabilität und niedrigen Herzfrequenzen dar (10,11). Bei anderen Anästhetika wie Isoflurane, Desflurane, Sevoflurane und Propofol finden sich während einer Allgemeinanästhesie häufig niedrigere Blutdruckwerte; teilweise ist zum Erhalt der Blutdruckstabilität eine gegensteuernde Therapie mit Katecholaminen erforderlich. Sowohl niedrige arterielle Mitteldrücke als auch vasokonstriktive Medikamente wie Noradrenalin wirken sich zusätzlich negativ auf die Nierenfunktion aus. Hieraus könnte sich ein weiterer deutlicher Vorteil von Xenon in der Risiko-Nutzen-Abschätzung ergeben.
Bei Xenon handelt es sich um ein sehr seltenes und natürliches Gas, so dass seine Bereitstellung mit erhöhten Kosten verbunden ist. Doch eine postoperative Niereninsuffizienz geht oftmals mit einer verlängerten Krankenhausverweildauer, mehr Kosten und einer erhöhten Mortalität einher (23). Somit könnte eine Verminderung der postoperativen Niereninsuffizienz durch Xenon, sei es durch organprotektive Effekte oder zusätzliche hämodynamische Stabilität, die die damit verbundenen erhöhten Kosten der Xenonapplikation aufwiegen.

1.3 Arbeitshypothese

In dieser Pilotstudie soll die frühe postoperative Einschränkung der GFR (glomeruläre Filtrationsrate) nach einer Nierenteilresektion unter Xenonanästhesie mit einer Standardanästhesie verglichen werden. Dazu sollen in der Kontrollgruppe die GFR täglich evaluiert und der maximale Abfall zum Ausgangswert innerhalb von 7 Tagen postoperativ bestimmt werden. Zum Abschätzen des längerfristigen Verlaufs werden die GFR und der Serum-Kreatininwert nach 3-6 Monaten erneut bestimmt. Anschließend werden diese Daten mit denen der Xenon-Gruppe verglichen. Die Arbeitshypothese postuliert, dass sich bei Operationen mit temporärer Verminderung oder gar Unterbrechung der Nierenperfusion eine Vorbehandlung mit Xenon günstig auf die postoperative Nierenfunktion erweist, was zu einer Verringerung des maximalen GFR-Abfalls führen würde. Dazu könnten sowohl die hämodynamische Stabilisierung, die eine bessere Nierenperfusion vor und nach der Manipulation im Rahmen der Nierenresektion gewährleistet, als auch ein nephroprotektiver Effekt, dessen zelluläres Ziel nur zum Teil bekannt ist, beitragen. Der durch Xenon

induzierte organprotektive Effekt wird in vielen Fällen durch Präkonditionierung erreicht, so dass auch eine Protektion des Nierengewebes noch während Ischämie angenommen werden kann. Der neue Aspekt dieser Hypothese ist, dass bei der Suche nach nephroprotektiven Optionen für Nierenchirurgie mit Ischämie noch nie die dazu obligatorische Anästhesie in unterschiedlichen Formen untersucht worden ist. Eine Anästhesie mit Xenon hat aus den oben beschriebenen Gründen gutes Potential, die Nephroprotektion während renaler Ischämie auch in klinisch relevanter Größe auszuüben. Eine kontrollierte klinische Studie hierzu fehlt aber bisher. Als Kontrolle für die Xenon-Anästhesie zur Nierenteilresektion wird Isoflurane als Anästhetikum mit der bisher höchsten nephroprotektiven Eigenschaft gewählt. Für Isoflurane konnten im Tierversuch nephroprotektive Eigenschaften aufgezeigt werden (24,25), die stärker als bei Sevoflurane oder Desflurane waren (26). Potentiell nephrotoxische Eigenschaften wie Sevoflurane hat es nicht (27). Isoflurane ist den anderen etablierten Anästhetika Sevoflurane, Desflurane und Propofol in Bezug auf die Nierenfunktion nach Nierentransplantation oder großer Chirurgie nicht unterlegen (28-30).

2 Studienziele

2.1. Primäres Ziel

Das Ziel dieses Projektvorhabens ist, in einer randomisierten kontrollierten Pilotstudie eine mögliche Nephroprotektion durch Xenonanästhesie bei Nierenteilresektionen anhand der Evaluation der frühen post-operativen Nierenfunktion gegenüber einer Standard-Anästhesie mit Isoflurane zu analysieren und die mögliche Effektgröße abzuschätzen. Zur Beurteilung der postoperativen Nierenfunktion wird hierbei die Messung der GFR herangezogen. Lässt sich ein nephroprotektiver Effekt und damit ein Benefit der Xenonanästhesie nachweisen, wird dies in einer multizentrischen randomisiert kontrollierten klinischen Studie mit größerer und auf den Effekt bezogener Fallzahl auf Signifikanz überprüft. Langfristiges Ziel dieser Arbeiten ist es eine zusätzliche Präventionsstrategie zu entwickeln, die bei bekannt nierenfunktionsschädigenden Operationen (Nierenteilresektionen, Nierentransplantationen) die Rate an Nierenfunktions-einschränkungen vermindern und/oder den Grad der Funktionseinschränkung abschwächen kann und damit kurz- und langfristige Folgen (Kurzzeitdialyse,

chronische Niereninsuffizienz, kardiovaskuläres Risiko) für Patienten und Gesundheitssystem reduziert.

2.2 Primärer Endpunkt

Der primäre Endpunkt der Studie ist der maximale Abfall der GFR (Absolutwert in ml/min/1,73 m² KOF) vom präoperativen Ausgangswert bis zum niedrigsten Wert innerhalb der ersten sieben Tage nach Nierenteilresektion. Die GFR wird als Ausgangswert vor OP und täglich in den sieben postoperativen Tagen mit Hilfe des Cystatin C-Werts im Serum bestimmt (31-33). Der primäre Endpunkt „maximaler GFR-Abfall" wird berechnet als Differenz aus der präoperativen Ausgangs-GFR minus der niedrigsten postoperativen GFR innerhalb dieser sieben postoperativen Tage.

2.3 Sekundäres Ziel /sekundäre Endpunkte

Als sekundäre Endpunkte werden bestimmt: Dauer der direkten Manipulation/ Ischämiezeit an der Niere zur Tumorresektion, Dauer der Anästhetika-Exposition vor und nach der Tumorresektionszeit, der intraoperative Blutverlust, Tumorgröße und – histologie, gegebenenfalls die Aktivierung von HIF 1 im gesunden Resektionsrandgewebe, Verlauf der GFR, bestimmt mit dem Cystatin C-Wert im Serum, und Verlauf des Serum-Kreatinins in den ersten sieben Tagen nach Nierenteilresektion, gegebenenfalls die Bestimmung von KIM-1 oder NGAL im Urin vor und nach Resektion, Urinausscheidung während des Eingriffs und an den post-operativen Tagen 1 bis 3, Auftreten eines akuten Nierenversagens (acute kidney injury) nach der AKIN-Klassifikation (34). Bereits innerhalb von 48h lässt sich eine Nierenfunktionsstörung nach der AKIN-Klassifikation bei zunehmendem Schweregrad mit einer erhöhten Mortalität assoziieren. Mehrere Studien konnten diesen Zusammenhang verifizieren. Wie in einer rezenten Metaanalyse festgestellt, verdoppelt sich das durchschnittliche Risiko zu versterben im Vergleich zu Patienten ohne AKI für Stadium I (RR= 2,4) und steigt über Stadium II zu Stadium III (RR = 4 ,2 und RR = 6,4) stetig weiter an (35).

Der macrophage migration-inhibiting factor (MIF) im Serum wird präoperativ, 5 Min. nach Ende der Versorgung des Tumorgrundes, direkt postoperativ im Aufwachraum

und nach 24h bestimmt. Zusätzlich werden im Rahmen der Pilotstudie demographische Daten wie Alter, Größe, BMI und Anästhesiedaten wie Anästhetika-Konzentrationen, Opiatverbrauch und die inspiratorische Sauerstoffkonzentration bestimmt, um die Vergleichbarkeit der Gruppen an diesen Parametern zu überprüfen. Sicherheitsdaten wie Vitalparameter (Sauerstoffsättigung des Bluts, Blutdruck, Herzfrequenz) während der Allgemeinanästhesie, allgemeine Laborparameter (kleines Blutbild, Gerinnungsparameter, Serum-Harnstoff, Transaminasen, Creatinkinase) nach der Operation und sämtliche Adverse Events (AEs) und Serious Adverse Events (SAEs) nach GCP werden ebenfalls erhoben. Nach 3-6 Monaten sollen der Serum-Kreatininwert und die GFR erneut bestimmt werden, um den Verlauf der Nierenfunktion zu bestimmen. Dies erfolgt über die Kontaktierung des behandelnden Hausarztes/Urologen im Rahmen der postoperativen Nachuntersuchungen.

3 Studienbeschreibung

3.1 Studiendesign

Die Pilotstudie wird als Phase III Studie prospektiv in einem randomisiert kontrollierten Patienten- und Auswerter-blinden Design, monozentrisch im Universitätsklinikum Aachen, durchgeführt.

- Gruppe 1: Xenon-Gruppe: 60% Xenon/ 40% O_2 inspiratorisch
- Gruppe 2: Kontroll-Gruppe: 1,2 Vol.% et. Isoflurane/ 40% O_2/Luftgemisch

Die übrigen für die standardisierte Studienanästhesie notwendigen Medikamente werden in einer Standard Operating Procedure (SOP) vor Beginn der Patientenrekrutierung festgelegt.

3.2 Randomisierung

Die Randomisierung wird vor Beginn des Patienteneinschlusses mit einem Randomisierungsprogramm durch einen nicht an der Studie beteiligten Biometriker durchgeführt. Dabei werden durch einen Rechenalgorithmus den Patientennummern zufällig die Therapietypen (Xenon oder Kontrolle) zugeordnet. Die zugeteilten Therapietypen werden anschließend in blickdichten Umschlägen, auf denen die

Studiennummer verzeichnet ist, verschlossen und bis zur Randomisierung des einzelnen Patienten sicher verwahrt.

Nach Einschluss des Patienten durch schriftliches Einverständnis nach Aufklärung durch den Studienarzt 1 erhält der Patient eine Studiennummer, mit Hilfe derer seine Daten während des Studienablaufs pseudonymisiert gesammelt und durch die befugten Personen (Biometriker, Monitor) eingesehen werden. Der Studiennummer wurde vor Beginn der Studie eine Therapieform (Xenon oder Kontrolle) wie oben beschrieben zugeordnet. Der Patient und der Auswerter sowie der Operateur (identisches Narkosegerät, keine Möglichkeit der zufälligen Entblindung) erfahren die Therapieform nicht. Einzig der die Narkose durchführende Studienarzt 2 und die zur Datenakquise anwesende Study Nurse können aufgrund der speziellen Durchführung der Xenon-Anästhesie nicht verblindet werden.

3.3 Aufbewahrung der Randomisierungs-Codes und Entblindung

3.3.1 Vorzeitige Entblindung

Während des Studienverlaufs ist eine komplette Verblindung gewährleistet. In einer Notfallsituation kann zum Schutz des Patienten vom Leiter der klinischen Prüfung oder vom Studienarzt die Blindung gebrochen werden. Die vorzeitige Entblindung der Studiennummer erfolgt nur im Falle eines AE/SAE, wenn dies medizinisch notwendig erscheint. Die vorzeitige Entblindung wird in der Patientenakte vermerkt und der Sponsor wird umgehend darüber informiert. Die Notfallmedikation und Therapie wird nach der medizinischen Notwendigkeit, dem medizinischen Wissensstand und der üblichen klinischen Praxis durchgeführt.

3.3.2 Reguläre Entblindung

Die reguläre Entblindung findet nach Abschluss der Studie d. h. nach Schließung der Datenbank statt.

3.4 Personelle und technische Anforderungen

3.4.1 Wissenschaftlerinnen und Wissenschaftler, mit denen für dieses Vorhaben eine konkrete Vereinbarung zur Zusammenarbeit besteht

Für dieses Projekt besteht eine konkrete Vereinbarung zur Zusammenarbeit mit der Klinik für Urologie, namentlich Univ.-Prof. Dr. Axel Heidenreich, dem Klinikdirektor,

und dem Oberarzt Dr. med. David Pfister. Die Klinik für Urologie führt die Nierenteilresektionen durch und betreut die Patienten medizinisch. Weiterhin besteht eine wissenschaftliche Kooperation mit dem Institut für Pathologie (Leitung: Univ.-Prof. Dr. med. Ruth Knüchel-Clarke). Durch das Institut für Pathologie wird im Rahmen der Regelversorgung die Größe und Histologie des Präparats sowie die Tumorklassifikation bestimmt sowie im Rahmen der Studie gegebenenfalls der Nachweis von HIF 1a im gesunden Randgewebe mittels Immunhistochemie und der Nachweis von KIM-1 oder NGAL in Urinproben mittels ELISA durchgeführt.
Eine weitere konkrete Vereinbarung zur Zusammenarbeit für dieses Projekt besteht mit dem Institut für Medizinische Statistik, Informatik und Epidemiologie (IMSIE) der Universität zu Köln (Direktor: Univ.-Prof. Dr. rer. nat. Walter Lehmacher). Das Institut stellt den für das Projekt verantwortlichen Biometriker, der für die Randomisierung, die GCP-konforme Datenüberprüfung, -speicherung und -auswertung zuständig ist.

3.4.2 Apparative Ausstattung

Die für das Projekt benötigten größeren Geräte sind in den projektbeteiligten Instituten und Kliniken vorhanden. Besonders ein spezieller Respirator zur Durchführung von Xenon-Anästhesien, mit dem auch die Kontrollanästhesie durchgeführt werden kann, ist in der Klinik für Anästhesiologie vorhanden (Gerät Felix Dual, Taema, ALMS, Frankreich). Das weitere Standardequipment für Anästhesien sowie die für die Operation und die histopathologische Auswertung ist in den genannten Kliniken vorliegend. Ein vollständig ausgestattetes und zertifiziertes labordiagnostisches Zentrum zur Messung der Laborparameter ist ebenfalls dem Universitätsklinikum angegliedert. In der Klinik für Anästhesiologie und im Institut für Medizinische Statistik, Informatik und Epidemiologie sind darüber hinaus genügend Rechner zur Dateneingabe, -überprüfung, -auswertung und zum Verfassen der notwendigen Schriftstücke vorhanden. Die benötigte Software (SPSS, Randomisierungsprogramm) ist ebenfalls vorliegend.

4 Studienpopulation

4.1 Studien- und Interventionsdauer

Die Studienteilnahme beträgt für jeden Patienten vom Moment der schriftlichen Einwilligung bis zum Entlasstag, maximal jedoch bis zum siebten postoperativen

Tag. Abhängig vom Zeitpunkt des Eingriffs sind das acht bis zehn Tage. Nach 3-6 Monaten soll eine erneute Bestimmung des Serum-Kreatinins und der GFR durch den behandelnden Urologen/Hausarzt erfolgen. Die Interventionsdauer entspricht dabei der für den Eingriff notwendigen Anästhesie, die üblicherweise drei Stunden beträgt. Die Gesamtdauer der Rekrutierungszeit (first patient in to last patient out) ist für sechzehn Monate vorgesehen.

4.2 Einschlusskriterien

Für den Einschluss in die klinische Prüfung müssen alle Einschlusskriterien erfüllt sein.

1. Männliche oder weibliche Patienten, ≥ 18 Jahre
2. Nierenkarzinom mit Beschränkung auf eine Niere
3. Operative Therapie: Nierenteilresektion
4. Eine durch den Patienten unterschriebene Einverständniserklärung, nach ausführlicher Aufklärung über die Studie durch eine Prüfarzt

4.3 Ausschlusskriterien

1. Chronische Niereninsuffizienz mit einer präoperativen GFR < 60 ml/min/1,73 m^2 KOF
2. American Society of Anesthesiologists (ASA) Status > III
3. Bekannte Kontraindikationen und Allergien für Propofol, Sufentanil, Xenon, Isoflurane, oder Rocuronium
4. Patienten mit schweren kardialen Vorerkrankungen (NYHA < III), einem akuten Koronarsyndrom innerhalb der letzten 24h, hämodynamischer Instabilität, Bedarf einer inotropen Unterstützung
5. Patienten mit einer schweren Lungen und Atemwegserkrankung (FeV1/FVC < 70% und FeV1 < 30% des Sollwertes), oder schwerer respiratorischer Insuffizienz mit einem PaO2 von <60mmHg, oder Heimsauerstofftherapie
6. schwere neurologische Vorerkrankungen
7. erhöhter intrakranieller Druck
8. Patienten mit einer Veranlagung zur malignen Hyperthermie
9. Frauen während Schwangerschaft / Stillzeit

10. Nicht-Einwilligungsfähigkeit
11. Ablehnung der Studie durch den Patienten
12. Gleichzeitige Teilnahme an weiteren Studien innerhalb der letzten 30 Tage
13. Sprach-/ Verständigungsprobleme im Rahmen der Aufklärung

4.4 Patientenausschlusskriterien und Prozeduren

4.4.1 Gründe für einen vorzeitigen Studienausschluss und Abbruch der Prüfproduktapplikation

- Die Sicherheit des Patienten steht an oberster Stelle. Zeigt sich nach Einschluss eines Patienten ein Ausschlusskriterium – aber vor der Intervention – (wie z.B. Änderung der Operationstechnik, akute Verschlechterung des Patienten, etc.), wird dieser Patient noch vor der Intervention ausgeschlossen. Die Patientendaten werden in einer Akte mit der Screening Nummer versehen im Studienaktenschrank aufbewahrt.
- Wird nachträglich – nach Beginn der Intervention – festgestellt, dass zum Zeitpunkt des Patienteneinschlusses bereits eine Verletzung der Ein- und Ausschlusskriterien vorlag, dann kommt es zu einem Studienabbruch. Abhängig zu welchem Zeitpunkt der Studienabbruch erfolgt, wird eine Standardnarkose durchgeführt und der Patient beim Data-Management ausgeschlossen.
- Rücknahme der Einwilligung: Patienten können jederzeit und ohne Angabe von Gründen ihre Einwilligung zurückziehen und die Studie abbrechen. Die bis dahin erhobenen Daten und wofür ein Patient randomisiert wurde und der Zeitpunkt des Zurückziehens seiner Einwilligung muss in der Dokumentation festgehalten bleiben. Der Patient ist darüber zu informieren, dass im Falle eines Widerrufs der Einwilligung die gespeicherten Daten weiterhin verwendet werden dürfen.
- Schwere unerwünschte Ereignisse (SAE) und Verdachtsfälle einer unerwarteten schwerwiegenden Nebenwirkung (SUSAR), welche eine Fortsetzung der klinischen Prüfung verbieten.
- Unerwünschte Ereignisse (AE) und Nebenwirkungen (AR), welche durch den Hauptprüfer bei Fortsetzung der klinischen Studie, als gefährlich für die physische und psychische Gesundheit eines Patienten angesehen werden.

- Technische Schwierigkeiten und erforderliche Veränderungen der Geräte bei der Verabreichung der Prüfmedikation.

4.4.2 Zeitpunkt des Patientenausschlusses aus der Studie / dem Einfluss des Studienmedikamentes

Jeder Patient kann aus der Studie jederzeit ausgeschlossen werden. Wenn sich ein Zustand wie oben genannt an irgendeinem Zeitpunkt ereignet, wird der Patient sofort aus der Studie ausgeschlossen. Falls sich dies während einer laufenden Anästhesie ereignet, wird die Verabreichung des Studienmedikamentes sofort unterbrochen und das Studienmedikament aus dem Patienten ausgewaschen und durch ein Standardanästhetikum ersetzt. Der Patient wird sofort entsprechend seinem klinischen Bedarf medizinisch behandelt.

4.4.3 Dokumentation des Zeitpunktes und der Art des Patientenausschlusses

Alle bis zum Ausschluss der Person aus der klinischen Studie erhobenen Daten werden gesammelt und dokumentiert. Der Ausschlussgrund und der genaue Zeitpunkt und die spezifischen Details des Ausschlusses einer Prüfperson werden auf einem Sonderbogen des CRF`s erfasst. Der Prüfer muss den Haupt-Ausschlussgrund der Prüfperson benennen. Im Falle eines SAE/SUSAR, wird der Sponsor innerhalb von < 24h nach bekannt werden informiert. Der Sponsor wird, falls erforderlich, das SAE/SUSAR sofort der Bundesoberbehörde und der Ethikkomission melden.

4.4.4 Ersatz von ausgeschlossenen Patienten

Nach ihrer Randomisierung ausgeschlossene Patienten werden ersetzt.

4.4.5 Weiterverfolgung von ausgeschlossenen Patienten

Von der Studie/dem Prüfmedikament ausgeschlossene Patienten verbleiben weiterhin Patienten des Universitätsklinikums Aachen und werden von den zuständigen Ärzten entsprechend dem medizinischen Wissenstand und der klinischen Praxis weiterbehandelt. Falls der Patient in eine spezielle Einrichtung verlegt wird, oder entlassen wird, kann die Klinik für Anästhesiologie 24h täglich durch den Patienten erreicht werden. Die Kontaktdaten hierfür werden dem Patienten mit den Aufklärungsunterlagen ausgehändigt. Jedem Patienten, welcher seine

Einverständniserklärung nach der Intervention zurückzieht, wird ausdrücklich angeraten an einer Abschlussuntersuchung teilzunehmen.

5 Individueller Studienablauf

5.1 Prüfung der Ein- und Ausschlusskriterien

Die Prüfung der Ein- und Ausschlusskriterien potentieller Studienpatienten ("Screening") erfolgt anhand einer vorgefertigten Checkliste:
Alle Patienten, welche die Einschlusskriterien:

- Nierenkarzinom mit Beschränkung auf eine Niere
- Operative Therapie: Nierenteilresektion
- Alter ≥ 18 Jahre

erfüllen, werden bereits durch die Kollegen der Klinik für Urologie über die Möglichkeit an einer Studienteilnahme informiert. Bei Interesse werden sie von einem Studienarzt visitiert und über die Studiendurchführung und den Nutzen und das Risiko der Studie inklusive einer individuellen Nutzen-Risiko-Abwägung aufgeklärt. In diesem Gespräch eruiert der Studienarzt ebenfalls, ob Ausschlusskriterien für eine Studienteilnahme vorliegen. Die Patienten erhalten dann das Patienteninformationsblatt und individuell ausreichend Bedenkzeit. Ein Screening Log wird geführt. Wenn ein Patient alle Einschlusskriterien erfüllt und keine Ausschlusskriterien vorliegen ("eligible"), ist im Screening-Log zu dokumentieren, ob ein Einschluss in die klinische Prüfung erfolgt ist ("enrolled"). Falls der Patient nicht in die klinische Prüfung eingeschlossen wird, so ist im Screening-Log mindestens 1 Ausschlusskriterium zu dokumentieren. Jeder einschlussfähige Patient ist zu dokumentieren. Im Rahmen der Überprüfung der Ein- und Ausschlusskriterien dokumentiert der Prüfarzt, dass der Patient aktuell und auch in den vorangegangenen 30 Tagen nicht an einer anderen klinischen Prüfung oder einem anderen Forschungsprojekt teilnimmt/teilgenommen hat. Hierfür wird die Patientenakte sorgfältig auf entsprechende Angaben überprüft. Es wird dem Patienten erklärt, dass der Patient nicht an einer anderen klinischen Prüfung oder einem anderen Forschungsprojekt teilnehmen darf, da eine gleichzeitige Teilnahme an mehreren klinischen Prüfungen oder Forschungsprojekten unkalkulierbare Risiken für den Patienten darstellen können.

5.2 Patientenaufklärung

Jeder potenziell geeignete Patient muss umfassend über die Studie und deren Zielsetzung, die Freiwilligkeit der Teilnahme, die Möglichkeit des Rücktritts ohne Begründung, die pseudonymisierte Verwendung und Veröffentlichung der Ergebnisse, die Risiken und möglichen Komplikationen aufgeklärt werden. Die Aufklärung erfolgt mündlich durch den Prüfarzt und schriftlich durch die Patienteninformation. Nachdem dem Patienten ausreichend Zeit eingeräumt wurde Fragen zu stellen und die Teilnahme an der Prüfung zu überdenken, wird dieser gebeten zwei Exemplare der Einwilligungserklärung zu unterzeichnen und eigenhändig zu datieren. Dem Patienten wird anschließend ein Exemplar der Patienteninformation bzw. der Einwilligungserklärung ausgehändigt, das zweite Exemplar wird im Prüfarztordner aufbewahrt.

5.3 Visiten

Es werden insgesamt höchstens 11 Visiten durchgeführt. Die Visiten 0 und 2-10 werden durch den Studienarzt 1 (Investigator I) verblindet bezüglich der Studienmedikation durchgeführt. Einzig der die Narkose durchführende Studienarzt 2 (Investigator II) und die zur Datenakquise anwesende Study Nurse können aufgrund der speziellen Durchführung der Xenon-Anästhesie nicht verblindet werden.

5.3.1 Visite 0 (Baselinemessung)

Nach Erhalt des schriftlichen Einverständnisses findet die Baseline-Untersuchung durch den Studienarzt 1 statt. Diese beinhaltet neben einer gründlichen Anamnese und körperlichen Untersuchung zum Aufdecken von Ausschlusskriterien die Erfassung von demographischen Daten (Geschlecht, Körpergröße, Gewicht, Alter, ASA-Status u.a.), Ausgangsdaten der Vitalparameter (Herzfrequenz, Blutdruck, Sauerstoffsättigung unter Raumluft), der Laborparameter (kleines Blutbild, Gerinnungsparameter, Serum-Harnstoff, Transaminasen, Creatinkinase), und besonders der präoperativen GFR (bestimmt mit dem Cystatin C-Wert im Serum) und des Serum-Kreatinins. Des Weiteren wird eine Blutprobe zur Bestimmung des MIF entnommen. Eine Urinprobe wird asserviert.

Die Daten werden in einem Papier-Case-Report Form (CRF) händisch erfasst. Liegen Ausschlusskriterien vor, werden die Patienten nicht eingeschlossen. Ihre Daten werden in einer Akte mit der Screening Nummer versehen im Studienaktenschrank aufbewahrt. Liegen keine Ausschlusskriterien vor, erhält der Patient eine Studiennummer. Dieser Nummer ist bereits vor Beginn der Studie (s.o.) eine Behandlungsform, verschlossen in einem blickdichten Umschlag, zugeordnet worden.

5.3.2 Visite 1 (Operationstag)

Kurz vor der Anästhesieeinleitung wird durch den Studienarzt 2 der dem Patienten entsprechende Randomisierungsumschlag geöffnet, nur der die Narkose durchführende Studienarzt und die unter seiner Aufsicht Daten erhebende Study Nurse haben Zugang zu dieser Information.

Nach Anlage des für eine Anästhesie üblichen Monitorings, erhalten alle Patienten zur post-operativen Schmerztherapie vor Allgemeinanästhesie einen thorakalen Periduralkatheter.

Einleitung:

Die Narkose wird in beiden Fällen (Xenon und Kontrolle) mit Propofol (1,5-2mg/kgKG und Sufentanil (0,2µg/kg KG) eingeleitet; als Relaxans erhalten die Patienten eine gewichtsadaptierte Dosis Rocuronium (0,6 mg/kgKG).

Narkoseaufrechterhaltung:

Nach der Intubation erhalten die Patienten der Xenon-Gruppe eine Allgemeinanästhesie mit 60 % insp. Xenon/ 40% O2, die Patienten der Kontrolle eine Anästhesie mit 1.2 Vol. % et Isoflurane in einem 40 %O2 enthaltendem O2/Luftgemisch; beide supplementiert mit Sufentanil titriert nach klinischem Bedarf.

Operation:

- MAD > 65mmHg; Zur Verhinderung einer zusätzlichen Einschränkung der Nierenfunktion aufgrund unzureichender Perfusion wird der arterielle Mitteldruck kontinuierlich über 65 mmHg, gegebenenfalls mit Hilfe von titriertem Noradrenalin, gehalten.
- Die Operation wird nach den Standards der Klinik für Urologie durchgeführt.

- Währenddessen werden die Anästhesie- und Sicherheitsdaten in fünf minütigen Abständen, die Urinausscheidung stündlich und die Gesamtmenge am Ende der Anästhesie dokumentiert.
- Die Schnitt-Naht-Zeit, der intraoperative Blutverlust, die Warmischämiezeit als Tumorresektionszeit und die Exposition mit Anästhetika bis zum Beginn direkten Tumorresektionszeit und nach Ende dieser, (entsprechend dem Ende der Versorgung des Tumorgrundes), bis zur Ausleitung der Narkose werden in Zusammenarbeit des Studienarztes mit dem Operateur bestimmt.
- Das Tumorpräparat wird in das Institut für Pathologie gegeben, und dort nach einem Schnellschnitt auf Tumorfreiheit der Schnittränder der weiteren Diagnostik zugeführt. Die Bestimmung der Größe des Präparats, der Histologie mittels Immunhistochemie im Paraffinschnitt und ggf. der Aktivierung von HIF 1a im tumorfreien Rand des Präparats mittels Immunfluoreszenzfärbung erfolgt durch das Institut für Pathologie.
- ca. 5 Min. nach Ende der Ischämiezeit ≙ Ende der Versorgung des Tumorgrundes, erfolgt eine Blutentnahme zur Bestimmung des macrophage migration-inhibiting factor (MIF) im Serum.

<u>Aufwachraum (AWR):</u>

- Im AWR werden die invasiven Blutdruckwerten (RR_{sys}, RR_{dias}, RR_{MD}) und die HF alle 15 Min. erfasst und das EKG und die Pulsoxymetrie werden kontinuierlich abgeleitet.
- Bei Aufnahme in den AWR erfolgt eine weitere Blutentnahme zur Messung des macrophage migration-inhibiting factor (MIF) im Serum

Der Studienarzt 2 trägt alle erhobenen Daten per Hand in das Papier-CRF des Patienten ein.

Die Nachobservationszeit beträgt innerklinisch maximal sieben Tage nach dem Eingriff. In sechsstündigen Abständen ab Ende der Operation bis zum Ende der ersten postoperativen Tages wird die Urinausscheidung des Patienten in sechsstündigen Abständen bestimmt; dazu wird die Urinmenge im Stundenurometer am standardmäßig für den Eingriff eingelegten Urindauerkatheter abgelesen. Am 2. postoperativen Tag wird die Gesamturinausscheidung des Tages abgelesen. Am 1. und 2. sowie am 7. postoperativen Tag werden Urinproben asserviert, aus denen

ggf. KIM-1 oder NGAL mittels ELISA bestimmt werden. Am 1. bis zum Entlasstag bzw. maximal 7. postoperativen Tag erfolgen täglich eine morgendliche Blutentnahme und eine Messung der Vitalparameter (Blutdruck, Herzfrequenz, Sauerstoffsättigung) durch den Studienarzt. Aus den Blutproben wird im Labor Cystatin C bestimmt, und daraus die GFR berechnet. Weiterhin wird der sekundäre Outcome-Parameter Serum Kreatinin bestimmt, sowie die Sicherheitsparameter (kleines Blutbild, Gerinnungsparameter, Serum-Harnstoff, Transaminasen, Creatinkinase) und in einer weiteren Blutentnahme wird am 1. postoperativen Tag der macrophage migration-inhibiting factor (MIF) im Serum erneut bestimmt. Laborparameter sowie die Urinmenge und die Vitalparameter werden durch einen Studienarzt bewertet und ins CRF eingetragen. Der Studienarzt bestimmt ebenfalls das Vorliegen eines akuten Nierenversagens nach den Kriterien der AKIN (34) am ersten und zweiten postoperativen Tag. Nach einer körperlichen Abschlussuntersuchung durch den Studienarzt endet die innerklinische Studienteilnahme für die Patienten am Entlasstag bzw. spätestens siebten postoperativen Tag. 3-6 Monate postoperativ werden wir versuchen die GFR und den Serum-Kreatininwert über den behandelnden Urologen/Hausarzt des Patienten erneut zu bestimmen.

5.3.3 Visite 2 (Op-Tag, postoperativ)

- Bestimmung der Urinausscheidung des Patienten alle 6 h postoperativ. Die Urinmenge wird dazu im Stundenurometer am standardmäßig für den Eingriff eingelegten Urindauerkatheter abgelesen.
- Erfassung und Dokumentation sämtlicher AE`s und SAE`s

5.3.4 Visite 3 (1. postoperativer Tag)

- Bestimmung der Urinausscheidung des Patienten alle 6 h postoperativ. Die Urinmenge wird dazu im Stundenurometer am standardmäßig für den Eingriff eingelegten Urindauerkatheter abgelesen bis zum 2. postoperativen Tag.
- Am Morgen des ersten postoperativen Tages erfolgt eine Blutentnahme und eine Messung der Vitalparameter (Blutdruck, Herzfrequenz, Sauerstoffsättigung) durch den Studienarzt 1.

- Hierbei benötigte Laborwerte: Cystatin C, woraus die GFR berechnet wird. Weiterhin wird der sekundäre Outcome-Parameter Serum Kreatinin bestimmt, sowie die Sicherheitsparameter (kleines Blutbild, Gerinnungsparameter, Serum-Harnstoff, Transaminasen, Creatinkinase).
- Eine letzte Blutprobe zur Bestimmung des macrophage migration-inhibiting factor (MIF) im Serum wird entnommen.
- Eine Urinprobe zur ggf. Bestimmung von KIM-1 oder NGAL wird asserviert.
- Laborparameter sowie die Urinmenge und die Vitalparameter werden durch einen Studienarzt bewertet und ins CRF eingetragen. Der Studienarzt bestimmt ebenfalls das Vorliegen eines akuten Nierenversagens nach den Kriterien der AKIN-Definition (34) am ersten und zweiten postoperativen Tag.
- Erfassung und Dokumentation sämtlicher AE`s und SAE`s

5.3.5 Visite 4 - 8 (2. – 6. postoperativer Tag)

- Morgendliche Blutentnahme und eine Messung der Vitalparameter (Blutdruck, Herzfrequenz, Sauerstoffsättigung) durch den Studienarzt 1.
- Am 2. postoperativen Tag wird zusätzlich die Tagesgesamt-Urinmenge bestimmt.
- Am 2. postoperativen Tag wird eine Urinprobe asserviert.
- Zu bestimmende Laborwerte: s. 5.3.4
- Laborparameter und die Vitalparameter werden durch einen Studienarzt bewertet und ins CRF eingetragen.
- Der Studienarzt bestimmt ebenfalls das Vorliegen eines akuten Nierenversagens nach den Kriterien der AKIN-Definition (34) am zweiten postoperativen Tag.
- Erfassung und Dokumentation sämtlicher AE`s und SAE`s

5.3.6 Visite 9 (7. postoperativer Tag bzw. am Entlasstag / Studienende)

- Nach einer körperlichen Abschlussuntersuchung, Bestimmung der Vitalparameter, Asservation einer Urinprobe und Blutentnahme (zu bestimmende Laborwerte s. 5.3.4) durch den Studienarzt 1 endet die Studienteilnahme für die Patienten am siebten postoperativen Tag.
- Erfassung und Dokumentation sämtlicher AE`s und SAE`s

5.3.7 Visite 10 (3-6 Monate postoperativ)

- Kontaktierung des behandelnden Urologen/Hausarztes mit der Bitte um Kontrolle des Kreatinins im Serum und Bestimmung der GFR im Rahmen der Routine-Blutentnahme im rahmen der postoperativen Nachsorgeuntersuchung.

6 Prüfmedikation

6.1 Bezeichnung

- Xenon 100% (v/v)
- Isoflurane

6.2 Verabreichung der Prüfmedikation

Über einen speziellen Respirator mit einem geschlossenen System wird sowohl die Xenon-Anästhesie, als auch die Kontrollanästhesie durchgeführt. (Gerät Felix Dual, Taema, ALMS, Frankreich).

6.3 Verpackung, Bezeichnung und Aufbewahrung der Prüfmedikation

Das medizinische Xenon (LenoXe®, 100% v/v, Air Liquide) wird in Stahlzylindern entsprechend den europäischen Regeln bezeichnet geliefert. Es wird bei Raumtemperatur aufbewahrt, hitze- und lichtgeschützt aufbewahrt. Isoflurane (250ml, Firma Abbott) wird in speziell gekennzeichneten Flaschen bereitgestellt und bei 4℃ in einer lichtgeschützten Box aufbewahrt. Dieses wird durch spezifische wiederauffüllbare Vaporen vaporisiert.

6.4 Verantwortung des Umgangs mit der Prüfmedikation

Das Prüfprodukt Xenon (LenoXe®) wird durch die Air Liquide Medical GmbH (Hans-Günther-Sohl-Str.5; D-40235 Düsseldorf) geliefert. Das zweite Prüfprodukt Isoflurane (Forene®) wird durch die Firma Abbott geliefert. Beide werden durch die Zentralapotheke (Universitätsklinikum Aachen; Leiter: Dr. rer. nat. Albrecht Eisert; Steinbergweg 20; D-52074 Aachen) bestellt und herausgegeben. Die Verantwortung

der korrekten Anwendung der Prüfmedikamente entsprechend der Fachinformation und in Übereinstimmung mit der DGAI (Deutschen Gesellschaft für Anästhesiologie und Intensivmedizin), verbleibt in der Abteilung für Anästhesiologie des Universitätsklinikums Aachen.

6.5 Kriterien für einen vorzeitigen Studienabbruch aufgrund der eingesetzten Prüfmedikation

Die in dieser Studie eingesetzten Prüfpräparate, sind Medikamente, welche täglich zur Durchführung einer Vollnarkose eingesetzt werden. Sie sind seit vielen Jahren in Deutschland zugelassen, wurden in vielen Studien überprüft und werden sicher in der täglichen Anästhesiedurchführung eingesetzt. Somit werden Abbruchgründe aufgrund der Studienmedikation für die Studie nicht erwartet.

6.6 Bezeichnung der restlichen Studienmedikamente

- Propofol
- Sufentanil
- Rocuronium
- Sauerstoff
- Medizinische Luft

6.7 Verantwortung über die restliche Studienmedikation

Die restliche Studienmedikation wird durch die Zentralapotheke (Universitätsklinikum Aachen; Leiter: Dr. rer. nat. Albrecht Eisert; Steinbergweg 20; D-52074 Aachen) bestellt und herausgegeben. Die Verantwortung der korrekten Anwendung der Prüfmedikamente entsprechend der Fachinformation und in Übereinstimmung mit der DGAI (Deutschen Gesellschaft für Anästhesiologie und Intensivmedizin), verbleibt in der Abteilung für Anästhesiologie des Universitätsklinikums Aachen.

6.8 Begleittherapie

Die angeordnete Begleitmedikation des Patienten für seine Begleiterkrankungen wird entsprechend der Prüfarztentscheidung eingenommen. Es gibt keine Anwendungseinschränkung der Begleitmedikation vor oder während der Studie.

6.9 Unerlaubte Medikation

Während der Visite 1 (Allgemeinanästhesie) sollen keine anderen volatilen Anästhetika als die oben bezeichneten Prüfpräparate eingesetzt werden.

7 Beurteilung der Wirksamkeit

7.1 Bezeichnung der Wirksamkeitsparameter

7.1.1 Auswahlkriterien und demographische Daten

Die folgenden demographischen Daten sollen erfasst werden:

- Geburtsdatum
- Geschlecht
- Größe und Gewicht
- Vorerkrankungen, Nebenerkrankungen mit Medikation, Voroperationen, Rauchgewohnheiten, Alkoholkonsum und/ oder Drogenkosum, Allergien
- Der ASA (America Society of Anesthesiologists) Status
- Zeitpunkt der Krankenhaus und Stationsaufnahme
- Geplanter OP-Zeitpunkt

7.1.2 Primäre Wirksamkeitskriterien

Die frühe postoperative Nierenfunktion nach einer Xenonnarkose im Vergleich zu einer Isoflurannarkose während einer Nierenteilresektion wird gemessen durch:

- Die Bestimmung der GFR mit Hilfe des Cystatin C Wertes im Serum präoperativ und an den folgenden 7 postoperativen Tagen. Der maximale „GFR-Abfall“ wird dabei bestimmt als Differenz aus der präoperativen Ausgangs-GFR minus der niedrigsten postoperativen GFR innerhalb dieser sieben postoperativen Tage.

7.1.3 Sekundäre Wirksamkeitskriterien

Die folgenden sekundären Parameter werden zusätzlich zur Beurteilung der Xenonnarkose im Vergleich zur Isoflurannarkose erfasst:

- Messung der Anästhesie- und Sicherheitsdaten alle 5 Min. und Bestimmung der Urinmenge am Ende der Operation.
- Bestimmung des Vorliegens eines akuten Nierenversagens nach den AKIN-Kriterien durch Ablesen der Urinmenge alle 6 h bis zum 2.postoperativen Tag, der Gesamturinmenge am 2. postoperativen Tag und Bestimmung des Kreatininwertes an den ersten 7 postoperativen Tagen, bzw. bis zur Entlassung, falls der Patient früher entlassen werden sollte.
- Tägliche Messung der Sicherheitsparameter (kleines Blutbild, Gerinnungsparameter, Serum-Harnstoff, Transaminasen, Creatinkinase) bis zum 7. postoperativen Tag, bzw. bis zum Entlasstag, falls der Patient früher entlassen werden sollte.
- Tägliche Messung der Vitalparameter (Blutdruck, Herzfrequenz, Sauerstoffsättigung) bis zum 7. postoperativen Tag, bzw. bis zum Entlasstag, falls der Patient früher entlassen werden sollte. .
- Histologischen Untersuchung mittels Immunhistochemie im Paraffinschnitt und ggf. Aktivitätsbestimmung von HIF-1α im tumorfreien Rand des Präparats mittels Immunfluoreszenzfärbung.
- Untersuchung der Freisetzung von macrophage migration-inhibiting factor (MIF) im Serum.
- Bestimmung von KIM-1 oder NGAL im Urin
- Erfassung des intraoperativen Blutverlustes.
- Messung der Expositionsdauer mit den Anästhetika bis zum Beginn der operativen Ischämiephase (i.e.S der Tumorresektionszeit) und nach Ende dieser, bis zur Ausleitung der Narkose. Dies wird in Zusammenarbeit des Studienarztes mit dem Operateur bestimmt.
- Ermittlung der GFR und des Serum-Kreatinins nach 3-6 Monaten postoperativ.

7.2 Messung, Registrierung und Analyse der Wirksamkeitskriterien

Die Auswahlkriterien und demographische Daten werden während der Visite 0 durch den Studienarzt 1 erfasst und analysiert. Die intraoperativen Daten werden durch den unverblindeten Studienarzt 2 in der Visite 1 gemessen, registriert und analysiert. Die restlichen Untersuchungen der sekundären Wirksamkeitskriterien, postoperativ bis zum 7. postoperativen Tag, bzw. bis zum Entlasstag, falls der Patient zuvor entlassen werden sollte, werden durch den verblindeten Studienarzt 1 durchgeführt. Zur Gewährleistung der Verblindung, werden für jeden Studienpatient zwei Case-Report-Forms (CRF`s) mit allen erhobenen Daten und zusätzlichen Patienteninformationen angelegt. Ein CRF für die Visiten 0, 2–10 und einer für die Visite 1. Daten, welche von den normalen Grenzwerten abweichen, werden durch den Prüfarzt bewertet und als klinisch signifikant (cs) oder nicht klinisch signifikant (ncs) eingestuft. Jeder klinisch signifikante Wert wird als unerwünschtes Ereignis (AE) in Betracht gezogen und folglich als solches unter dem Abschnitt unerwünschter Ereignisse dokumentiert.

8 Sicherheitsanalysen

8.1 Sicherheitsmassnahmen während der Allgemeinanästhesie zur Nierenteilresektion (Visite 1)

8.1.1 Verabreichung der Prüfmedikation

Die Konzentration und Dosis der Prüfmedikation wird bei jedem Patienten erfasst:

- Messung der inspiratorischen Konzentration und der Gesamtdauer der Xenon und Isofluraneexposition der Patienten.
- Bestimmung der Expositionsdauer mit den Prüf-Anästhetika bis zum Beginn der direkten Tumorresektionszeit und nach Ende dieser, bis zur Ausleitung der Narkose.
- Der Gesamtverbrauch von Xenon (in Litern)
- Die Einleitungsdosis von Propofol für die Vollnarkose
- Die Gesamtdosis des verabreichten Sufentanils während der ganzen Vollnarkose
- Die Gesamtdosis des verabreichten Rocuroniums während der ganzen Vollnarkose

8.1.2 Klinische Sicherheitsparameter

- Systolischer und diastolischer Blutdruck, Mitteldruck und die Herzfrequenz werden alle 5 Minuten von Eintreffen in den OP-Saal bis zum Verlassen des OP-Saals und alle 15 Minuten im Aufwachraum dokumentiert.
- Das EKG wird sowohl im OP-Saal als auch im Aufwachraum kontinuierlich abgeleitet. Der Zeitpunkt einer klinisch signifikanten EKG-Veränderung wird durch den Studienarzt 2 registriert. Sollten diese EKG-Veränderungen klinisch-signifikant sein, werden sie durch den Studienarzt 2 als seperate AE`s dokumentiert und ihre Art, Schwere und die ggf. durchgeführte medizinische Maßnahme wird erfasst.
- Die Sauerstoffsättigung wird mittels Pulsoxymetrie kontinuierlich von Eintreffen in den OP-Saal bis zum Verlassen des OP-Saals und im Aufwachraum gemessen und alle 15 Min. dokumentiert.
- Die Beatmungsparameter werden mit Hilfe des Felix Dual® durch den Studienarzt 2 alle 15 Minuten während der Vollnarkose notiert (Fi $O_{2,}$ Sättigung, Fe O_2, CO_{2et}, AMV, AF, Beatmungdrücke).
- Der Cuffdruck wird während der Beatmung alle 5 Min. kontrolliert und auf einen Wert von 25-30 cmH_2O eingestellt.
- Die Urinmenge und der Blutverlust werden stündlich und am Ende der Operation abgelesen.
- Erfassen der Schnitt-Naht-Zeit und der Warmischämiezeit, als Zeit der direkten Tumorresektion bis zum Ende der Versorgung des Tumorgrundes, in Zusammenarbeit des Studienarztes mit dem Operateur.
- Das Auftreten von AE`s und SAE`s wird GCP konform bei jeder Visite erfasst und an der entsprechenden Stelle im CRF dokumentiert.

8.2 Sicherheitsmassnahmen während der Visiten 0 und 2-9

8.2.1 Klinische Sicherheitsparameter und Untersuchungen

Die Patientensicherheit wird durch eine engmaschige klinische Untersuchung und die Bestimmung der folgenden Parameter auf jeder Visite des Studienarztes 1 vom Einschluss (Visite 0) und der 1. postroperativen Visite (Visite2) bis zum Studienende (Visite 9) sichergestellt.

- Messung der Vitalparameter (Blutdruck, Herzfrequenz, Sauerstoffsättigung).
- Bestimmung der Urinausscheidung alle 6h postoperativ bis zum 2. postoperativen Tag. Und 24h am 2. und 3. postoperativen Tag.
- Erfassung eines akuten Nierenversagens anhand der AKIN Klassifikation.
- Das Auftreten von AE`s und SAE`s wird GCP konform bei jeder Visite erfasst und an der entsprechenden Stelle im CRF dokumentiert.

8.2.2 Blutentnahmen und Laborparameter

Bei jedem Patienten werden Blutproben an den folgenden Zeitpunkten entnommen:

- Die Baseline-Probe; zwischen der Auswahl (Visite 0) und der Allgemeinanästhesie (Visite1)
- In der postoperativen Phase; einmal täglich an den ersten 7 postoperativen Tagen, bzw. bis zum Entlasstag, falls der Patient zuvor entlassen werden sollte (Visite 2-9).
- Zusätzliche Blutproben werden entsprechend der Entscheidung des Studienarztes oder anderer Ärzte entnommen. Z.B. im Falle von technischen Problemen während der initialen Untersuchung oder dem Verlust der Baseline-Proben vor der Operation und/oder bei klinische Abweichungen, welche zusätzliche Messungen erfordern.

In jeder Blutprobe werden die folgenden Parameter erfasst:

- Hämatologische Parameter: Erythrozyten, Hämoglobin, Hämatokrit, Leukozyten, Thrombozyten
- Nierenwerte: Kreatinin im Serum, Harnstoff, Cystatin-C Wert im Serum zur Berechnung der GFR
- Gerinnungswerte: Quick, INR, PTT
- Leberwerte: ALT und AST
- Als weiterer Sicherheitsparameter: Creatinkinase = CK

(Visite 10): Nach 3-6 Monaten erfolgt eine erneute Blutentnahme mit Bestimmung von Kreatinin und Cystatin C zur Berechnung der GFR über den behandelnden Urologen/Hausarzt im Rahmen der postoperativen Nachsorgeuntersuchung.

8.3 Bewertung, Aufzeichnung und Analyse der Sicherheitsparameter

Die Patienten werden täglich bis zum 7. postoperativen Tag, bzw. bis zum Entlasstag, wenn dieser zuvor stattfindet von einem speziell ausgebildeten

Studienarzt visitiert. Für jedes Ereignis welches sich zwischen diesen Visiten ereignet, wird ein Studienarzt des Studienteams per Telefon zur Verfügung stehen. Alle erhobenen Patiendaten werden im CRF dokumentiert und durch den Studienarzt entsprechend den SOP`s (Standard Operating Procedures) unseres Krankenhauses und Normwerttabellen (s. Anhang) bewertet. Daten, welche von den normalen Grenzwerten abweichen, werden durch den Studienarzt evaluiert und als klinisch signifikant (cs) oder nicht klinisch signifikant (ncs) eingestuft. Jeder klinisch signifikante Wert wird als unerwünschtes Ereignis (AE) erachtet und folglich als solches unter dem Abschnitt unerwünschter Ereignisse dokumentiert und der Sponsor wird über die AE`s in Kenntnis gesetzt.

8.4 Umgang mit unerwünschten Ereignissen (AE)

Ein unerwünschtes Ereignis (Adverse Event, AE) ist jedes nachteilige medizinische Vorkommnis, das einer betroffenen Person (Prüfungsteilnehmer) widerfährt, der ein Prüfpräparat verabreicht wurde, und das nicht notwendigerweise in ursächlichem Zusammenhang mit dieser Behandlung steht. Dies können Erkrankungen, Krankheitszeichen oder Symptome sein, die nach Einschluss des Patienten in die Studie auftreten oder sich verschlechtern (GCP-V §3 Abs. 6). Falls AE´s nicht durch die Patienten selbst oder das Stationspersonal mitgeteilt werden, werden sie durch die sorgfältige klinische Untersuchung, die Ermittlung von Sicherheitswerten und eine nicht-suggestive Patientenbefragung durch den jeweiligen Studienarzt erhoben und gründlich dokumentiert.

Hat ein Studienarzt zu irgendeinem Zeitpunkt der Studiendurchführung Anhalt für ein AE, so werden unverzüglich alle Maßnahmen ergriffen, um dieses AE zu diagnostizieren und die notwendigen medizinischen Maßnahmen zur Behandlung einzuleiten. Die Patienten werden bei Eintritt eines AE entsprechend ihren medizinischen Bedürfnissen überwacht und behandelt, bis die Symptome verschwinden, sich die Laborwerte normalisieren und die beobachteten Effekte ausreichend erklärt werden können. Die Patienten verbleiben solange stationär, bis sie den Abteilungs– und Klinikstandard zur Entlassung erfüllen.

Der Studienarzt nimmt zusätzlich eine Bewertung vor, ob das AE im Zusammenhang mit der Studienmedikation steht oder nicht. Folgendes wird im CRF dokumentiert:

- Art des Unerwünschten Ereignisses (Anzeichen, Symptom oder Krankheit)
- Differenzierung (schwerwiegend/nicht schwerwiegend)

- Beginn und Ende des Auftretens
- Intensität
- Kausalität zum Prüfpräparat
- Maßnahmen hinsichtlich des Prüfpräparates oder Handlungen zur Wiederherstellung oder Besserung des Wohlbefindens des Patienten
- Ausgang des Ereignisses.

Die Intensität der Beschwerden bzw. des unerwünschten Ereignisses wird nach folgendem Schema klassifiziert:

- **Leicht:** Wahrnehmung von Anzeichen oder Symptomen, die leicht zu ertragen sind. Symptome können leicht ignoriert werden und verschwinden, sobald die Aufmerksamkeit auf andere Dinge gelenkt wird.
- **Mäßig:** Symptome rufen Unwohlsein hervor, sind aber tolerabel. Sie können nicht ignoriert werden und beeinträchtigen die Konzentration.
- **Stark:** Heftige Symptome, welche die normalen täglichen Aktivitäten beeinflussen.

Über alle AE`s wird der Sponsor in Kenntnis gesetzt.

8.5 Umgang mit schwerwiegenden unerwünschten Ereignissen (SAE)

Als schwerwiegend unerwünschtes Ereignis, (Serious Adverse Event, SAE) bzw. Schwerwiegende Nebenwirkung (Serious Adverse Reaction, SAR) wird jedes unerwünschte Ereignis oder jede Nebenwirkung bezeichnet, das bzw. die

- zum Tode führt oder
- lebensbedrohlich ist oder
- zu bleibender oder schwerwiegender Behinderung oder zu Invalidität führt oder
- eine stationäre Behandlung oder deren Verlängerung erforderlich macht oder
- zu angeborenen Missbildungen oder Geburtsfehlern führt.

Im Falle eines SAE/SAR wird neben den unter 8.5 beschriebenen medizinischen Maßnahmen und der Dokumentation im CRF das SAE/SAR unverzüglich (< 24h) an den Sponsor durch den Studienarzt gemeldet. Bei vermutetem oder gesichertem Zusammenhang mit der Prüfmedikation informiert der Sponsor innerhalb des gesetzlich vorgegebenen Zeitraums die Bundesoberbehörde (BfArM) (s. 8.6).

Nach Meldung übermittelt der Studienarzt hierzu dem Sponsor einen ausführlichen schriftlichen Bericht.

8.6 Umgang mit suspekten unerwarteten schwerwiegenden Nebenwirkung (SUSAR)

Eine Nebenwirkung (Adverse Reaction, AR) ist jede nachteilige und unbeabsichtigte Reaktion auf ein Prüfpräparat, unabhängig von dessen Dosierung. Eine Unerwartete Nebenwirkung (Unexpected Adverse Reaction, UAR) ist eine Reaktion für die ein Kausalzusammenhang mit der Prüfmedikation besteht, die nach Art oder Schweregrad aber nicht mit der vorliegenden Information über das Prüfpräparat übereinstimmt. Bei Feststellung einer suspekten unerwarteten schwerwiegenden Nebenwirkung (Suspected Unexpected Serious Adverse Reaction, SUSAR) wird nach der Richtlinie 2001/20/EG der Sponsor unvermittelt informiert und dieser meldet den Fall innerhalb von max. 15 Tagen an die zuständige Ethik-Kommission und die zuständige Bundesoberbehörde (BfArM) weiter (§13, Abs.2 GCP-V). Dieser Zeitraum verkürzt sich auf max. 7 Tage, wenn der Verdachtsfall einer unerwarteten schwerwiegenden Nebenwirkung, zu einem Todesfall geführt hat oder lebensbedrohlich ist (§13, Abs.3 GCP-V).

8.7 Erneute Überprüfung der Nutzen-Risiko-Bewertung

Der Sponsor unterrichtet unverzüglich, aber spätestens **innerhalb von 15 Tagen** nach Bekanntwerden, die zuständige Ethikkommission sowie die zuständige Bundesoberbehörde (BfArM) über jeden Sachverhalt, der eine erneute Überprüfung der Nutzen-Risiko-Bewertung des Prüfpräparates erfordert. Hierzu gehören insbesondere:

- Einzelfallberichte von erwarteten schwerwiegenden Nebenwirkungen mit einem unerwarteten Ausgang
- Erhöhung der Häufigkeit erwarteter schwerwiegender Nebenwirkungen, die als klinisch relevant bewertet werden
- Verdachtsfälle schwerwiegender unerwarteter Nebenwirkungen, die entstanden, nachdem der Prüfungsteilnehmer die klinische Prüfung bereits beendet hat

- Ereignisse im Zusammenhang mit der Studiendurchführung oder der Entwicklung des Prüfpräparates, die möglicherweise die Sicherheit der Prüfungsteilnehmer beeinträchtigen können.

9 Statistik

9.1 Fallzahlplanung

Aufgrund fehlender Vordaten kann die Fallzahl für diese Pilotstudie nur auf Basis von Schätzwerten berechnet werden. Das Ziel der Pilotstudie ist es, Daten über den Effekt von Xenon zu gewinnen, mit denen für das Folgeprojekt eine valide Fallzahlberechnung durchgeführt werden kann.

Aus der Literatur und nach klinischer Erfahrung ist in der Kontrollgruppe mit einem maximalen Abfall der GFR um 30 %, entsprechend 54 ml/min/1,73 m² KOF bei einem nierengesunden Menschen (180 ml/min/1,73 m² KOF), zu rechnen. Ein um 10 % geringerer Abfall in der Xenon-Gruppe, entsprechend 36 ml/min/1,73 m² KOF bei einem nierengesunden Menschen, ist als klinisch relevanter Unterschied anzusehen. Bei einer angenommenen Streuung σ in der Größenordnung von ca. 80% des Mittelwertunterschiedes δ von 30 ml/min/1,73 m² KOF, einer Power von $1 - \beta = 80$ % und einem 2-seitigen Signifikanzniveau von $\alpha = 5$ % werden 23 Patienten pro Gruppe benötigt, um eine Signifikanz des Effekts zu sehen. Da wir aus Erfahrungen bei früheren Studien mit einer Drop-out Rate von 10 % rechnen, werden 2 Patienten pro Gruppe zusätzlich eingeschlossen, um dies zu kompensieren. Die Gesamt-Patientenanzahl in der Pilotstudie beträgt demnach 25 Patienten pro Gruppe. Mit diesen Fallzahlen können auch „positive Signale“ mit 2-seitigen p-Werten unter 20% für Effekte ab einer standardisierten Differenz von ca. $\delta / \sigma = 0{,}6$ mit einer Power von 80% generiert werden. Bei wesentlich kleineren Effekten erscheint eine Weiterverfolgung dieser Studienidee aus klinischen und ökonomischen Gründen wenig sinnvoll.

9.2 Patientenauswahl für den Einschluss in die Analyse

Alle randomisierten Patienten werden in die Analyse eingeschlossen.

9.3 Anwendung von statistische Tests

Alle gesammelten Daten werden deskriptiv entsprechend dem Studienprotokoll erfasst. Nach dem letzten Überprüfungsdurchgang auf Fehlerfreiheit der SPSS-

Datenmaske werden die Daten an die Biometrie (IMSIE Köln) übergeben. Durch den Vergleich der Werte in der Isofluranegruppe zu der Xenongruppe zu jedem erfassten Zeitpunkt, erfolgt die Analyse des primären Wirksamkeitskriteriums GFR durch den Einsatz einer Kovarianzanalyse.

Die Hauptzielvariable max. Veränderung der GFR-Werte wird gemäß 2.3.4 berechnet. Diese Hauptzielvariable wird primär mit einer Kovarianzanalyse mit dem Faktor Gruppe und der Kovariaten Ausgangswert über einen 2-seitigen Mann-Whitney-Test zum Niveau von 5% ausgewertet. Die primäre Auswertung wird nach dem Intention-to-Treat-Prinzip durchgeführt (fehlende Werte werden durch Imputationen, deren genauere Methoden in einem späteren Statistical Analysis Plan definiert werden, ersetzt, sodass konservative Abschätzungen des Effekts erreicht werden), die sekundäre nach dem per-protocol-Prinzip. Die Nebenzielvariablen werden analog mit üblichen 2-Stichproben-Tests deskriptiv bzw. explorativ ausgewertet.
Die deskriptive Auswertung für die Baseline- und Outcome-Variablen erfolgt mit Hilfe der üblichen Maßzahlen und Kenngrößen, Graphiken und Tests (p-Werte) in Abhängigkeit der Skalenniveaus der Merkmale.

9.4 Prozeduren zur Behandlung von fehlenden Daten

Jeglicher mögliche Aufwand wird betrieben, damit die Zahl von fehlenden Werten so gering wie nur möglich bleibt. Die ausgefüllten CRF`s werden vor der statistischen Analyse, durch den Studienarzt oder eine study nurse auf die Vollständigkeit und Korrektheit überprüft. Fehlende Daten werden gesichtet und falls möglich aus den Quelldaten des Patienten erhoben und in die CRF`s nachgetragen. Fehlende Daten, welche im Nachhinein nicht ermittelt werden können, werden entsprechend dem „Intention to Treat Prinzip“ hochgerechnet.

9.5 Auswertungsprozeduren bei Abweichungen vom ursprünglichem statistischen Plan

Jede Abweichung vom ursprünglichen statistischen Plan wird im Protokoll und im Endbericht beschrieben und begründet.

10 Dateneingabe und Datenmanagement

10.1 Dateneingabe

Die Datensammlung und die Pseudonymisierung werden von jeweiligen Prüfarzt und dessen Mitarbeitern durchgeführt. Nur diese Personen, der Monitor und der Auditor haben Zugang zu den Patientenakten. Die Pseudonymisierung wird über die Zentrumsnummer und eine laufende Nummer erfolgen. Der jeweilige Arzt muss gewährleisten, dass eine Identifikation einer bestimmten Person zu jeder Zeit über die Registrierungsliste möglich ist. Deshalb verfügt er über eine Identifikationsliste mit dem vollen Patientennamen. Falls es nötig sein sollte, einen Patienten aus Sicherheitsgründen zu identifizieren, dann unterliegen alle beteiligten Personen der Schweigepflicht. Der Prüfer ist verantwortlich für die korrekte und vollständige Dokumentation der Studiendaten.

10.2 Datenaufbewahrung

Die Studien-CRFs und die Daten der gescreenten, aber nicht in die Studie eingeschlossenen Patienten werden zusammen mit den vollständigen Studiendokumenten (Prüfplan, Ethikvotum, BfArM-Zustimmung u.a.) in einem für die Allgemeinheit unzugänglichen Sicherungsschrank in den Räumen der Klinik für Anästhesiologie für den gesetzlich vorgegebenen Aufbewahrungszeitraum (15 Jahre) aufbewahrt.

10.3 Datenmanagement

10.3.1 Zugang zu Quelldaten

Aufgrund gesetzlicher Regelungen zur Sicherung der Datenqualität und zur Überwachung der Studiendurchführung am Zentrum sind die Prüfärzte verpflichtet, autorisierten Dritten Einsicht in die Patientenakten (Quelldaten) zu gewährleisten. Dazu zählen Monitore, Auditoren, unabhängige ethische Komitees und Behörden. Diese Personen sind zur Verschwiegenheit verpflichtet.

10.3.2 Monitoring

Durch den Sponsor wird ein Monitor engagiert, der bei allen Studienpatienten das Vorhandensein der schriftlichen Einwilligung überprüft, sowie stichprobenartig die Übereinstimmung zwischen den Originaldaten und den CRFs überprüft. Zur

Durchführung des Monitorings ermöglichen die Prüfer den Zutritt zu den Räumlichkeiten der Studiendurchführung, inkl. Zugriff auf die Akten aller Studienteilnehmer zur Gewährleistung eines vollständigen Quelldatenvergleichs.
Die Daten werden aus den Papier-CRFs mittels Doppeleingabe durch zwei unabhängige studentische Hilfskräfte in jeweils eine Datenmaske übertragen. Der verantwortliche Biometriker überprüft die beiden Dateien mittels eines dafür programmierten Algorithmus auf Übereinstimmung. Gibt es Diskordanzen zwischen den beiden Dokumenten, wird der Monitor informiert, der den Abgleich mit dem Papier-CRF übernimmt. Alle fehlenden und unplausiblen Werte werden an den Prüfer zurückgefragt, der verpflichtet ist, diese zu beseitigen oder zu erklären. Die Änderung in den SPSS-Datenmasken führen dann die studentischen Hilfskräfte durch. Die Dateneingabe und Datenkorrekturen müssen mit Datum, Uhrzeit und Namen der bearbeitenden Person versehen werden. Des Weiteren kontrolliert der Monitor auch die Durchführung der Intervention.

10.4 Abschlussbericht

Im Anschluss an die Auswertung der Studienergebnisse, erstellt der Hauptprüfer einen kompletten Abschlussbericht entsprechend den ICH-GCP-Richtlinien, welcher dem Sponsor ausgehändigt wird und

11 Qualitätskontrolle und Qualitätssicherung

11.1 Qualitätskontrolle

Zur Sicherstellung einer korrekten, verlässlichen und übereinstimmenden Datenerhebung und zur Garantie des Gebrauches von Standardbedingungen und standardisierten Vorgehensweisen (SOP), wird diese Studie entsprechend den Anforderungen der ICH Guideline for Good Clinical Practice (GCP) E6 vom Juni 1996 und der CPMP/ICH/135/95 vom September 1997 und der geltenden gesetzlichen Bestimmungen durchgeführt.

11.2 Qualitätssicherung

Die folgenden Bestimmungen zur Qualitätskontrolle werden eingesetzt:

- Feedback Gespräche und Übungen, um den Prüfer für die Studien SOP`s vorzubereiten

- Monitoring der eingeschlossenen Patienten entsprechend den Kriterien für Sicherheit.
- Während des Studiendurchführungszeitraumes, darf der Sponsor jederzeit die Prüfungsstätte besichtigen und den Studienfortschritt, die Studiendurchführung, die Originaldaten und CRF`s begutachten.
- Abgleich der original Quelldaten und der CRF`s.
- Doppeleingabe der Papier-CRF`s durch zwei unabhängige studentische Hilfskräfte in SPSS-Datenmasken. Überprüfung dessen auf Übereinstimmung durch den verantwortlichen Biometriker und einen dafür programmierten Algorithmus.

12 Ethische und Administrative Aspekte

12.1 Erläuterungen zu den vorgesehenen Untersuchungen bei Versuchen an Menschen oder an vom Menschen entnommenem Material oder Tieren

Bei dieser Pilotstudie handelt es sich um eine klinische Prüfung eines zugelassenen Arzneimittels. Alle im Rahmen dieser Pilotstudie geplanten Untersuchungen am Menschen werden konform zum Arzneimittelgesetz (in der aktuellen Fassung von 2011) und nach der GCP-Verordnung (aktuelle Fassung 2006) auf Basis der Deklaration von Helsinki durchgeführt. Diese Durchführung beinhaltet vor Beginn der Studie ein zustimmendes Ethikvotum, die bundesoberbehördliche Erlaubnis zur Durchführung der Studie und die nicht-öffentliche Registrierung bei der European Medicines Agency (EMEA). Zusätzlich wird die Studie offen für alle Interessierten zugänglich bei clinicaltrials.gov registriert und damit einsehbar. Die Patienten werden mündlich und schriftlich über das Ziel, die Durchführung und den Nutzen der Studie inklusive dem individuellen persönlichem Nutzen und Schaden aufgeklärt. Vor dem Einschluss in die Studie muss dann schriftlich bekundet werden, dass der Einschluss in die Studie freiwillig und nach Aufklärung und Abwägen erfolgt. Zu jedem Zeitpunkt der Studie kann der Patient ohne Angabe von Gründen sein Einverständnis zurückziehen. Durch die Randomisierung ist eine Zufallszuteilung und damit Chancengleichheit auf beide Therapiegruppen gewährleistet. Durch Pseudonymisierung des Patienten und seiner Studiendaten unter einer Studiennummer wird der Datenschutz gegenüber Personen, die die Daten im

Regelfall nicht einsehen dürften (Biometriker, Datenmanager etc.), aufrechterhalten. Die genaue Auswahl und Behandlung der Patienten im Verlauf der Studie ist detailliert bereits unter Punkt 4 und 5 beschrieben. Die Operation und die Anästhesie für den Eingriff werden nach den gängigen klinischen Standards bzw. Leitlinien durchgeführt. Für die Studienanästhesie wird für beide Gruppen (Xenon und Kontrolle) wird eine SOP zur Durchführung festgelegt, die sich nur im Studienmedikament unterscheidet. Die Patienten werden während der Narkoseführung genauestens überwacht, im Rahmen der Studie werden Sicherheitsdaten wie Anästhesiedaten und Vitalparameter wie oben beschrieben in engen Abständen erfasst. Zu jeder Zeit ist das gesundheitliche Wohl des Patienten das oberste Gebot. Gibt es zu irgendeinem Zeitpunkt der Studiendurchführung einen Anhalt für einen drohenden oder eingetretenen Schaden für den Patienten, unabhängig ob studienassoziiert oder nicht, wird für den Patienten die Studiendurchführung beendet und eine adäquate medizinische Diagnostik und Therapie unverzüglich eingeleitet. Alle Patienten erhalten bei Beendigung (planmäßig oder individuell) eine gründliche Abschlussuntersuchung und werden bei auffälligem Ergebnis einer weiteren Diagnostik und Therapie zugeführt. Zusammenfassend gesagt, ist das Prüfpräparat Xenon ein sicheres und für Anästhesien in dem beschriebenen Rahmen bereits zugelassenes Medikament. Das Risiko einer Studienteilnahme übersteigt nicht das Risiko einer Allgemeinanästhesie für eine Nierenteilresektion. Aus dem Tumorpräparat der Niere wird wie bei der Standardversorgung eine Histopathologie im Paraffinschnitt und Einstufung des Tumorstadiums durchgeführt. Aus dem Paraffinblock werden zusätzliche Schnitte angefertigt, in denen ggf. im Rahmen der Pilotstudie mittels Immunhistochemie HIF 1a im gesunden Randgebiet des Tumors untersucht wird. Eine Aufbewahrung des Präparats über die Standardversorgung hinaus ist nicht geplant.

12.2 Zustimmung einer unabhängigen Ethikkommission

Die Zustimmung einer unabhängigen Ethikkommission wird vor der Konsultation der Bundesbehörde und vor dem Studienbeginn entsprechend dem GCP-V bei der:

Ethik-Kommission an der Medizinischen Fakultät der RWTH Aachen
Vorsitz: Prof. Dr. med. G. Schmalzing
Universitätsklinikum Aachen

Pauwelsstr. 30, D-52074 Aachen
Tel.: +49-241-8089963
Fax.: +49-241-8082012
Mail: ekaachen@ukaachen.de

12.3 Zustimmung der Bundesbehörde

Die Zustimmung der Bundesbehörde wird vor dem Studienbeginn entsprechend GCP-V eingeholt:

Bundesinstitut für Arzneimittel und Medizinprodukte BfArM
Kurt-Georg-Kiesinger-Allee 3, D-53175 Bonn
Tel.: +49 228 207-30
Fax: +49 228 207-5207
Mail: poststelle@bfarm.de

12.4 Änderung des Studienprotokolls

Zur Sicherstellung einer einwandfreien Datenauswertung ist eine Änderung der vereinbarten und im Prüfplan niedergelegten Prüfungsbedingungen nicht vorgesehen. In Ausnahmefällen sind jedoch Änderungen der Prüfungsbedingungen möglich. Diese erfolgen nur nach gegenseitiger Abstimmung zwischen dem Prüfer und dem Sponsor. Jede Änderung der im Prüfplan vorgesehenen Studienprozedur muss schriftlich, unter Angabe der jeweiligen Gründe, erfolgen und von allen Studienverantwortlichen unterschrieben werden. Die Änderungen gelten dann als Bestandteil des Prüfplans. Sofern erforderlich (bei z. B. Änderung der Medikationsdosis und/oder anderen bedeutsamen Veränderungen, die einen direkten Einfluss auf die Sicherheit der Studienteilnehmer erkennen lassen), ist die Zustimmung der zuständigen Ethikkommission und/oder Behörden sowie des Patienten zu den Prüfplanänderungen einzuholen, und das Amendment ist der Bundesbehörde (BfArM) vorzulegen.

12.5 Studienabschluss

Das Studienende wird entsprechend § 12 and 13 GCP-V durch den Sponsor an die Bundesbehörde innerhalb von 90 Tagen gemeldet.

12.6 Abschlussbericht

Ein Abschlussbericht wird entsprechend § 13 GCP-V durch den Sponsor an die Bundesbehörde innerhalb von einem Jahr gemeldet.

13 Dokumentation

13.1 Datenerhebung und Datenverwaltung

13.1.1 Quelldaten (Source Data)

Alle Daten aus den studienbezogenen Untersuchungen und Erhebungen werden direkt in studienspezifische Case Report Forms (CRF`s) eingetragen und werden folglich als Quelldaten erachtet. Zusätzliche Informationen aus nicht studienbezogenen Untersuchungen, bzw. Daten aus der Patientenakte wie EKG, Laborausdrucke, werden fotokopiert und auch als Quellinformationen unverzüglich in die CRF`s geheftet.

13.1.2 Case Report Forms (CRF`s)

Für jeden Studienteilnehmer werden zwei CRF`s erstellt, einer für die erhobenen Daten des Studienarztes 1 und ein CRF für die Daten des Studienarztes 2. Die CRF`s werden mit einem wasserunlöslichen Stift von Hand ausgefüllt. Die CRF`werden entsprechend dem Punkt 10.2 aufbewahrt. Sobald die Studienergebnisse ausgewertet wurden, erstellt der Hauptprüfer einen ausführlichen Studienbericht entsprechend den ICH-GCP Richtlinien und händigt diesem dem Sponsor aus.

13.2 Digitalisierung der Studiendaten

Die vollständigen CRF`s werden durch den Studienarzt oder eine Study Nurse auf Vollständigkeit und Korrektheit überprüft. Hieran schließt sich die Übertragung der Papier-CRF`s mittels Doppeleingabe durch zwei unabhängige studentische Hilfskräfte in SPSS-Datenmasken, um die Übereinstimmung der beiden Dateien mittels eines programmierten Algorithmus erneut zu überprüfen. Erst danach schließt sich die statistische Analyse an.

13.3 Archivierung der Studiendaten

Der Sponsor dieser Studie – Clinical Trials Center Aachen (CTC-A), Universitätsklinikum Aachen, Pauwelsstr. 30, D-52074 Aachen – ist verpflichtet entsprechend dem ICH Topic E6 (R1) Guideline for Good Clinical Practice (CPMP/ICH/135/95) alle wichtigen Dokumente, wie unter den Punkten 8.2 – 8.6 dargestellt, für 15 Jahre aufzubewahren. Der Abschlussbericht muss für 5 Jahre aufbewahrt werden. Dokumente aus einer vorzeitig beendeten Studie müssen 2 Jahre lang nach dem Studienabbruch aufgehoben werden.

Die oben aufgeführten wichtigen Dokumente, werden vom Hauptprüfer in einem für die Allgemeinheit unzugänglichen Sicherungsschrank in den Räumen der Klinik für Anästhesiologie für den gesetzlich vorgegebenen Aufbewahrungszeitraum (15 Jahre) aufbewahrt. Der Hauptprüfer hat auch dafür Sorge zu tragen, dass eine fälschliche oder vorzeitige Vernichtung dieser Dokumente verhindert wird.

14 Formales

Die klinische Prüfung wird in Übereinstimmung mit dem Prüfplan, dem deutschen Arzneimittelgesetz (AMG), den ICH GCP Richtlinien, der Deklaration von Helsinki und der Bekanntmachung von Grundsätzen für die ordnungsgemäße Durchführung der klinischen Prüfung von Arzneimitteln durchgeführt.

14.1 Finanzierung

Die Studie wird durch die Deutsche Forschungsgemeinschaft; DFG und durch die Klinik für Anästhesiologie (Universitätsklinikum Aachen, Direktor: Univ.-Prof. Dr. Rolf Rossaint) finanziert.

Die detaillierten, finanziellen Aspekte dieser Studie werden in separaten Verträgen festgehalten.

14.2 Studienversicherung

Gemäß den aktuellen, gesetzlichen Vorgaben (§ 40 Abs. 1b) bedarf es keiner gesonderte Versicherung der Studienteilnehmer.

Bei verschuldensabhängigen Zwischenfällen sind die Patienten über die Betriebhaftpflichtversicherung des Universitätsklinikum Aachen versichert.

15 Publikationsvereinbarung

Nach Auswertung der Daten durch den verantwortlichen Biometriker erfolgt die Entblindung. Es werden ein integrierter Studienbericht und ein Manuskript durch den Hauptprüfer und den Biometriker erstellt, das schlussendlich an ein peer-reviewed Journal zur Veröffentlichung eingeschickt wird.

Jährlich sowie nach Abschluss der Studie und Auswertung der Daten verfasst der Hauptprüfer einen Bericht für die DFG.

16 Signaturen

Dem Prüfplan stimmen zu:

VERTRETER DES SPONSORS

**Dipl.-Biol. Verena Deserno**<br>**Koordinierende Geschäftsführerin CTC-A**	Aachen,

PRÜFER DER KLINISCHEN PRÜFUNG

**Dr. Astrid Fahlenkamp**<br>**Klinik für Anästhesiologie**	Aachen,

STELLVERTRETER

**Prof. Dr. med. Mark Coburn**<br>**Klinik für Anästhesiologie,**	Aachen,

KLINIKDIREKTOR

**Univ.-Prof. Dr.med. Rolf Rossaint**<br>**Klinik für Anästhesiologie,**	Aachen,

STATISTIKER

**Prof. Dr. Walter Lehmacher**	Köln, 20.3.2013<br>W. Le[illegible]

17 Literaturverzeichnis

1. Bono AV, Lovisolo JA. Renal cell carcinoma--diagnosis and treatment: state of the art. Eur Urol. 1997;31 Suppl 1:47-55.
2. Hollingsworth JM, Miller DC, Daignault S, Hollenbeck BK. Rising incidence of small renal masses: a need to reassess treatment effect. J Natl Cancer Inst. 2006;98:1331-1334.
3. Jemal A, Siegel R, Xu J, Ward E. Cancer statistics, 2010. CA Cancer J Clin. 2010;60:277-300.
4. Kane CJ, Mallin K, Ritchey J, Cooperberg MR, Carroll PR. Renal cell cancer stage migration: analysis of the National Cancer Data Base. Cancer. 2008;113:78-83.
5. Ljungberg Cowan NC, Hanbury DC, Hora M, Kuczyk MA, Merseburger AS, Patard JJ, Mulders PF, Sinescu IC; European Association of Urology Guideline Group. EAU guidelines on renal cell carcinoma: the 2010 update. Eur Urol. 2010;58:398-406.
6. Han JS, Huang WC. Impact of Kidney Cancer Surgery on Oncologic and Kidney Functional Outcomes. Am J Kidney Dis. 2011;58:846-854.
7. Lane BR, Russo P, Uzzo RG, Hernandez AV, Boorjian SA, Thompson RH, Fergany AF, Love TE, Campbell SC. Comparison of cold and warm ischemia during partial nephrectomy in 660 solitary kidneys reveals predominant role of nonmodifiable factors in determining ultimate renal function. J Urol. 2011;185:421- 7.
8. Yossepowitch O, Eggener SE, Serio A, Huang WC, Snyder ME, Vickers AJ, Russo P. Temporary renal ischemia during nephron sparing surgery is associated with short-term but not long-term impairment in renal function. J Urol. 2006;176:1339-43; discussion 1343.
9. Simmons MN, Schreiber MJ, Gill IS. Surgical renal ischemia: a contemporary overview. J Urol. 2008;180:19-30.
10. Coburn M, Kunitz O, Baumert J-H, Hecker K, Haaf S, Zühlsdorf A, Beeker T, Rossaint R. Randomized controlled trial comparing hemodynamic and recovery effects of xenon. Br J Anaesth 2005;94:198-202.
11. Rossaint R, Reyle-Hahn M, Schulte Am Esch J, Scholz J, Scherpereel P, Vallet B, Giunta F, Del Turco M, Erdmann W, Tenbrinck R, Hammerle AF, Nagele P; Xenon Study Group. Multicenter randomized comparison of the efficacy and

safety of xenon and isoflurane in patients undergoing elective surgery. Anesthesiology. 2003;98:6-13.

12. Hobbs C, Thoresen M, Tucker A, Aquilina K, Chakkarapani E, Dingley J. Xenon and hypothermia combine additively, offering long-term functional and histopathologic neuroprotection after neonatal hypoxia/ischemia. Stroke 2008;39:1307-1313
13. Coburn M, Maze M, Franks NP. The neuroprotective effects of xenon and helium in an in vitro model of traumatic brain injury. Crit Care Med. 2008;36:588-95.
14. Hein M, Roehl AB, Baumert JH, Bleilevens C, Fischer S, Steendijk P, Rossaint R. Xenon and isoflurane improved biventricular function during right ventricular ischemia and reperfusion. Acta Anaesthesiol Scand. 2010;54:470-8.
15. Weber NC, Stursberg J, Wirthle NM, Toma O, Schlack W, Preckel B. Xenon preconditioning differently regulates p44/42 MAPK (ERK 1/2) and p46/54 MAPK (JNK 1/2 and 3) in vivo. Br J Anaesth. 2006;97:298-306.
16. Mio Y, Shim YH, Richards E, Bosnjak ZJ, Pagel PS, Bienengraeber M. Xenon preconditioning: the role of prosurvival signaling, mitochondrial permeability transition and bioenergetics in rats. Anesth Analg. 2009;108:858-66.
17. Schwiebert C, Huhn R, Heinen A, Weber NC, Hollmann MW, Schlack W, Preckel B. Postconditioning by xenon and hypothermia in the rat heart in vivo. Eur J Anaesthesiol. 2010;27:734-9.
18. Ma D, Lim T, Xu J, Tang H, Wan Y, Zhao H, Hossain M, Maxwell PH, Maze M. Xenon preconditioning protects against renal ischemic-reperfusion injury via HIF-1alpha activation. J Am Soc Nephrol. 2009;20:713-20.
19. Rizvi M, Jawad N, Li Y, Vizcaychipi MP, Maze M, Ma D. Effect of noble gases on oxygen and glucose deprived injury in human tubular kidney cells. Exp Biol Med (Maywood). 2010;235:886-91.
20. Irani Y, Pype JL, Martin AR, Chong CF, Daniel L, Gaudart J, Ibrahim Z, Magalon G, Lemaire M, Hardwigsen J. Noble gas (argon and xenon)-saturated cold storage solutions reduce ischemia-reperfusion injury in a rat model of renal transplantation. Nephron Extra. 2011;1: 272-82.
21. Stoppe C. Fahlenkamp AV, Rex S, Veeck NC, Gozdowsky SC, Schälte G, Autschbach R, Rossaint R, Coburn M: Feasibility and safety of xenon compared to sevoflurane anaesthesia in coronary surgical patients – a randomized controlled pilot study. In revision at Br J Anesth 2012.

22. Stoppe C, Werker T, Rossaint R, Dollo F, Lue H, Wonisch W, Menon A, Götzenich A, Bruells CS, Coburn M, Kopp R, Bucala R, Bernhagen J, Rex S. What ist he significance of perioperativ release of macrophage migration inhibitory factor in cardiac surgery? Antioxid Redox Signal. 2012 Nov 19.
23. Roggenbach J, Morath C. Postoperatives Nierenversagen. Der Nephrologe 02/2009; 4(2):118-127
24. Zhang L, Huang H, Cheng J, Liu J, Zhao H, Vizcaychipi MP, Ma D. Pre-treatment with isoflurane ameliorates renal ischemic-reperfusion injury in mice. Life Sci. 2011;88:1102-7.
25. Lee HT, Kim M, Kim J, Kim N, Emala CW. TGF-beta1 release by volatile anesthetics mediates protection against renal proximal tubule cell necrosis. Am J Nephrol. 2007;27:416-24.
26. Lee HT, Ota-Setlik A, Fu Y, Nasr SH, Emala CW. Differential protective effects of volatile anesthetics against renal ischemia-reperfusion injury in vivo. Anesthesiology. 2004;101:1313-24.
27. Bito H, Ikeuchi Y, Ikeda K. Effects of low-flow sevoflurane anesthesia on renal function: comparison with high-flow sevoflurane anesthesia and low-flow isoflurane anesthesia. Anesthesiology. 1997;86:1231-7.
28. Teixeira S, Costa G, Costa F, da Silva Viana J, Mota A. Sevoflurane versus isoflurane: does it matter in renal transplantation? Transplant Proc. 2007;39:2486-8.
29. Ko JS, Kim G, Shin YH, Gwak MS, Kim GS, Kwon CH, Joh JW. The effects of desflurane and isoflurane on hepatic and renal functions after right hepatectomy in living donors. Transplant Proc. 2012;44:442-4.
30. Story DA, Poustie S, Liu G, McNicol PL. Changes in plasma creatinine concentration after cardiac anesthesia with isoflurane, propofol, or sevoflurane: a randomized clinical trial. Anesthesiology. 2001;95:842-8.
31. Coll E, Botey A, Alvarez L, Poch E, Quintó L, Saurina A, Vera M, Piera C, Darnell A. Serum cystatin C as a new marker for noninvasive estimation of glomerular filtration rate and as a marker for early renal impairment. Am J Kidney Dis. 2000;36:29-34.
32. Larsson A, Malm J, Grubb A, Hansson L-O. Calculation of glomerular filtration rate expressed in mL/min from plasma cystatin C values in mg/L. Scand J Clin Lab Invest 2004; 64: 25–30.

33. Herget-Rosenthal S, Pietruck F, Volbracht L, Philipp T, Kribben A. Serum cystatin C--a superior marker of rapidly reduced glomerular filtration after uninephrectomy in kidney donors compared to creatinine. Clin Nephrol. 2005;64:41-6.
34. Mehta RL, Kellum JA, Shah SV, Molitoris BA, Ronco C, Warnock DG, Levin A, Acute Kidney Injury Network. Acute Kidney Injury Netw
35. Ricci Z, Cruz D, Ronco C. The RIFLE criteria and mortality in acute kidney injury: A systematic review. Kidney Int 2008; 73: 538-546.
