# Supplementary material for: Renal function following xenon anesthesia for partial nephrectomy—An explorative analysis of a randomized controlled study
Source: PLoS One. 2017 Jul 18;12(7):e0181022. doi: 10.1371/journal.pone.0181022 (PMC5515428; doi:10.1371/journal.pone.0181022)
Supplement: S2 Appendix — (DOCX) [file pone.0181022.s003.docx]

**S2 Appendix. English translation of the German study protocol-**

**Background and Rationale**

**1.1 Background**

Renal cell carcinoma (RCC) is the third most common type of urological cancer in in Europe and the USA, with an increasing incidence due to incidental detection (with imaging techniques) of small mass tumors staged T1 [1-4]. A partial nephrectomy (PN) with preservation of nephrons is recommended in early-stage RCCs, under certain circumstances also in with stage T2 [5]. Notwithstanding the nephron-sparing effect of PN compared to radical nephrectomy, postoperative complications are common. This is due to the surgery-related ischemia during hilar clamping and indirect ischemia and reperfusion injury (IRI) involving tumor manipulation and resection itself [6]. Particularly impairment of renal function is common with a clinically significant glomerular filtration rate (GFR) decrease of 16-30% [6-8]. Beside limitations of clamping- and manipulation time, the use of cold ischemia for durations of more than 30 minutes ischemia, administration of mannitol, adequate hydration, avoidance of hypotension and blood loss, a postoperative GFR decline cannot fully be prevented [6-8]. After PN the GFR shows an initial nadir [8], followed by recovery to new reduced baseline levels. Evidence from clinical studies is limited [9], particularly in regard to the early postoperative maximum decrease of the GFR.

A therapeutic approach enhancing kidney-resilience to IRI would be of outstanding clinical relevance.

**1.2 Rationale**

The noble gas xenon (Xe) is approved since 2005 in Germany and 2007 in Europe for clinical routine use as an inhalational anesthetic. It is used for balanced anesthesia together with opioids for adult American Society of Anesthesiologists (ASA) ≤III patients. Different experimental *in vitro* and *in vivo* models were able to demonstrate the neuroprotective and cardioprotective effects induced by Xe treatment [12-15]. Both pre- and post conditioning protocols were effective [16-17]. Nephroprotection by Xe was recently shown in an *in vivo* IRI model and *in vitro* model of glucose deprived injury in human tubular kidney cells [18-19]. Both studies found an increased expression of hypoxia inducible factor-1α (HIF-1α) in the Xe treated cells or kidneys of the animals [18-19]. The nephroprotective effect was abolished by inhibition of HIF-1α activity [18]. Further nephroprotective effects showed Pype et al. Xe-saturated cold storage solutions reduced the ischemia-reperfusion injury after 6 hours of cold ischemia in a rat model of renal transplantation [20]. Animals with Xe-treated kidneys showed better creatinine-clearance after 7 and 14 days and decreased excretion of albumin in urine, which shows the tubular injury [20]. Clinical data from our study group revealed a better renal function in patients undergoing cardiac surgery after Xe anesthesia [21]. Furthermore, we could show that IRI on cardiac surgery patients leads to an enhanced release of macrophage migration- inhibiting factor (MIF) in serum. MIF was associated with an enhanced antioxidative capacity and less postoperative renal failure [22]. Therefore the influence of Xe compared to isoflurane on MIF should be examined in the present study. Beside the experimental organ protective effects, Xe is an effective anesthetic with favorable hemodynamic properties with blood pressure stability and low heart rates [10-11]. Other anesthetics like isoflurane, desflurane, sevoflurane and propofol usually show low blood pressures; with the need of catecholamines during anesthesia. Both low mean arterial pressures as well as the vasoconstrictive drugs like norepinephrine have additional negative influence on the renal function. This could lead to a beneficial risk-benefit ratio for Xe.

Xe is a extremely rare and natural gas, which is linked to higher costs. But a postoperative renal insufficiency is often associated with a longer hospital stay, more costs and higher mortality [23]. Reduction of the postoperative renal insufficiency by Xe, via organ protection or additional hemodynamic stability, could counterbalance the higher costs for Xe.

**1.3 Hypotheses**

The aim of this pilot-study is to compare the GFR decrease after Xe compared to standard anesthesia in patients undergoing PN. GFR should be measured daily until postoperative day 7. GFR and creatinine in serum will be assessed after 3-6 months to determine the longer-term renal function. Data from the control group will be compared to the Xe group. We aim to test our hypothesis, that Xe is more favorable for patients undergoing surgeries with temporarily reduced renal perfusion or clamping. Hereby Xe will induce an attenuated GFR decrease, either by hemodynamic stability before and after renal manipulation or by an unknown nephroprotective effect. Organ protection by Xe is often induced by pre-conditioning, which also protects renal tissue during ischemia. Novelty of our hypothesis is that the impact of anesthesia for nephroprotection during renal surgery was never investigated before. As described before, Xe anesthesia has a great potential to induce clinical significant nephroprotection. Randomized controlled trials are lacking with regard to this issue. We have chosen Iso as the comparative, as it was shown to have the strongest nephroprotective effect against IRI [24-25], compared to sevoflurane or desflurane [26]. Furthermore, Iso does not exhibit potentially nephrotoxic effects like sevoflurane [27] and it was not inferior in clinical studies examining postoperative renal function after major surgery and renal transplantation compared to sevoflurane, desflurane or propofol anesthesia [28-30].

**2 Objectives**

**2.1 Primary objective**

The aim of this project is to analyze a potential nephroprotection by Xe after PN in a randomized controlled pilot-study, by evaluation of the early postoperative GFR decline, compared to isoflurane anesthesia. If we could show a nephroprotective effect, we aim to perform a multicenter randomized controlled trial with a large sample size. The long-term goal is to develop a preventive strategy for attenuation of renal insufficiencies after renal-damaging surgeries (PN, renal transplantation). This could reduce short-term and long-term consequences (short-term dialysis, chronic renal insufficiencies, cardiovascular risk) for the patient and healthcare.

**2.2 Primary endpoint**

The primary endpoint of this study was to determine the maximum GFR decrease between the preoperative value and the lowest value within the first seven postoperative days after PN. GFR was calculated by a combined formula incorporating serum creatinine and serum cystatin C [31-33]. The maximum GFR decrease is calculated as the difference between the baseline GFR minus the lowest GFR within the first seven postoperative days.

**2.3 Secondary objectives/ secondary endpoints**

The following data will be assessed as secondary endpoints: Duration of direct manipulation-/ ischemia time during renal tumor resection; duration of anesthetic exposition before and after tumor resection; intraoperative blood loss, tumor size and histology; optional assessment of HIF 1 activation in healthy surrounding tissue; GFR time-course, determined by cystatin C assessment in serum; creatinine time-course within the first 7 days after PN; optional assessment of KIM-1 or NGAL in urine before and after tumor resection; urine output during surgery and on postoperative day 1-3; acute kidney injury with increasing AKIN-classification is already within 48h associated with increased mortality. This association was verified in several studies. A recent meta-analysis showed a doubled risk for mortality (RR= 2.4) for patients with AKIN I compared to patients without. Furthermore, it is increasing continuously from AKIN II to AKIN III (RR = 4.2 and RR = 6.4, respectively) and further on [35].

Macrophage migration-inhibiting factor (MIF) in serum will be assessed preoperatively, 5 minutes after termination of the tumor ground-treatment, directly postoperative in the PACU and after 24h. Additionally we will assess demographic data like age, height, BMI and anesthesia-related data (anesthetic-concentrations and amount of opioids and inspiratory oxygen-concentration) in this pilot-study. These data will be used to check the comparability of the two groups. According to GCP, safety data like vital patient data (oxygen saturation, blood pressure, heart rate) during anesthesia, laboratory data (blood cell count, coagulation data, urea, transaminases, creatine kinase) after surgery and all adverse events (AEs) und serious adverse events (SAEs) will be assessed in addition. After 3-6 months creatinine in serum and GFR should be determined for longer-term renal function course. This will be achieved by contacting of the familiar physician or urologist during the routine post-surgical care.

**3 Methods**

**3.1 Study design**

The present pilot-study will be a mono-center, prospective randomized controlled phase III study, with a patient and outcome assessor blinded design. It will be conducted in the University Hospital RWTH Aachen

• Group 1: Xenon-group: 60% Xenon/ 40% oxygen inspiratory.

• Group 2: Control-group: 1,2 Vol.% end-expiratory Isoflurane/ 40% oxygen/ air-mix.

All other drugs, which will be used for the standardized anesthesia, will be specified in a standard operating procedure form before patient recruitment.

**3.2 Randomization**

The randomization sequence will be generated computer-based by an independent biometrician. Patients will be randomly allocated according to a computer-based algorithm into the two groups Xe or control. Allocation concealment will be achieved by sequentially numbered, sealed opaque numbered envelopes, which will be kept safe until randomization of the single patients. Patients will be enrolled and pseudonymized with sequential increasing numbers by an investigator after obtaining written informed consent. All data will be collected under this pseudonymized number and only the biometrician and the monitor will have access to the randomization sequence, which includes the allocated treatment (Xe or control). The patients, the outcome-assessing investigator and the surgeon will be blinded (the ventilator will be the same for all patients to prevent accidental un-blinding). Only the second investigator, who performs the anesthesia and the intraoperative data assessing study-nurse, cannot be blinded.

**3.3 Storage of the randomization-codes and un-blinding**

**3.3.1 Premature un-blinding**

During study conduction we will assure complete blinding. The principle investigator or sub-investigator can reverse blinding for patient protection in emergency cases. This un-blinding of the randomization number is restricted to cases of AE or serious adverse events (SAE) with clinical need. Premature un-blinding has to be documented in the patient file and the sponsor will be informed immediately. Emergency drugs and therapy will be administrated according to the medical evidence and usual clinical practice.

**3.3.2 Regular un-blinding**

Regular un-blinding will occur after termination of the study and database lock.

**3.4 Personnel and technical requirements**

**3.4.1 Agreements with researchers with regard to this study**

An agreement was established with the Department of Urology, director of the Department named Univ.-Prof. Dr. Axel Heidenreich, and the assistant professor Dr. med. David Pfister. The Department of Urology will perform PN and look after the patients. Furthermore, there is an research agreement with the Institute of Pathology (director: Univ.- Prof. Dr. med. Ruth Knüchel-Clarke). The Institute of Pathology will routinely determinate the size and histology of the resected tissue. Optionally for this study they will determine HIF 1α in the healthy surrounding tissue by immunohistochemistry and KIM-1 or NGAL in urine by ELISA, in addition.

A further cooperation is established with the Institute of Medical Statistics, Informatics and Epidemiology (IMSIE), university of cologne (director Univ.-Prof. Dr. rer. nat. Walter Lehmacher). This Institute will provide the responsible biostatistician for the randomization, the GCP-conform data verification, -storage, and analysis.

**3.4.2 Technical equipment**

All technical devices, which are required for this project, are present in the involved institutes and departments. In particular the specific ventilator for Xe anesthesia and control anesthesia conduction (Felix Dual, Taema, ALMS, France) is available in the anesthesia department. Further standardized anesthesia equipment as well as the equipment for surgery and histopathology analysis is available in the participating departments. A certified local laboratory for determination of the laboratory variables is present in the University Hospital. There are plenty of computers for data entering, analysis and manuscript writing available in the Department of Anesthesia and the Medical Statistics, Informatics and Epidemiology Institute. The required software (SPSS and randomization software) is also available.

**4 Study population**

**4.1 Study- and intervention duration**

The study duration will last for each patient from the moment of the written informed consent until the discharge date or maximum the seventh postoperative day. Depending on the surgery time-point this will be 8-10 days. After 3-6 months we will assess the creatinine in serum and GFR, if measured by the family physician or urologist. The intervention duration will correspond to the anesthesia duration for the respective surgery, which will commonly be around 3 hours. The total recruitment period (first patient in to last patient out) is planned for sixteen months.

**4.2 Inclusion criteria**

All inclusion criteria have to be fulfilled before patient enrolment into the study.

1. Male or female patients with ≥18 years of age
2. Suspected renal carcinoma limited to one kidney
3. Planned for a partial nephrectomy (PN)
4. Written informed consent, after detailed information by the investigator

**4.2 Exclusion criteria**

1. Patients with a chronic kidney disease (CKD), and a pre-operative glomerular filtration rate (GFR) < 60 ml min^-1^ 1.73^-1^ m^-2^
2. American Society of Anesthesiologists (ASA) status > III
3. Known contraindication and hypersensitivity to propofol, sufentanil, Xe, Iso or rocuronium
4. Severe pre-existing cardiac disease (New York Heart Association-Classification (NYHA)<III), acute coronary syndrome during the past 24 hours, hemodynamic instability or requirement of catecholamines
5. Severe respiratory disease (Forced expiratory volume in one second/ forced vital capacity (FeV1/FVC)<70% and FeV1<30%), severe respiratory insufficiency with a partial pressure of oxygen in arterial blood (PaO_2_)<60mmHg, or home oxygen therapy
6. Severe neurological disease
7. Increased intracranial pressure
8. Risk for malignant hyperthermia
9. Pregnant or breast-feeding woman
10. Legally incompetent patients
11. Refusal of consent
12. Concurrent participation in other interventional studies in the last 30 days
13. Language and communication difficulties during study information
14. People who are institutionalized by court or administrative order
15. Subjects with dependent/ employment relationship to the sponsor or the investigator

**4.4 Patient exclusion criteria and procedure**

**4.4.1 Reasons for premature study-exclusion and termination of the investigational product administration**

- Patient safety has the first priority. A patient will be excluded before intervention, if an exclusion criterion appears after patient inclusion- but before intervention- (e.g. change of the surgical technique, acute patient deterioration, etc.). Patient data will receive a screening number and will be stored in a locker.
- A retrospectively noticed deviation from the inclusion or exclusion criteria (after the beginning of the intervention) will induce a study drop out. Depending on the time-point, the patient will receive a standard anesthesia and the data management will excluded the patient.
- Consent withdrawal: Patients can withdraw whenever they want. All collected data until the withdrawal time-point as well as the randomization group have to be filed. The patient has to be informed, that already collected data will be used further on.
- Severe adverse events (SAE) and suspected severe adverse reactions (SUSAR), which prohibit further study conduction.
- Adverse events (AE) and adverse reactions (AR), which seem to be hazardous for the physical and psychological health of the patients, as evaluated by the principle investigator.
- Technical difficulties and required modifications of the investigational product administration device.

**4.4.2 Time-point of patient drop out/ cessation of study treatment**

Each patient may be excluded from this study at any time. If any of the above mentioned condition occurs, the patient will be immediately excluded. If this occurs during the administration of the study-treatment, this will be terminated and replaced by a standard anesthetic. The patient will be treated according to his medical need.

**4.4.3 Documentation of the time-point and kind of patient exclusion**

All data until exclusion from the study will be collected and documented. The reason and the exclusion time-point will be documented on a special CRF sheet. The investigator is obliged to name the main reason for exclusion. The sponsor has to be notified within <24 hours in case of SAE/ SUSAR. The sponsor will inform the regulatory body and the ethic committee about the SAE/ SUSAR if necessary.

**4.4.4 Replacement of excluded patients**

After randomization excluded patients will be replaced.

**4.4.5 Follow-up of excluded patients**

Excluded patients from the study remain patients of the University Hospital Aachen and will be treated according to the medical evidence and clinical practice by the attending physicians. The Department of Anesthesiology is 24 hours available by the patient, if the patient is transferred to a special institution. The contact information will be provided to the patient.

It will be recommended to each patient, who withdraws his study-participation, to participate on a final examination.

**5 Individual course of study**

**5.1 Verification of the inclusion- and exclusion criteria**

Verification of the inclusion- and exclusion criteria of potential study-patients (screening) will be conducted according to a predefined checklist: All patients meeting the inclusion criteria: Suspected renal carcinoma limited to one kidney, planned PN and age of ≥18 years will already be informed by the colleagues of the Urology Department. An investigator will visit them if they are interested in study participation. The investigator will inform the patients about the individual risk and benefit of this study and evaluate the exclusion criteria. The patients will receive a patient information and ample of time for their decision to participate in this study. A screening log will be kept, where all eligible patients have to be documented. The enrolled patients will additionally be documented in this screening log. If a patient is not enrolled in this study, then at least 1 exclusion criterion has to be documented. Within the verification of the inclusion / exclusion criteria, the investigator has to document if the patient has participated in another clinical trial within the prior 30 days. The patient will be informed that he is not allowed to participate in further clinical trials, as this bears unpredictable risks for the patient.

**5.2 Patient information**

Each potentially eligible patient has to be informed about the study aims, the voluntariness of participation, and the possibility of withdrawal without reasons, the pseudonymized utilization and publication of the results, and the risks and possible complications. The patients will be informed verbally by the investigator and written by the patient information. After provision of ample time, the patient will be requested to sign and date two informed consent forms. One form will be provided together with the patient information to the patient. The other form will be filed in the investigator site file.

**5.3 Visits**

All patients will undergo a maximum of 11 visits. Visits 0 and 2-10 will be performed in a blinded manner by investigator I. Only investigator II and the study nurse will perform the intraoperative visit un-blinded, due to the special kind of Xe anesthesia administration.

**5.3.1 Visit 0 (Baseline visit)**

Investigator I will perform the baseline visit, after written informed consent. This visit includes the patient medical history and clinical investigation for detection of exclusion criteria. He will assess demographic data (gender, height, weight, age, ASA-status and other), vital function (heart rate, blood pressure, oxygen saturation with air), the central laboratory data (blood cell count, coagulation variables, urea in serum, transaminases, creatine kinases) and the preoperative GFR (estimated by cystatin C in serum) and the creatinine in serum. Additionally, a serum (for MIF determination) and urine sample for additional analyses will be obtained. Data will be documented in a paper-based case report form (CRF). Patients will not be enrolled; if there are exclusion criteria, but they will receive a screening number kept in a file in a cabinet. If there are no exclusion criteria, they will receive a study patient number. A treatment is assigned to each study number already before study start and concealed in an opaque sealed envelope.

**5.3.2 Visit 1 (Surgery day)**

Investigator II will open the randomization envelope shortly before anesthesia induction. Only the respective intraoperative investigator and the study nurse, who collects the intraoperative data, will have access to this treatment allocation. After application of the standard monitoring, the patients will receive a thoracic epidural catheter against postoperative pain.

Anesthesia induction: Both groups ( Xe and control) will receive propofol 1.5-2 mg kg^-1^ and sufentanil 0.2 µg kg^-1^; muscle relaxation will be induced by 0.6 mg kg^-1^ rocuronium.

Maintenance of anesthesia:

Patients in the Xe group will receive general anesthesia with 60% inspiratory Xe/ 40% O_2_, patients in the control group will receive 1.2 % isoflurane end-expiratory in mixed air with 40% O_2_; both groups will receive titrated sufentanil according to the clinical need.

Surgery:

- Mean arterial pressure (MAD) < 65mmHg; Prevention of additional impairment of renal function, by insufficient perfusion. Therefore, the MAD has to be kept continuously > 65mmHg, if necessary with titrated norepinephrine.
- Surgery will be performed according to the SOP of the Department of Urology.
- Assessment of the anesthesia- and safety data every 5 minutes, urine output every hour and the total urine output at the end of anesthesia.
- Incision-suture time, intraoperative blood loss, warm ischemia time corresponding to the tumor resection time and the anesthetic exposition until the beginning of the tumor resection time and after the end of this time (defined by the termination of the tumor ground-treatment), will be assessed in collaboration with the surgeons.
- Resected tumor tissue will be transferred to the Institute of Pathology, where the crop margins will be analyzed rapidly with regard to tumor-freedom. The tumor size, histology by immunochemistry, and optionally the activation of HIF 1α in the in the healthy surrounding tissue will be assessed in addition.
- 5 minutes after the end of ischemia time = termination of the tumor ground-treatment, we will assess macrophage migration-inhibiting factor (MIF) in serum.

Post-anesthesia-care unit (PACU):

- Blood pressure and heart rate will be assessed every 15 minutes and the pulse oxymetry will be measured continuously.
- A further serum sample for MIF measurement will be drawn in PACU

Investigator II will document all assessed data in the paper-based CRF of the respective patient. Follow-up during the hospital stay will last a maximum of seven postoperative days. Urine output will be assessed every 6 hours after surgery via urine catheter. The total urine output will be assessed in the second postoperative day. Urine samples will be collected on the first and second, as well as the seventh postoperative day for optional additional analyses (KIM-1 or NGAL by ELISA). Blood samples will be drawn each morning from the first until the discharge day or maximum 7^th^ postoperative day. Furthermore, the investigator will measure vital function (blood pressure, heart rate, oxygen saturation) daily. Cystatin C will be determined from the blood samples and the GFR will be calculated using cystatin C. Furthermore, the secondary blood outcome variables (creatinine, blood cell count, coagulation, urea in serum, transaminases and creatine kinase) will be determined in the blood samples. A further blood sample will be drawn on the first postoperative day for MIF determination in serum. The investigator will value and document laboratory variables and the amount of urine output in the respective CRF. In addition, the investigator will determine an acute renal failure according to the AKIN criteria [34] on the first and second postoperative day. In-hospital study participation is terminated after physical examination on the discharge day or maximum the 7^th^ postoperative day. We will try to obtain the GFR and creatinine value after 3-6 months postoperative by contacting of the family physician or attending urologist.

**5.3.3 Visit 2 (Surgery day, postoperative)**

- The urine output will be assessed every 6 hours after surgery via urine catheter.
- Furthermore, we will assess and document AEs and SAEs.

**5.3.4 Visit 3 (1^st^ postoperative day)**

- The urine output will be assessed every 6 hours after surgery via urine catheter until the second postoperative day.
- Investigator I will measure the patients` vital data (blood pressure, heart rate, oxygen saturation) and draw blood samples in the morning. We will need the following laboratory data: Cystatin C will be determined from the blood samples for calculation of GFR using cystatin C. Furthermore, the secondary blood outcome variables (creatinine, blood cell count, coagulation, urea in serum, transaminases and creatine kinase) will be determined in the blood samples.
- The last blood sample for MIF determination in serum will be drawn in addition.
- One urine sample will be drawn for optional KIM-1 or NGAL determination.
- The investigator will value and document laboratory variables and the amount of urine output in the respective CRF. The investigator will determine an acute renal failure according to the AKIN criteria [34] on the first and second postoperative day.
- Evaluation and documentation of AEs and SAEs.

**5.3.4 Visit 4-8 (2^nd^-6^th^ postoperative day)**

- Investigator I will measure the patients` vital data (blood pressure, heart rate, oxygen saturation) and draw blood samples in the morning.
- The total urine output will be measured on the 2^nd^ postoperative day
- One urine sample will be drawn.
- Laboratory data determination, please refer to 5.3.4
- The investigator will value and document laboratory variables and the amount of urine output in the respective CRF.
- The investigator will determine an acute renal failure according to the AKIN criteria [34] on second postoperative day.
- Evaluation and documentation of AEs and SAEs.

**5.3.6 Visit 9 (7^th^ postoperative day or discharge day/ end of study, respectively)**

- The investigator I will perform physical examination, measurement of the patients` vital data, blood sample drawing (laboratory variables, see 5.3.4). The in-hospital study participation ends maximum at the 7^th^ postoperative day.
- Evaluation and documentation of AEs and SAEs.

**5.3.7 Visit 10 (3-6 months postoperative)**

- Contacting of the family physician or attending urologist, to obtain the GFR and creatinine value, which was probably measured during the clinical postoperative routine.

**6 Investigational products**

**6.1 Name**

- Xenon 100% (v/v)
- Isoflurane

**6.2 Administration**

Both Xe anesthesia as well as control anesthesia will be administrated by a special closed circuit (Felix Dual, Taema, ALMS, France).

**6.3 Packaging, labeling and storage of the investigational drugs**

Medical Xe (LenoXe®, 100% v/v, Air Liquide) will be provided and labeled according to the European regulations in steel cylinders. It will be stored at room temperature, protected from light and in a cool place. Isoflurane (250ml, Abbott) will be provided in specific bottles and stored at 4 degrees Celsius in a light protected box. It will be vaporized by specific refillable vapors.

**6.4 Responsibility of investigational drug handling**

Xe (LenoXe®) will be supplied by Air Liquide Medical GmbH (Hans- Günther-Sohl-Str.5, 40235 Düsseldorf, Germany). Isoflurane (Forene®) will be supplied by the Abbott company. Both will be ordered by the central pharmacy (University Hospital Aachen; Director Dr. rer. nat. Albrecht Eisert; Steinbergweg 20; 52074 Aachen, Germany). Department of Anesthesia, University Hospital Aachen will hold the responsibility for correct handling of the study drugs in accordance with the DGAI (German Society for Anaesthesiology and Intensive Medicine).

**6.5 Criteria for premature study termination due to the used study drug**

All used study drugs are drugs, which are used daily for anesthesia conduction. They are approved since several years in Germany, and were validated in many studies. They are safely used in the daily anesthesia conduction. Therefore, we do not expect any reasons for premature study termination due to the study drugs.

**6.6 Names of the other study drugs**

- Propofol
- Sufentanil
- Rocuronium
- Oxygen
- Medical air

**6.7 Responsibility for the other drugs**

All other drugs will be ordered and provided by the central pharmacy (University Hospital Aachen; Director Dr. rer. nat. Albrecht Eisert; Steinbergweg 20; 52074 Aachen, Germany). Department of Anesthesia, University Hospital Aachen will hold the responsibility for correct handling of the study drugs in accordance with the DGAI (German Society for Anaesthesiology and Intensive Medicine).

**6.8 Concomitant therapy**

Concomitant therapy for comorbidities of the patient will be taken according to the suggestion of the investigator. There are no restrictions for concomitant therapies during the study.

**6.9 Prohibited drugs**

Other than the designated volatile anesthetics should not be used during visit 1 (general anesthesia).

**7 Outcomes**

**7.1 Outcome variables**

**7.1.1 Selection criteria and demographic data**

Following demographic data should be assessed:

- Birthdate
- Gender
- Height and weight
- Medical history, comorbidities and the respective medication, surgical history, smoking, alcohol use and/ or drug use, allergies
- ASA (America Society of Anesthesiologists) physical status
- Admission time-point
- Planned surgery time-point

**7.1.2 Primary outcomes**

The early postoperative renal function after Xe anesthesia compared to isoflurane anesthesia during PN, assessed by:

- GFR determination by cystatin C in serum, measured preoperatively and on the first 7 postoperative days. The maximum GFR decrease will be calculated as the difference between the preoperative baseline GFR and the nadir GFR within the first 7 postoperative days.

**7.1.3 Secondary outcomes**

The following secondary outcomes will be assessed in addition for the comparison of Xe anesthesia to isoflurane anesthesia:

- Measurement of the anesthesia and safety variables every 5 minutes and the determination of urine output at the end of surgery.
- Acute kidney failure determination according to the AKIN criteria, by measurement of the urine output every 6 hours until the second postoperative day, total urine output on the second postoperative day and determination of creatinine within the first 7 postoperative days or if earlier the day of discharge, respectively.
- Daily assessment of the safety data (creatinine, blood cell count, coagulation, urea in serum, transaminases and creatine kinase) until the 7^th^ postoperative day or if earlier the discharge day, respectively.
- Patients` vital data (blood pressure, heart rate, oxygen saturation) until the 7^th^ postoperative day or the discharge day (if earlier), respectively.
- Histological analysis with immunohistochemistry in paraffin sections and optional determination of HIF-1α in the tumor-free surrounding tissue via immunofluorescence staining.
- Determination of migration-inhibiting factor  (MIF) in serum.
- Determination of KIM-1 or NGAL in urine.
- Assessment of intraoperative blood loss.
- Assessment the anesthesia time until the beginning of the surgical ischemia (tumor resection time) and after the end of this surgical ischemia (defined by the termination of the tumor ground-treatment), until cessation of anesthesia. This will be assessed in collaboration of the investigator and the surgeons.
- Assessment of GFR and creatinine in serum after 3-6 months postoperative.

**7.2 Outcome measures**

Investigator I will assess inclusion criteria and demographic data during visit 0. Intraoperative data will be assessed, documented and analyzed by investigator II during visit 1. Blinded investigator I will assess the remaining secondary outcomes postoperatively until the 7th postoperative day or discharge day (if earlier), respectively. We will maintain two case report forms (CRFs) with all patient data, to assure blinding procedure. One CRF will include visit 0, 2-10, the other one for visit 1. All data outside of range will be validated by the investigator and marked as clinically significant (cs) or not clinically significant (ncs). Each clinically significant value will be considered as an AE and documented in the adverse event section.

**8 Safety analyses**

**8.1 Safety measures during general anesthesia for partial nephrectomy**

**8.1.1 Administration of the study treatment**

Concentration and dosage of the study drug will be assessed for each patient:

- Measurement of the inspiratory concentration and duration of the Xe- and isoflurane-exposition.
- Determination of the exposure duration with the study drugs until the beginning of the direct tumor resection time and after the end of this, until cessation of anesthesia.
- Total amount of Xe consumption (in liter).
- General anesthesia induction dose of propofol.
- Total amount of sufentanil during the whole anesthesia.
- Total amount of rocuronium during the whole anesthesia.

**8.1.2 Clinical safety variables**

- Systolic and diastolic blood pressure and heart rate will be assessed and documented every 5 minutes from entering the operating room until discharge from the operating room and every 15 minutes in the PACU.
- ECG will be measured continuously in the operating room and PACU. Investigator II will register clinically significant ECG deviations. They will be entered in the respective adverse event section, including the severity and treatment of the AE.
- Oxygen saturation will be assessed by pulse oxymetry continuously in the operating room and PACU. It will be documented every 15 minutes.
- Investigator II will assess the respiratory variables by Felix Dual® every 15 minutes during anesthesia (FiO_2_, saturation, FeO_2_, CO_2_et, Respiratory minute volume, respiratory rate, airway pressures).
- Cuff pressure will be checked every 5 minutes during ventilation and adjusted to 25-30 cmH_2_O.
- Urine output and blood loss will be measured every hour and at the end of surgery.
- Incision-suture time, warm ischemia time corresponding to the direct tumor resection time until termination of the tumor ground-treatment, will be assessed in collaboration with the surgeons.
- Occurrence of AEs and SAEs will be assessed GCP-conform during each visit and documented in the respective section of the CRF.

**8.2  Safety measures during visit 0, 2-9**

**8.2.1 Patient safety variables and examinations**

Investigator I will assure patient safety during the inclusion visit (visit 0), the first postoperative visit (visit 2) until the end of study (visit 9). He will perform physical examinations and determine the following variables:

- Patients` vital data (blood pressure, heart rate, oxygen saturation)
- Measurement of the urine output every 6 hours until the second postoperative day and the total 24h urine output on the 2^nd^ and 3rd postoperative day.
- Acute kidney failure determination according to the AKIN classification.
- Occurrence of AEs and SAEs will be assessed GCP-conform during each visit and documented in the respective section of the CRF.

**8.2.2 Blood samples and laboratory variables**

Blood samples will be drawn from each patient on the following time-points:

- Baseline sample; between the inclusion (visit 0) and general anesthesia (visit1).
- Postoperatively, once daily on the first 7 postoperative days or until discharge (if earlier), respectively (visit 2-9).
- Additional blood samples will be drawn according to the decision of the investigator or other physicians. This refers to the case of technical problems during the initial visit or in case of loss of the baseline samples before surgery and/ or in case of clinical deviations, which require additional analyses. The following variables will be determined in each blood sample:
- Hematological variables: Erythrocytes, hemoglobin, hematocrit, leucocytes, and platelets.
- Renal variables: creatinine in serum, urea, cystatin C in serum for determination of GFR.
- Coagulation: Quick, INR, pTT
- Liver enzymes: ALT and AST
- Creatine kinase = CK
- Visit 10: Contacting of the family physician or attending urologist, to obtain the GFR and creatinine value, which was probably obtained during the clinical postoperative routine.

**8.3 Evaluation, documentation and analysis of the safety variables**

A specialized investigator will visit then patients daily until the 7^th^ postoperative day or until discharge (if earlier), respectively. An investigator is always available via telephone for events in-between these visits. All patient data will be documented in the CRF and evaluated by the investigator according to the SOP`s (standard operating procedures) of our hospital and the reference table. All data outside of range will be validated by the investigator and marked as clinically significant (cs) or not clinically significant (ncs). Each clinically significant value will be considered as an AE and documented in the adverse event section. The sponsor will be informed about the AEs.

8.4 Adverse events (AE)

An adverse event (AE) is any untoward medical occurrence in a patient or clinical investigation subject administered a pharmaceutical product and which does not necessarily have a causal relationship with this treatment. An adverse event (AE) can therefore be any unfavorable and unintended sign (including an abnormal laboratory finding), symptom, or disease temporally associated with the use of a medicinal (investigational) product, whether or not related to the medicinal (investigational) product. This can include diseases or symptoms, which occur or deteriorate after patient inclusion into the study (GCP-V §3 section 6). If the patients themselves or study personnel do not report AEs, they will be assessed by meticulous clinical examination, assessment of safety variables and a non-suggestive patient interview by the investigator. Furthermore, they will be documented thoroughly. If an investigator assumes an AE, he has to take all measures to make a diagnosis and the corresponding medical treatment. Patients will be observed and treated according to the medical need, until disappearing of symptoms, normalization of the laboratory variables and finding of a sufficient explanation for the observed event. Patients remain in-hospital until they meet the department and hospital standards for discharge. In addition, the investigator will evaluate, the dependence of the AE and the study treatment. The following will be documented in the CRF:

- Kind of AE (signs, symptoms or disease)
- Differentiation (serious/ not serious)
- Date of onset and end
- Intensity
- Causality to the investigational product
- Measures with regard to the study treatment or measures for recovery or improvement of patients` well-being
- Outcome of the event.

Intensity of the symptoms or the adverse event, respectively will be documented as follows:

- **Mild:** Perception of mild symptoms. Symptoms can be ignored easily or disappear, if the attention is diverted to other things.
- **Moderate:** Symptoms cause discomfort, but they are tolerable. They could not be ignored and compromise concentration.
- **Severe:** Severe symptoms, compromising the performance of usual activities.

Sponsor has to be informed about all AEs.

**8.5 Handling of serious adverse events (SAEs)**

- A serious adverse event (SAE) or serious adverse reaction (SAR) are defined as any adverse event or adverse reaction that:
- results in death (fatal) or
- is immediately life-threatening or
- results in persistent or significant disability/incapacity or
- requires or prolongs patient hospitalization or
- induces congenital anomaly/ birth defect.

In case of SAEs/ SARs, all measures described under 8.5 have to be taken and the SAEs/ SARs have to be documented in the CRF. In addition, the sponsor has to be informed immediately (<24h) by the investigator. Sponsor will inform the regulatory body (BfArM) in cases of suspected or confirmed causal relationship with the study treatment within the prescribed period (see 8.6). The investigator will provide a detailed written report after notification of the sponsor.

**8.6 Handling of suspected unexpected serious adverse reaction (SUSAR)**

An adverse reaction (AR) is any untoward and unintended response to an investigational medicinal product related to any dose administered. An unexpected adverse reaction (UAR) is a reaction with causality to the investigational product, which is not consistent with the applicable product information (e.g. summary of product characteristics). Diagnosis of a suspected unexpected serious adverse Reaction (SUSAR) has to be reported to the sponsor according to the guideline 2001/20/EG and the sponsor has to report it within 15 days to the respective Ethics Committee and the responsible authority (BfArM) (§13, section 2 GCP-V). This time-span is reduced to 7 days, if the suspected SUSAR has led to death or is immediately life threatening (§13, section 3 GCP-V).

**8.7 Review of the risk-benefit ratio**

Sponsor has to inform immediately, or within maximum 15 days the respective Ethics Committee and the responsible authority (BfArM) about each issue, which requires a new risk-benefit evaluation of the investigational product. This includes particularly:

- Case reports of expected serious reactions with unexpected outcome.
- Increasing incidence of expected clinically relevant serious reactions.
- Suspected cases of serious unexpected adverse reactions, after study termination.
- Events in connection with the study conduction or development of a investigational product, which could compromise the safety of the study participants.

**9 Statistics**

**9.1 Sample size**

Due to the lack of previous data, we can only calculate the sample size based on estimates. The aim of this pilot-study is to gather data about Xe, which could be used for a sample size calculation for a subsequent study. According to the literature and from own clinical experience we expect a maximum GFR decrease in the control group of 30% (54 ml min^-1^ 1.73 m^-2^). 10% less decrease in the Xe group, corresponding to 36 ml min^-1^ 1.73 m^-2^ in a healthy patient, have to be seen as a clinically significant difference. With a assumed scattering σ of about 80% of the difference of means δ (30 ml min^-1^ 1.73 m^-2^), a power of 1 - β = 80% and a two-sided significance of α = 5 %, we will need 23 patients per group, to see a significant effect. From our study experience we expect a dropout rate of 10%. Therefor, we will include 2 more patients per group. The total patient number in this pilot-study will be 25 patients per group. With this sample size we would be able to generate "positive signals" with 2-sided p-values below 20% for effects with a standardized difference of about δ /σ = 0.6 and a power of 80%. From clinical and economical reasons, a follow-up of this study idea would not be useful in case of significantly lower effects.

**9.2 Patient selection for inclusion into the analysis**

All randomized patients will be included in the analysis.

**9.3 Statistical analysis**

All data will be collected descriptively according to the study protocol. After verification of the data in the SPSS-data base, they will be provided to the biometric institute (IMSIE, Cologne). A covariance-analysis will be used for analysis of the primary outcome GFR, by comparison of the data in the isoflurane- and Xe group for each time-point. The primary outcome, max. variation of the GFR, will be calculated according to 2.3.4. This primary outcome will be primary analyzed by covariance-analysis with the factor group and the covariate baseline value, with a 2-sided Mann- Whitney-Test with the level of 5%. Primary analysis will be performed according to the Intention-to-treat principle (missing values will be replaced by imputations, which will be pre-defined in a statistical analysis plan. This enables a conservative estimate of the effect). Secondary outcomes will be analyzed according to the per protocol analysis. Other variables will be analyzed descriptive and explorative with the common 2-sample T-test. Descriptive analysis for baseline and outcome variables will be performed with the usual measured values and parameters, graphics and tests (p-values) depending on the scale levels of the variables.

**9.4 Procedures for missing data**

All possible effort will be performed, to reduce missing data. An investigator or study nurse will check the completed CRFs before statistical analysis. Missing data will be identified and if possible entered from the source data of the patient. Remaining missing data will be analyzed according to the Intention-to-treat principle.

**9.5 Analysis procedures for deviations from the statistical analysis plan**

Each deviation from the original statistical analysis plan will be described in the protocol and the final report.

**10 Data entry and data management**

**10.1 Data entry**

The respective investigator and study nurse will perform data collection and psodonymization. Only these persons, the monitor and an auditor will have access to the patient files. Pseudonymization will be performed via center number and a consecutively increasing number. The respective physician is obliged to ensure identification of a particular person at any time via the registry-list. Therefore, he will have a identification log with the full patient names. If patient identification is necessary, due to safety reasons, then all respective persons underlie professional secrecy. The investigator is responsible for the correctness and complete documentation of the study data.

**10.2 Data storage**

Study CRFs and the data of the screened but not included patients will be sored together with the completed study documents (study protocol, ethical approval, BfArM-approval) in a locked cabinet with restricted access in the Department of Anesthesiology for 15 years (prescribed by law).

**10.3 Data management**

**10.3.1 Access to source data**

Investigators are obliged to permit authorized third persons access to the source data, according to the legal requirements for quality and protection of data and protection of the study conduction in this center. This includes monitors, auditors, independent Ethical Committees, and authorities. These persons are sworn to secrecy.

**10.3.2 Monitoring**

The sponsor will provide a monitor, who will check all signed patient informed consents and randomly check the accordance of the original data and the CRFs. The investigator is obliged to permit study specific monitoring and enable direct access to the patient files and the source data. Data will be double entered from the paper CRF into the database by to independent student assistants. The responsible biometrician will check both data files for concordance via a coded algorithm. The monitor will be informed if there is discordance between these data files, to check the data in the paper CRF. The investigator will bi inquired for all missing and implausible data, which he has to remove or explain. The student assistants will perform changes in the SPSS database. Data entry and data corrections have to be signed with the name of the respective person, date and time. Monitor will also check intervention performance.

**10.4 Final report**

The principle investigator will create and provide to the sponsor a final report after study data analysis, according to the ICH-GCP guidelines.

**11 Quality control and quality assurance**

**11.1 Quality control**

This study will be conducted in accordance with the ICH Guideline for Good Clinical Practice (GCP) E6, June 1996; the CPMP/ICH/135/95, September 1997; and the legal regulations, to assure a correct, valid and consistent data collection and to guarantee the usage of standardized operating procedures (SOPs).

**11.2 Quality assurance**

The following defaults will be used for quality control:

- Feedback meetings and exercises, for preparation of the investigators for the study SOPs.
- Monitoring of included patients according to the safety criteria.
- The sponsor may visit the study site at any time and inspect the study progress, study conduction, the original data and the CRFs.
- Matching of the original source data and the CRFs.
- Double entry of the paper CRF data by two to independent student assistants into the SPSS database. Verification of these entries by the responsible biostatistician via a coded algorithm.

**12 Ethical and administrative aspects**

**12.1 Explanation of the planned examinations on humans or human material or animals**

This pilot-study is a clinical investigation of an approved drug. All planned investigations in this pilot-study will be conducted in accordance with the medicine law (current version of 2011) and the GCP guideline (current version 2006) based on the Helsinki declaration. This implies an ethical approval, the approval of the competent authority and the non-public registration at the European Medicines Agency (EMEA). We will register this study in a public registry at clinical trials.gov in addition. Patients will be verbally and written informed about the aims, the conduction and the benefit of this study including the personnel benefit and risk. It has to be noted in written form, before enrolment into the study, that inclusion into the study was voluntary and after information and weighing. The patient is allowed to withdraw from study without reasons at any time. Randomization ensures the same chance to be allocated to one of the two groups. Pseudonymization of the patients and their data with a study number preserves data protection toward persons, who are not allowed to see these data (biometrician, data manager etc.). Patient selection and treatment are described under 4 and 5. Surgery and anesthesia are conducted according to the clinical standards and guidelines. A SOP, where only the drug is different will be established for the study anesthesia in both groups (Xe and control). Patients will be closely monitored during anesthesia conduction; safety data like anesthesia data and vital data will be assessed in short intervals as described above. Highest priority has the well being of the patients. If a threatening or occurred damage for the patient appears at any time-point of the study conduction, independently of a causal relationship or not, the study participation will be terminated and an adequate diagnosis and therapy will be induced immediately. All patients will receive a physical examination at the study end (planned/ individual) and will receive further diagnosis and therapy if the result was conspicuous. Summarizing, the investigational product Xe is a safe for anesthesia already approved drug. The risk for study participation does not exceed the risk for general anesthesia for PN. The renal tumor resection tissue will undergo histopathological examination in paraffin sections and a graduation of the tumor stage according to the clinical routine. In addition, there will be produced additional paraffin sections, which will probably be used in this pilot-study for HIF-1α determination in the healthy surrounding tissue by immunohistochemistry. An additional storage exceeding the clinical routine is not planned.

**12.2 Ethical approval**

Ethical approval will be applied before consultation of the regulatory authority and before study start according to GCP-V:

Ethics Committee of the Medical Faculty RWTH Aachen

Chairperson: Prof. Dr. med. G. Schmalzing

University Hospital Aachen

Pauwelsstr. 30, 52074 Aachen, Germany

Tel.: +49-241-8089963

Fax.: +49-241-8082012

E-mail: ekaachen@ukaachen.de

**12.3 Approval of the regular authority**

The approval from the regular authority will be applied according to GCP-V from:

Bundesinstitut für Arzneimittel und Medizinprodukte BfArM

Kurt-Georg-Kiesinger-Allee 3

53175 Bonn

Germany

Tel.: +49 228 207-30

Fax: +49 228 207-5207

E-mail: poststelle@bfarm.de

**12.4 Protocol amendments**

Any modification of the predefined trial conditions in the study protocol is not planned, for assurance of proper data analysis. Any modifications to the study protocol that may impact the conduct of the study are possible in exceptional cases. Modifications will only take place in coordination of the investigator with the sponsor. All modifications of the study procedure have to be made in written form in the study protocol, stating the reasons, and signed by all responsible persons. Each amendment becomes part of the study protocol. Substantial amendments (e.g. change of the drug dose and/ or other significant changes, which may influence the safety of the study participants), have to be approved by the respective Ethics Committee and/ or the respective regular authority, as well as the patients. The amendment has to be provided to the regular authority (BfArM).

**12.5 End of study**

End of study will be reported by the sponsor to the regular authority within 90 days, in accordance with § 12 and 13 GCP-V.

**12.6 Final report**

The sponsor will provide the final report to the regular authority within one year, in accordance with § 13 GCP-V.

**13 Documentation**

**13.1 Data collection and management**

**13.1.1 Source data**

All data from the study related examinations and surveys will directly be documented in the case report forms (CRFs) and considered as source data. Additional information from not-study related examinations, or data from the patient file (like ECG and laboratory data print-outs), respectively, will be copied, considered as source data and filed in the CRFs.

**13.1.2 Case Report Forms (CRF`s)**

Two CRFs will be created for each patient. One for the data of investigator I, one CRF for data collected by investigator II. CRFs will be filled in manually with a water insoluble pen. CRFs will be stored according to point 10.2. The principle investigator will create a final report, after study data analysis, according to the ICH-GCP guidelines and provide it to the sponsor.

**13.2 Digitalization of study data**

The investigator or study nurse will check the completed CRFs with regard to completeness and correctness. Data will be double entered from the paper CRF into the SPSS database by to independent student assistants, to check the concordance via a coded algorithm. This will be followed by the statistical analysis.

**13.3 Storage of study data**

The sponsor of this study - Clinical Trials Center Aachen (CTC-A), University Hospital Aachen, Pauwelsstr. 30, 52074 Aachen, Germany - is obliged, according to the ICH Topic E6 (R1) Guideline for Good Clinical Practice (CPMP/ICH/135/95), to store all important documents (described in 8.2-8.6) for 15 years. The final report has to be stored for 5 years. Documents from a prematurely terminated study have to be stored for 2 years.

Above-mentioned important documents, will be sored by the principle investigator) in a locked cabinet with restricted access in the Department of Anesthesiology for 15 years (prescribed by law). The principle investigator is obliged to prevent an erroneous or premature destruction of these documents.

**14 Formal regulations**

This study will be performed in concordance with the study protocol, the German medical act (AMG), the ICH-GCP guidelines, Declaration of Helsinki, and the publication of principles for correctly conducted clinical trials with medical products.

**14.1 Funding**

The German Research Foundation (DFG) and the Department of Anesthesiology (University Hospital Aachen, director: Univ.-Prof. Dr. Rolf Rossaint) will fund this study. All detailed, financial aspects for this study will be noted in separate agreements.

**14.2 Study insurance**

According to the regulatory requirements (§40 section 1b) we do not need a special insurance for the study participants. The patients are insured by the business liability insurance of the University Hospital Aachen, in case of strict liability.

**15 Publication agreements**

Un-blinding will occur after data analysis by the responsible biostatistician. The principle investigator and the biometrician will create a final report and a manuscript. At least it will be send to a peer-reviewed journal for publication.

The principle investigator will create a report each year and after study termination for the DFG.

**16 Signatures**

**17 References**

1. Bono AV, Lovisolo JA. Renal cell carcinoma--diagnosis and treatment: state of the art. Eur Urol. 1997;31 Suppl 1:47-55.
2. Hollingsworth JM, Miller DC, Daignault S, Hollenbeck BK. Rising incidence of small renal masses: a need to reassess treatment effect. J Natl Cancer Inst. 2006;98:1331-1334.
3. Jemal A, Siegel R, Xu J, Ward E. Cancer statistics, 2010. CA Cancer J Clin. 2010;60:277-300.
4. Kane CJ, Mallin K, Ritchey J, Cooperberg MR, Carroll PR. Renal cell cancer stage migration: analysis of the National Cancer Data Base. Cancer. 2008;113:78-83.
5. Ljungberg Cowan NC, Hanbury DC, Hora M, Kuczyk MA, Merseburger AS, Patard JJ, Mulders PF, Sinescu IC; European Association of Urology Guideline Group. EAU guidelines on renal cell carcinoma: the 2010 update. Eur Urol. 2010;58:398-406.
6. Han JS, Huang WC. Impact of Kidney Cancer Surgery on Oncologic and Kidney Functional Outcomes. Am J Kidney Dis. 2011;58:846-854.
7. Lane BR, Russo P, Uzzo RG, Hernandez AV, Boorjian SA, Thompson RH, Fergany AF, Love TE, Campbell SC. Comparison of cold and warm ischemia during partial nephrectomy in 660 solitary kidneys reveals predominant role of nonmodifiable factors in determining ultimate renal function. J Urol. 2011;185:421- 7.
8. Yossepowitch O, Eggener SE, Serio A, Huang WC, Snyder ME, Vickers AJ, Russo P. Temporary renal ischemia during nephron sparing surgery is associated with short-term but not long-term impairment in renal function. J Urol. 2006;176:1339-43; discussion 1343.
9. Simmons MN, Schreiber MJ, Gill IS. Surgical renal ischemia: a contemporary overview. J Urol. 2008;180:19-30.
10. Coburn M, Kunitz O, Baumert J-H, Hecker K, Haaf S, Zühlsdorf A, Beeker T, Rossaint R. Randomized controlled trial comparing hemodynamic and recovery effects of xenon. Br J Anaesth 2005;94:198-202.
11. Rossaint R, Reyle-Hahn M, Schulte Am Esch J, Scholz J, Scherpereel P, Vallet B, Giunta F, Del Turco M, Erdmann W, Tenbrinck R, Hammerle AF, Nagele P; Xenon Study Group. Multicenter randomized comparison of the efficacy and safety of xenon and isoflurane in patients undergoing elective surgery. Anesthesiology. 2003;98:6-13.
12. Hobbs C, Thoresen M, Tucker A, Aquilina K, Chakkarapani E, Dingley J. Xenon and hypothermia combine additively, offering long-term functional and histopathologic neuroprotection after neonatal hypoxia/ischemia. Stroke 2008;39:1307-1313
13. Coburn M, Maze M, Franks NP. The neuroprotective effects of xenon and helium in an in vitro model of traumatic brain injury. Crit Care Med. 2008;36:588-95.
14. Hein M, Roehl AB, Baumert JH, Bleilevens C, Fischer S, Steendijk P, Rossaint R. Xenon and isoflurane improved biventricular function during right ventricular ischemia and reperfusion. Acta Anaesthesiol Scand. 2010;54:470-8.
15. Weber NC, Stursberg J, Wirthle NM, Toma O, Schlack W, Preckel B. Xenon preconditioning differently regulates p44/42 MAPK (ERK 1/2) and p46/54 MAPK (JNK 1/2 and 3) in vivo. Br J Anaesth. 2006;97:298-306.
16. Mio Y, Shim YH, Richards E, Bosnjak ZJ, Pagel PS, Bienengraeber M. Xenon preconditioning: the role of prosurvival signaling, mitochondrial permeability transition and bioenergetics in rats. Anesth Analg. 2009;108:858-66.
17. Schwiebert C, Huhn R, Heinen A, Weber NC, Hollmann MW, Schlack W, Preckel B. Postconditioning by xenon and hypothermia in the rat heart in vivo. Eur J Anaesthesiol. 2010;27:734-9.
18. Ma D, Lim T, Xu J, Tang H, Wan Y, Zhao H, Hossain M, Maxwell PH, Maze M. Xenon preconditioning protects against renal ischemic-reperfusion injury via HIF- 1alpha activation. J Am Soc Nephrol. 2009;20:713-20.
19. Rizvi M, Jawad N, Li Y, Vizcaychipi MP, Maze M, Ma D. Effect of noble gases on oxygen and glucose deprived injury in human tubular kidney cells. Exp Biol Med (Maywood). 2010;235:886-91.
20. Irani Y, Pype JL, Martin AR, Chong CF, Daniel L, Gaudart J, Ibrahim Z, Magalon G, Lemaire M, Hardwigsen J. Noble gas (argon and xenon)-saturated cold storage solutions reduce ischemia-reperfusion injury in a rat model of renal transplantation. Nephron Extra. 2011;1: 272-82.
21. Stoppe C. Fahlenkamp AV, Rex S, Veeck NC, Gozdowsky SC, Schälte G, Autschbach R, Rossaint R, Coburn M: Feasibility and safety of xenon compared to sevoflurane anaesthesia in coronary surgical patients – a randomized controlled pilot study. In revision at Br J Anesth 2012.
22. Stoppe C, Werker T, Rossaint R, Dollo F, Lue H, Wonisch W, Menon A, Götzenich A, Bruells CS, Coburn M, Kopp R, Bucala R, Bernhagen J, Rex S. What ist he significance of perioperativ release of macrophage migration inhibitory factor in cardiac surgery? Antioxid Redox Signal. 2012 Nov 19.
23. Roggenbach J, Morath C. Postoperatives Nierenversagen. Der Nephrologe 02/2009; 4(2):118-127
24. Zhang L, Huang H, Cheng J, Liu J, Zhao H, Vizcaychipi MP, Ma D. Pre-treatment with isoflurane ameliorates renal ischemic-reperfusion injury in mice. Life Sci. 2011;88:1102-7.
25. Lee HT, Kim M, Kim J, Kim N, Emala CW. TGF-beta1 release by volatile anesthetics mediates protection against renal proximal tubule cell necrosis. Am J Nephrol. 2007;27:416-24.
26. Lee HT, Ota-Setlik A, Fu Y, Nasr SH, Emala CW. Differential protective effects of volatile anesthetics against renal ischemia-reperfusion injury in vivo. Anesthesiology. 2004;101:1313-24.
27. Bito H, Ikeuchi Y, Ikeda K. Effects of low-flow sevoflurane anesthesia on renal function: comparison with high-flow sevoflurane anesthesia and low-flow isoflurane anesthesia. Anesthesiology. 1997;86:1231-7.
28. Teixeira S, Costa G, Costa F, da Silva Viana J, Mota A. Sevoflurane versus isoflurane: does it matter in renal transplantation? Transplant Proc. 2007;39:2486- 8.
29. Ko JS, Kim G, Shin YH, Gwak MS, Kim GS, Kwon CH, Joh JW. The effects of desflurane and isoflurane on hepatic and renal functions after right hepatectomy in living donors. Transplant Proc. 2012;44:442-4.
30. Story DA, Poustie S, Liu G, McNicol PL. Changes in plasma creatinine concentration after cardiac anesthesia with isoflurane, propofol, or sevoflurane: a randomized clinical trial. Anesthesiology. 2001;95:842-8.
31. Coll E, Botey A, Alvarez L, Poch E, Quintó L, Saurina A, Vera M, Piera C, Darnell A. Serum cystatin C as a new marker for noninvasive estimation of glomerular filtration rate and as a marker for early renal impairment. Am J Kidney Dis. 2000;36:29-34.
32. Larsson A, Malm J, Grubb A, Hansson L-O. Calculation of glomerular filtration rate expressed in mL/min from plasma cystatin C values in mg/L. Scand J Clin Lab Invest 2004; 64: 25–30.
33. Herget-Rosenthal S, Pietruck F, Volbracht L, Philipp T, Kribben A. Serum cystatin C--a superior marker of rapidly reduced glomerular filtration after uninephrectomy in kidney donors compared to creatinine. Clin Nephrol. 2005;64:41-6.
34. Mehta RL, Kellum JA, Shah SV, Molitoris BA, Ronco C, Warnock DG, Levin A, Acute Kidney Injury Network. Acute Kidney Injury Netw
35. Ricci Z, Cruz D, Ronco C. The RIFLE criteria and mortality in acute kidney injury: A systematic review. Kidney Int 2008; 73: 538-546.
