# Supplementary material for: Renal function following xenon anesthesia for partial nephrectomy—An explorative analysis of a randomized controlled study
Source: PLoS One. 2017 Jul 18;12(7):e0181022. doi: 10.1371/journal.pone.0181022 (PMC5515428; doi:10.1371/journal.pone.0181022)
Supplement: S2 Table — (DOCX) [file pone.0181022.s005.docx]

**S2 Table. Pre-existent chronic disease.**

| **Group** | **Total (n=46)** | **Isoflurane (n=23)** | **Xenon (n=23)** | ***P*-value**^a^ |
| --- | --- | --- | --- | --- |
| Cardiac disease [n] | | | | |
| Cardiac insufficiency: NYHA II | 7 | 3 | 4 | 1.0 |
| CAD | 2 | 1 | 1 | 1.0 |
| Myocardial Infarction | 2 | 1 | 1 | 1.0 |
| Pulmonary disease [n] | | | | |
| COPD/Asthma | 0/0 | 0/0 | 0/0 | 1.0 |
| Diabetes [n] | | | | |
| NIDDM/IDDM | 7/1 | 3/0 | 4/1 | 0.699 |
| Renal Insufficiency | 0 | 0 | 0 | 1.0 |
| Vascular Disease [n] | | | | |
| Hypertension | 24 | 12 | 12 | 1.0 |
| Stroke | 1 | 1 | 0 | 1.0 |
| PAOD | 0 | 0 | 0 | 1.0 |
| Abuse [n] | | | | |
| Alcohol/Drugs | 0/0 | 0/0 | 0/0 |  |

CAD, coronary artery disease; COPD, chronic obstructive pulmonary disease; IDDM, insulin dependent diabetes mellitus; n, number; NIDDM, non insulin dependent diabetes mellitus; NYHA, New York Heart Association; PAOD, peripheral artery occlusive disease. ^a^ *P*-values were calculated using Fisher's exact test. Data are presented as numbers of patients.
