# Supplementary material for: Renal function following xenon anesthesia for partial nephrectomy—An explorative analysis of a randomized controlled study
Source: PLoS One. 2017 Jul 18;12(7):e0181022. doi: 10.1371/journal.pone.0181022 (PMC5515428; doi:10.1371/journal.pone.0181022)
Supplement: S3 Table — (DOCX) [file pone.0181022.s006.docx]

**S3 Table.** **Postoperative renal function.**

| **Analysis** | **Intention to Treat** | | | **Per Protocol** | | |
| --- | --- | --- | --- | --- | --- | --- |
| **Group** | **Isoflurane (n=23)** | **Xenon (n=23)** | ***P*-value**^a^ | **Isoflurane (n=19)** | **Xenon (n=22)** | ***P*-value**^a^ |
| Baseline GFR [ml min^-1^ 1,73 cm^-^²] | 90.2 ± 14.0, 85.6 (16) | 86.7 ± 17.8, 88.0 (26.4) | 0.668 | 92.0 ± 12.7, 95.1 (15.9) | 86.7 ± 18.2, 88.3 (26.4) | 0.464 |
| Max GFR decrease [%] | 21.3 | 10.8 | - | 18.1 | 9.4 | - |
| Max GFR decrease [ml min^-1^ 1,73 cm^-^²] | 23.6 ± 18.6, 22.1 (39.8) | 12.4 ± 17.0, 10.8 (27.6) | 0.032 | 19.7 ± 17.7, 17.0 (26.3) | 10.9 ± 15.7, 10.7 (24.8) | 0.084 |
| Surgery with hilar clamping | **(n=8)**, 27.0 ± 18.8, 24.6 (37.9) | **(n=10)**, 18.2 ± 20.3, 21.1 (37.1) | 0.053 | **(n=8)**, 27.0 ± 18.8, 24.6 (37.9) | **(n=9)**, 15.2 ± 19.0, 14.6 (36.8) | 0.233 |
| Surgery without hilar clamping | **(n=15)**, 21.8 ± 18.9, 17.0 (37.2) | **(n=13),** 7.9 ± 13.0, 8.4 (14.7) | 0.174 | **(n=11)**, 14.4 ± 15.5, 8.7 (20.3) | **(n=13)**, 7.9 ± 13.0, 8.4 (14.7) | 0.268 |

GFR, glomerular filtration rate; max, maximum; n, number; POD, postoperative day. ^a^ *P*-values are from Mann-Whitney *U*-test, except for maximum GFR decrease (ANOVA). Data are presented as means ± standard deviation, median (interquartile range) or as percentage.
