# Supplementary material for: Renal function following xenon anesthesia for partial nephrectomy—An explorative analysis of a randomized controlled study
Source: PLoS One. 2017 Jul 18;12(7):e0181022. doi: 10.1371/journal.pone.0181022 (PMC5515428; doi:10.1371/journal.pone.0181022)
Supplement: S4 Table — (DOCX) [file pone.0181022.s007.docx]

**S4 Table. Key intervention data.**

| **Analysis** | **Intention to Treat** | | | **Per Protocol** | | |
| --- | --- | --- | --- | --- | --- | --- |
| **Group** | **Isoflurane (n=23)** | **Xenon (n=23)** | ***P*-value**^a^ | **Isoflurane (n=19)** | **Xenon (n=22)** | ***P*-value**^a^ |
| Duration of surgical intervention [min] | 147.7 ± 57.6, 139 (99) | 136.8 ± 58.8, 149 (93) | 0.575 | 145.6 ± 58.8, 139 (99) | 136.0 ± 60.0, 142.5 (93) | 0.610 |
| Kidney manipulation time [min] | 28.8 ± 17.7, 24 (15) | 25.0 ± 22.0, 19 (16) | 0.084 | 26.9 ± 15.2, 23 (17) | 25.6 ± 22.3, 19 (16) | 0.193 |
| Hilar clamping time [min] | 12.4 ± 5.3 **(n=8),** 11 (3.5) | 16.3 ± 9.0 **(n=10),** 14.5 (9) | 0.315 | 12.4 ± 5.3 **(n=8),** 11 (3.5) | 16.7 ±9.4 **(n=9)**, 15 (9) | 0.334 |
| Exposure to anesthetic before kidney manipulation [min] | 118.1 ± 42.8, 109 (74) | 86.6 ±44.8, 77.5 (57) | 0.014 | 120.2 ± 45.8, 108 (79) | 85.3 ±45.5, 76 (51) | 0.013 |
| Exposure to anesthetic after kidney manipulation [min] | 43.0 ± 16.9, 43 (26) | 46.2 ± 22.4, 38 (27) | 0.829 | 42.3 ± 17.0, 43 (22) | 46.7 ± 22.8, 39 (27) | 0.704 |
| Total infusion of crystalloids [ml] | 2130.4 ± 907.3, 2000 (1500) | 2037.0 ± 817.2, 2000 (1000) | 0.702 | 2184.2 ± 974.8, 2000 (1500) | 2015.9 ± 830.0, 2000 (1000) | 0.511 |
| Total infusion of colloids [ml] | 833.3 ± 577.4 **(n=3)**, 500 (1000) | 500 ± 0 **(n=4)**, 500 (0) | 0.248 | 833.3 ± 577.4 **(n=3)**, 500 (1000) | 500 ± 0 **(n=4)**, 500 (0) | 0.248 |
| Total blood loss [ml] | 348.6 ± 376.8, 150 (300) | 231.6 ± 192.4, 220 (270) | 0.361 | 345.8 ± 389.9, 150 (350) | 231.6 ± 192.4, 220 (270) | 0.428 |
| Total urine output [ml] | 279.9 ± 409.1, 120 (176) | 234.6 ± 206.5, 170 (200) | 0.308 | 295.3 ± 418.7, 140 (182) | 229.5 ± 211.7, 150 (150) | 0.496 |

Min, minutes; n, number. ^a^ *P*-values are from Mann-Whitney *U*-test. Data are presented as mean ± standard deviation, median (interquartile range).
