# Supplementary material for: Renal function following xenon anesthesia for partial nephrectomy—An explorative analysis of a randomized controlled study
Source: PLoS One. 2017 Jul 18;12(7):e0181022. doi: 10.1371/journal.pone.0181022 (PMC5515428; doi:10.1371/journal.pone.0181022)
Supplement: S5 Table — (DOCX) [file pone.0181022.s008.docx]

**S5 Table.** **Histological and pathological analyses.**

| **Analysis** | **Intention to Treat** | | | **Per Protocol** | | |
| --- | --- | --- | --- | --- | --- | --- |
| **Group** | **Isoflurane (n=23)** | **Xenon (n=23)** | ***P*-value**^a^ | **Isoflurane (n=19)** | **Xenon (n=22)** | ***P*-value**^a^ |
| Renal tissue excision volume [cm³] | 312.2 ± 773.2, 61.2 (173.7) | 149.8 ± 217.1, 46.3 (231.3) | 0.829 | 210.0 ± 688.1, 32 (81.1) | 134 ± 210.2, 39.4 (89.4) | 0.416 |
| Renal tissue excision weight [g] | 194.1 ± 287.8, 109 (178.6) | 53.2 ± 73.1, 22.5 (58.9) | 0.122 | 85.8 ± 106.6, 50.5 (127.1) | 39.4 ± 49.7, 22 (58.3) | 0.407 |
| Tumor size [cm] | 4.2 ± 2.5, 4.1 (3.6) | 3.6 ± 2.0, 3.4 (2.2) | 0.510 | 3.4 ± 1.8, 3.2 (3) | 3.6 ± 2.1, 3.5 (2.4) | 0.799 |
| Tumor histological classification |  |  | 0.639 |  |  | 0.870 |
| Renal Carcinoma [n] (%) | 19 (82.6) | 15 (65.2) |  | 15 (78.9) | 14 (63.6) |  |
| Oncocytoma [n] (%) | 0 (0) | 2 (8.7) |  | 0 (0) | 2 (9.1) |  |
| Angiomyolipoma [n] (%) | 2 (8.7) | 3 (13) |  | 2 (10.5) | 3 (13.6) |  |
| Adenoma [n] (%) | 1 (4.3) | 1 (4.3) |  | 1 (5.3) | 1 (4.5) |  |
| Cyst [n] (%) | 1 (4.3) | 2 (8.7) |  | 1 (5.3) | 2 (9.1) |  |

n, number. ^a^ *P*-values are from Fisher's exact test (qualitative data) or Mann-Whitney *U*-test (quantitative data), respectively. Data are presented as mean ± standard deviation, median (interquartile range) or number and percentage.
