# Supplementary material for: Renal function following xenon anesthesia for partial nephrectomy—An explorative analysis of a randomized controlled study
Source: PLoS One. 2017 Jul 18;12(7):e0181022. doi: 10.1371/journal.pone.0181022 (PMC5515428; doi:10.1371/journal.pone.0181022)
Supplement: S6 Table — (DOCX) [file pone.0181022.s009.docx]

**S6 Table.** **Adverse events.**

| **Analysis** | **Intention to Treat** | | | **Per Protocol** | | |
| --- | --- | --- | --- | --- | --- | --- |
| **Group** | **Isoflurane (n=23)** | **Xenon (n=23)** | ***P*-value**^a^ | **Isoflurane (n=19)** | **Xenon (n=22)** | ***P*-value**^a^ |
| Adverse events [n] | 2.3 ± 1.4, 2 (2) | 1.0 ± 1.0, 1 (2) | 0.001 | 2.1 ± 1.4, 1 (2) | 0.9 ± 0.9, 1 (1) | 0.003 |
| Intraoperative hypotension, requiring catecholamines [y/n] (%) | 19/4 (82.6/17.4) | 10/13 (43.5/56.5) | 0.013 | 16/3 (84.2/15.8) | 9/13 (40.9/59.1) | 0.009 |
| Nausea [y/n] (%) | 7/16 (30.4/69.6) | 3/20 (13.0/87.0) | 0.284 | 5/14 (26.3/73.7) | 3/19 (13.6/86.4) | 0.436 |
| Vomiting [y/n] (%) | 2/21 (8.7/91.3) | 1/22 (4.3/95.7) | 1.000 | 1/18 (5.3/94.7) | 1/21 (4.5/95.5) | 1.000 |
| Acute kidney injury [y/n] (%), AKIN 1/2 | 11/12 (47.8/52.2), 7/4 | 7/16 (30.4/69.6), 6/1 | 0.387 | 8/11 (42.1/57.9), 6/2 | 6/16 (27.3/72.7), 6/0 | 0.320 |
| Anemia, requiring PRBC transfusion [y/n] (%) | 4/19 (17.4/82.6) | 0/23 (0/100) | 0.109 | 4/15 (21.1/78.9) | 0/22 (0/100) | 0.038 |
| Post-operative bleeding/ hematoma [y/n] (%) | 4/19 (17.4/82.6) | 0/23 (0/100) | 0.109 | 4/15 (21.1/78.9) | 0/23 (0/100) | 0.038 |
| Surgical Revision [y/n] (%) | 1/22 (4.3/95.7) | 0/23 (0/100) | 1.000 | 1/18 (5.3/94.7) | 0/22 (0/100) | 0.463 |
| Nephrectomy [y/n] | 4 | 1 | 0.346 | 0 | 0 | - |
| Serious adverse event [n] | 0 | 0 | - | 0 | 0 | - |

AKIN, Acute Kidney Injury Network; n, number; PBRC, packed red blood cells; y/n, yes/ no. ^a^ *P*-values are from Fisher's exact test (qualitative data) or Mann-Whitney *U*-test (quantitative data), respectively. Data are presented as mean ± standard deviation, median (interquartile range) or number and percentage.
