# Supplementary material for: Renal function following xenon anesthesia for partial nephrectomy—An explorative analysis of a randomized controlled study
Source: PLoS One. 2017 Jul 18;12(7):e0181022. doi: 10.1371/journal.pone.0181022 (PMC5515428; doi:10.1371/journal.pone.0181022)
Supplement: S7 Table — (DOCX) [file pone.0181022.s010.docx]

**S7 Table.** **Hospital length of stay and longer-term kidney function.**

| **Analysis** | **Intention to Treat** | | | **Per Protocol** | | |
| --- | --- | --- | --- | --- | --- | --- |
| **Group** | **Isoflurane (n=23)** | **Xenon (n=23)** | ***P*-value**^a^ | **Isoflurane (n=19)** | **Xenon (n=22)** | ***P*-value**^a^ |
| Length of hospital stay [d] | 6.1 ± 1.8, 6 (2) | 6.5 ± 4.1, 5 (2) | 0.599 | 6.2 ± 1.8, 6 (2) | 6.5 ± 4.2, 5 (2) | 0.559 |
| Renal function at 3-6 months | **Isoflurane (n=13)** | **Xenon (n=15)** |  | **Isoflurane (n=11)** | **Xenon (n=15)** |  |
| Serum creatinine [mg dl^-1^] | 1.0 ± 0.3, 1 (0.4) | 1.0 ± 0.2, 1 (0.4) | 0.695 | 1.0 ± 0.3, 1.0 (0.5) | 1.0 ± 0.2, 1.0 (0.4) | 0.736 |
| GFR (creatinine) [ml min^-1^ 1.73m^-^²] | 75.0 ±18.1, 70 (23.7) | 82.1 ± 16.0, 78.4 (19.6) | 0.300 | 78.9 ±16.6, 76.8 (24.4) | 82.1 ± 16.0, 78.4 (19.6) | 0.622 |
| Urea [mg dl^-1^] | 34.5 ± 11.2, 35 (8.6) | 36.2 ± 11.5, 34 (17.7) | 0.879 | 34.5 ± 11.2, 35 (8.6) | 36.2 ± 11.5, 34 (17.7) | 0.879 |

GFR, glomerular filtration rate; n, number. ^a^ *P*-values are from Mann-Whitney *U*-test. Data are presented as mean ± standard deviation, median (interquartile range).
